# Gold-catalyzed formation of pyrrolo- and indolo-oxazin-1-one derivatives: The key structure of some marine natural products

**DOI:** 10.3762/bjoc.11.101

**Published:** 2015-05-28

**Authors:** Sultan Taskaya, Nurettin Menges, Metin Balci

**Affiliations:** 1 Middle East Technical University, Department of Chemistry, Ankara, Turkey, 06800; 2 Yüzüncü Yil University, Faculty of Pharmacy, Van, Turkey, 65100

**Keywords:** alkyne cyclization, gold-catalyzed reaction, indolo-oxazin-1-one, marine natural products, pyrrolo-oxazin-1-one

## Abstract

Various *N*-propargylpyrrole and indolecarboxylic acids were efficiently converted into 3,4-dihydropyrrolo- and indolo-oxazin-1-one derivatives by a gold(III)-catalyzed cyclization reaction. Some of the products underwent TFA-catalyzed double bond isomerization and some did not. Cyclization reactions in the presence of alcohol catalyzed by Au(I) resulted in the formation of hemiacetals after cascade reactions.

## Introduction

**Figure 1:**
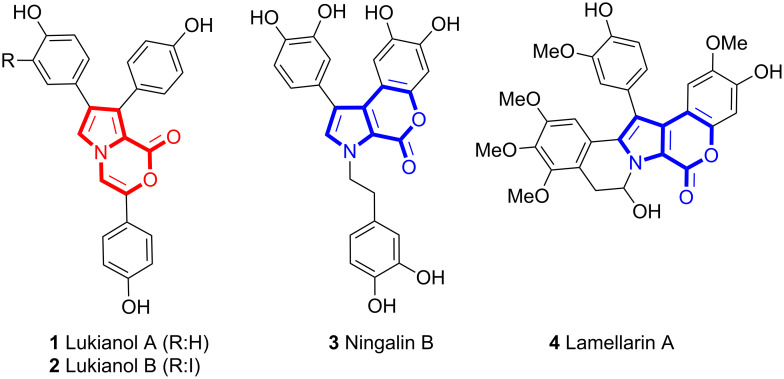
Structures of some marine natural products **1–4**.

Pyrrole-containing heterocycles are widely distributed within a large number of natural products and biologically active molecules [1]. These compounds possess a wide range of biological and pharmacological activities [2–5]. The oxazinone moiety is also frequently found in compounds displaying biological activities [6–7]. Pyrrolo-oxazinone structures with an oxazinone ring fused to a pyrrole are found in marine natural products, such as lukianol A (**1**) and lukianol B (**2**) (Figure 1) [8–10]. Lukianol B (**2**) was found to be the most potent human aldose reductase (h-ALR2) inhibitor in thousands of marine natural products screened [11]. The ningalin B alkaloid **3**, having an isomeric pyrrolo-oxazinone skeleton, possesses antitumor and multidrug resistance reversal activity [12–14]. Lamellarins **4** with a pyrrolo-oxazinone substructure are also marine natural products and they inhibit the proliferation of cancer cells and therefore are promising candidates for anticancer drugs [15–20].

**Figure 2:**
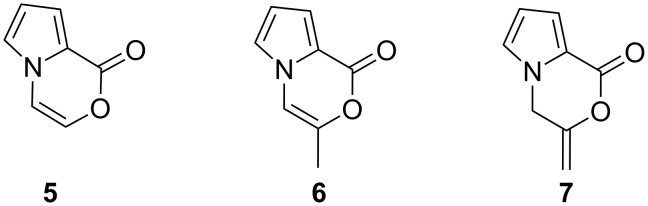
Structures **5–7**.

The design and synthesis of pharmaceutically relevant building blocks has always been a significant goal in organic synthesis. Moreover, modification of natural products is also an important approach to identify promising anticancer agents. Especially due to the bioactivity of lukianols, many groups accomplished the synthesis of these marine products and their analogues [21–26]. Despite the general and widespread interest in these structures, the core structure, 1*H*-pyrrolo[2,1-*c*][1,4]oxazin-1-one (**5**), is not described in the literature. However, there are relatively few synthetic routes leading to the substituted core structure **6** (Figure 2), which was generated by treatment of methyl 2-pyrrole-carboxylate with chloroacetone [2,27–30]. This core structure is also a very important intermediate for the synthesis of pyrrolo-pyrazinones as well as for pyrrolo-pyrazines [31]. Recently, Wang et al. reported the base-catalyzed intramolecular cyclization of alkynyl alcohols at high temperatures to give 1,4-oxaza heterocycles [32].

In this manuscript we envisaged a synthetic strategy leading to the core skeleton of marine products **6** and **7** (Figure 2) or their analogues with short reaction times and an atom-economic process using a gold-catalyzed alkyne cyclization reaction as the key step.

**Scheme 1:**
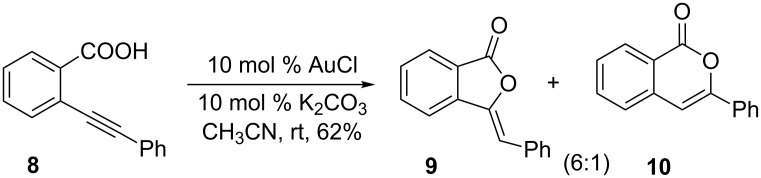
Intramolecular gold(I)-catalyzed cyclization reaction of **8** to give **9** and **10**.

Pyrrole and indole, bearing one (substituted) propargyl and carboxy group was initially designed as model substrate for the construction of **6** and **7**. Gold-catalyzed intramolecular cyclization of enyene carboxylic acids has been reported in the literature to give the corresponding lactones [33–40]. For example, gold(I)-catalyzed reaction of 2-(phenylethynyl)benzoic acid (**8**) yielded the *exo-* and *endo-*dig cyclization products, lactones **9** and **10** in 62% yield in a ratio of 6:1 (Scheme 1) [41].

## Results and Discussion

**Scheme 2:**
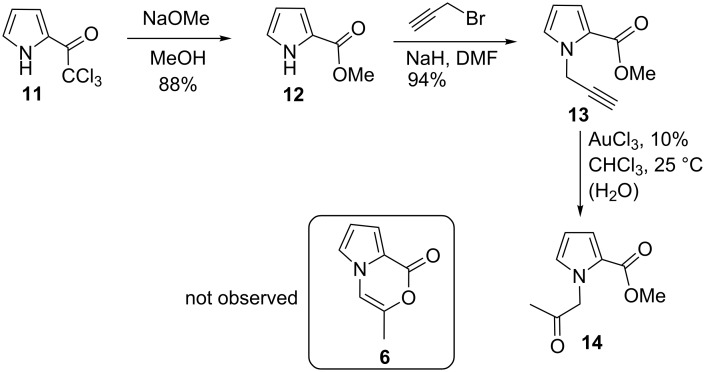
Synthesis of **13** and its reaction with AuCl_3_.

The starting compound **11** was synthesized via a slightly modified route by acetylation of pyrrole with trichloroacetyl chloride in 99% yield [42–43]. The propargyl ester **13** [44–46] was obtained in high yield from the reaction of **12** with propargyl bromide in the presence of NaH as a base (Scheme 2). Gold catalysis is an excellent method for constructing complex chemical architectures in a mild manner that would be difficult to achieve using other reactions [41,47–53]. Reaction of propargyl ester **13** with AuCl_3_ resulted in the formation of **14**, which is an H_2_O (present in the reaction media) addition product to alkyne units. It has been proposed that a gold-activated water intermediate can easily be added to alkynes. The addition of water to alkyne has been reported by several research groups [54–55]. The expected cyclization product having the structure **6** or **7** was not formed.

**Scheme 3:**
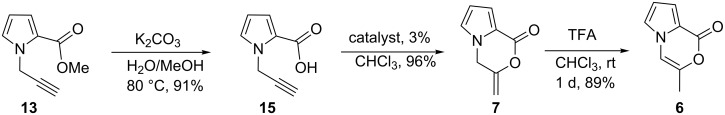
Synthesis of **6**.

After failure of the cyclization reaction of **13** with AuCl_3_, even in water-free solvents, we turned our attention to the construction of the pyrrolo-oxazin-1-one skeleton with the corresponding acid **15**. For this purpose, the propargyl ester **13** was first hydrolyzed with K_2_CO_3_ to acid **15** (Scheme 3) [56]. The acid **15** was reacted with various metal catalysts in chloroform at different temperatures and for different reaction times. Five different catalysts were tried (Table 1). Surprisingly, no reaction was observed when the reaction was conducted with the *N*-heterocyclic carbene (NHC) complex of Au(I) in chloroform (Table 1, entry 5). Reactions with InCl_3_ and PtCl_2_(PPh_3_)_3_ as catalysts gave very poor yields of *exo-*dig cyclization product **7** after 24 h (entries 3 and 4). AgOTf and AuCl_3_ were also screened and AuCl_3_ was identified as the optimal choice due to the shorter reaction time, high yield, and easy isolation of the product **7** (entries 1 and 2, Table 1). We next investigated the isomerization of the double bond in **7**, which was treated with trifluoroacetic acid (TFA) in chloroform at room temperature to give 3-methyl-1*H*-pyrrolo[2,1-*c*][1,4]oxazin-1-one (**6**) [27].

**Table 1:** Optimization of cyclization reaction of **15**.

Entry	Catalyst	Time (h)	Temperature (°C)	Yield (%)

1	AuCl_3_	2	rt	96
2	AgOTf	20	rt	95
3	InCl_3_	24	50	13
4	PtCl_2_(PPh)_3_	26	50	5
5	Au(L)^a^	24	rt	No reaction

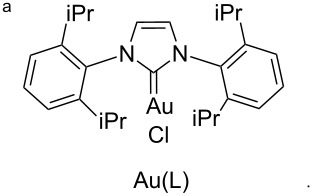

After having obtained the optimal conditions for the Au-catalyzed cyclization of carboxylic acid **15**, we attempted to determine the scope and limitation of this transformation. Then we investigated the cyclization reaction of a range of substituted *N*-propargyl-pyrrole-2-carboxylic acids **17**, **21**, **25**, **29** and **33** and *N*-propargylindole-2-carboxylic acids **37**, **41** and **45**, which were synthesized by hydrolysis of the corresponding esters (Table 2). The Sonogashira cross-coupling reaction [56–61] was used for the synthesis of the desired starting materials **29** and **33**. For the Sonogashira coupling reaction we used a palladium catalyst and a copper(I) cocatalyst in the presence of triphenylphosphine and triethylamine as the base. The carboxylic acids were then submitted to a gold-catalyzed cyclization reaction (Table 2).

**Table 2:** AuCl_3_-catalyzed cyclization reaction of carboxylic acids.

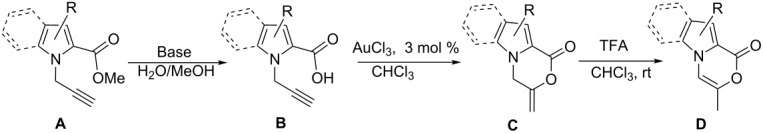			

Esters	Carboxylic acids	Cyclization products	Isomerization products

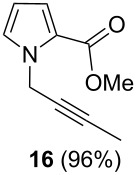	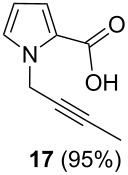	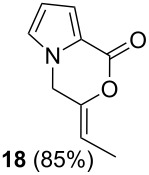	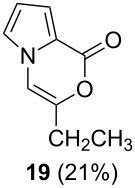
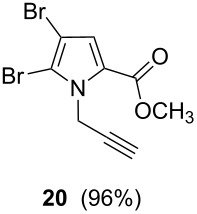	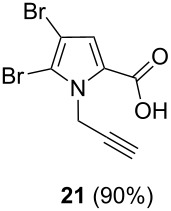	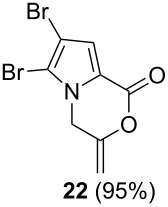	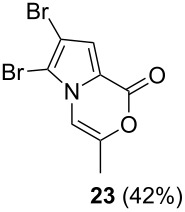
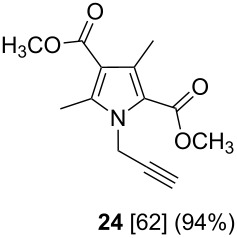	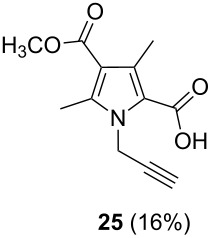	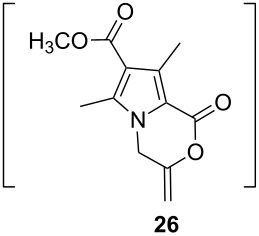	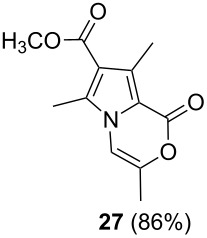
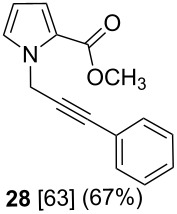	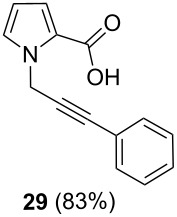	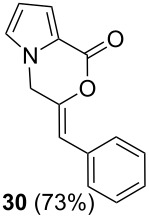	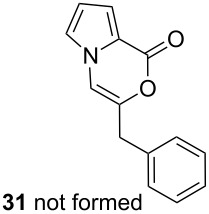
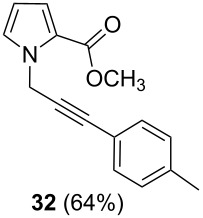	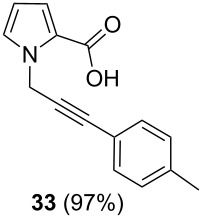	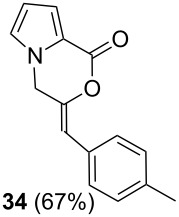	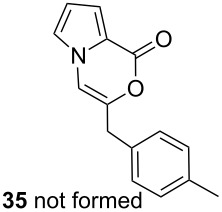
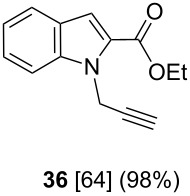	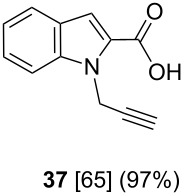	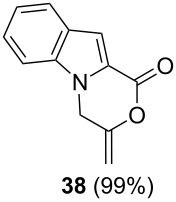	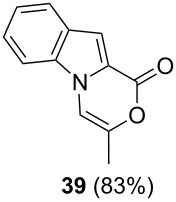
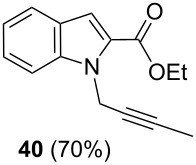	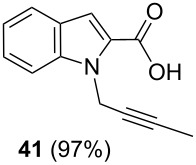	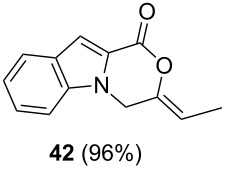	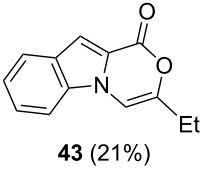
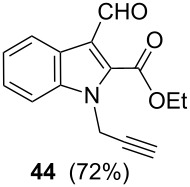	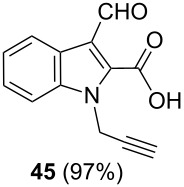	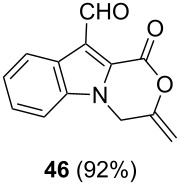	Decomposition

The optimized reaction conditions for cyclization was employed for pyrrole- and indole-carboxylic acids. We observed that all substituted pyrrole as well as indole carboxylic acids underwent a smooth gold-catalyzed cyclization reaction to afford the corresponding oxazinones **18**, **22**, **26**, **30**, **34**, **38**, **42** and **46** in high yields (Table 2). Substitution at the terminal carbon atom of the propargyl group with methyl and phenyl groups did not affect the mode of the cyclization reaction.

Further investigation of cyclic products involved examining the isomerization of the exocyclic double bond. For this purpose, the cyclization products were reacted with trifloroacetic acid (TFA) to form *endo*-cyclic double bonds. Some of the compounds underwent double bond shifts to form an oxazinone skeleton **19**, **23**, **27**, **39** and **43**, whereas the compounds **30** and **34** did not form the corresponding *endo*-cyclic systems **31** and **35**. In order to address this question, we carried out some DFT calculations [66].

**Figure 3:**
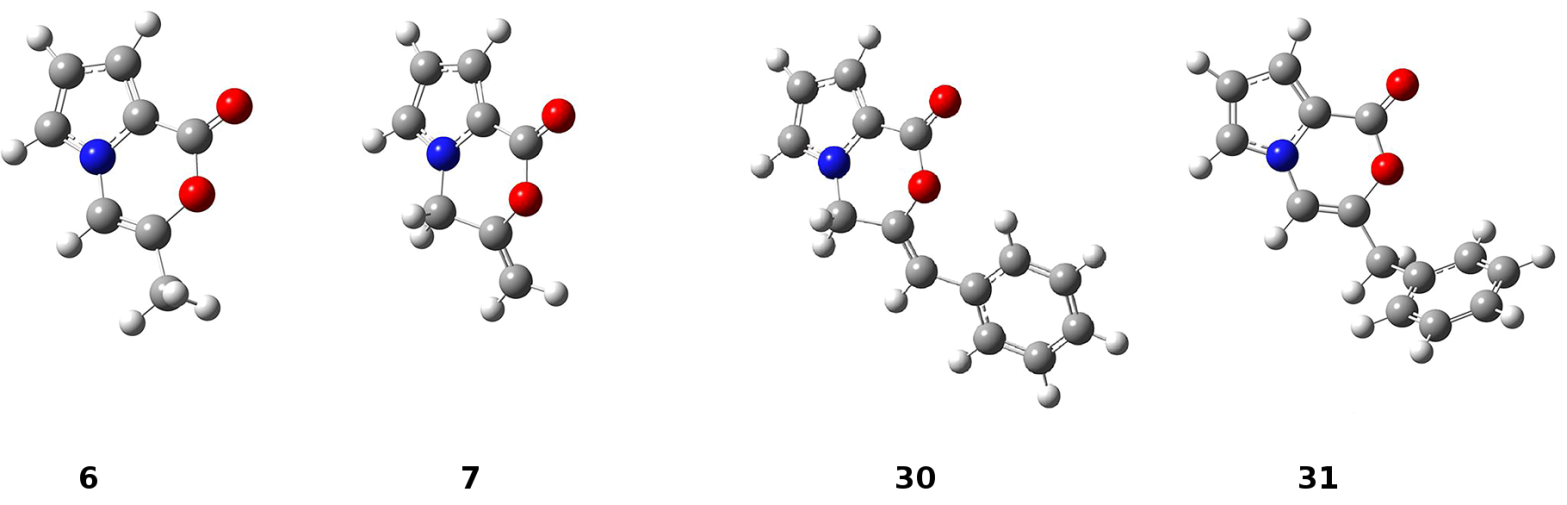
Geometry optimized structures of **6**, **7**, **30** and **31**.

The calculations performed in the gase phase showed that **6** is about 10.6 kcal/mol (DFT, B3LYP at 6-31G** level) and 8.1 kcal/mol (HF/6-311G** level) more stable than **7** (Figure 3). However, in the case of **30** and **31**, we also found that the *endo*-cyclic isomer **31** is thermodynamically about 4.8 kcal/mol (DFT, B3LYP at 6-31G** level) and 3.8 kcal/mol (HF/6-311G** level) more stable than the *exo-*cyclic isomer **30**. Although **31** is thermodynamically more stable than **30**, the resistance of **30** to isomerization in the presence of TFA can be explained by the regioselective protonation of the double bond. The proton is added to the carbon atom to generate the most stable carbocation. The most stable carbocation is the benzylic carbocation which would not undergo a rearrangement.

**Scheme 4:**
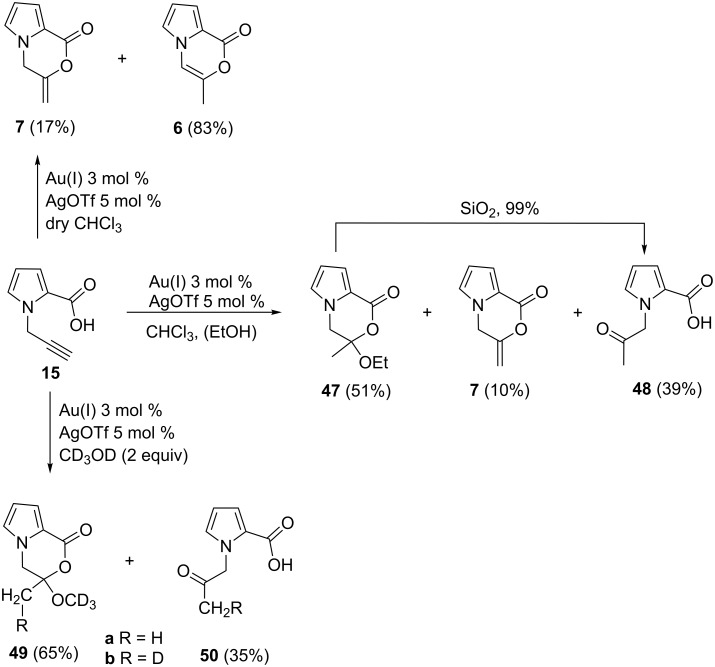
Reaction of **15** with Au(I)/AgOTf in the presence of EtOH and CD_3_OD.

As discussed above, when the cyclization reaction of **15** was carried out with [(NHC)AuCl] complexes (Table 1, entry 5), the starting material was fully recovered. Gold *N*-heterocyclic carbene complexes, in conjunction with a silver salt, were found to efficiently catalyze different types of reactions [67–69]. Therefore, **15** was reacted with [(NHC)AuCl] complex in the presence of AgOTf as the cocatalyst in chloroform. Beside the expected cyclization product **7** (10%), the product **47** was formed conclusively through incorporation of ethanol present in chloroform (<1%) as the major product in 51% yield (Scheme 4). NMR spectral analysis of the mixture revealed also the formation of **48** in 39% yield, which is the hydrolysis product of **47**. Attempts to isolate **47** failed. Chromatography on silica gel converted **47** to **48** in almost quantitative yield. However, the products **6** and **7** only were isolated when the reaction was carried out in EtOH-free chloroform in 83% and 17% yields, respectively (Scheme 4).

To reveal the mechanism for the formation of the products we carried out two different reactions. First, **15** was reacted with CD_3_OD under the same reaction conditions. The ^1^H NMR spectral analysis indicated the formation of **49** and **50** in 65% and 35% yields, respectively. Furthermore, the amount of deuterium atoms in **49** and **50** attached to the methylene group as –CH_2_D was about 64% (Scheme 4).

**Scheme 5:**
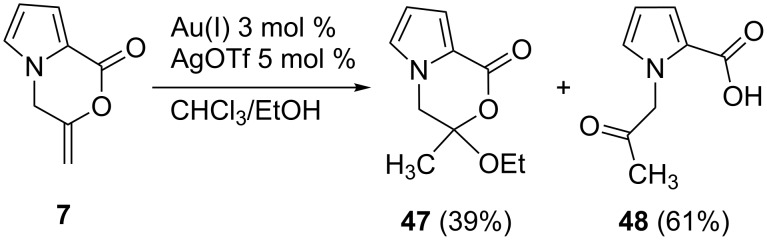
Reaction of **7** with Au(I)/AgOTf in the presence of EtOH.

To prove whether **7** was involved as the intermediate, the cyclization product **7** was reacted with Au(I)/AgOTf with EtOH under the same reaction conditions. As a result, the products **47** and **48** were formed in yields of 39% and 61%, respectively (Scheme 5).

**Scheme 6:**
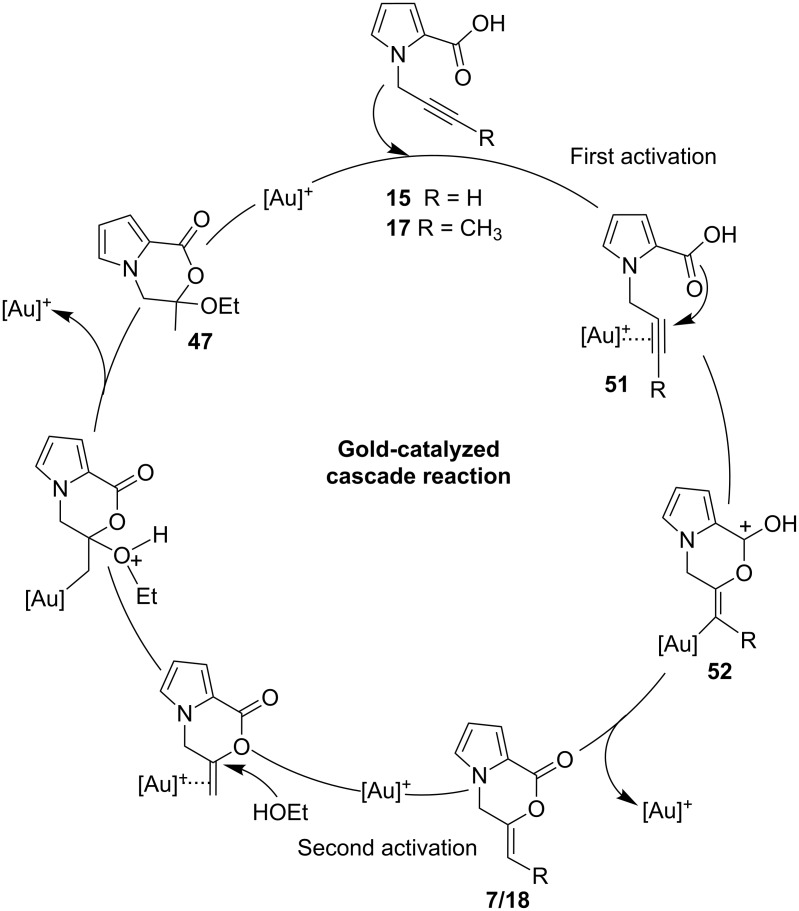
Proposed reaction mechanism for the intramolecular gold-catalyzed cyclization followed by EtOH addition.

Based on all this information obtained, we propose the following gold-catalyzed cascade reaction mechanism. The proposed catalytic cycle was initiated with π-activation of the triple bond by the carbene-based cationic gold species to form the intermediate **51**, which triggers a gold-promoted intramolecular addition of a carboxy group to the alkyne functionality to give methylene-oxazin-1-one derivative **7** (or **18**) (Scheme 6). In the next step, the double bond is activated by π-coordination of the gold(I) catalyst. This enables a nucleophilic attack by the alcohol oxygen, which affords hemiacetal **47**. In case of internal alkynes, two isomeric *E-* and *Z-*oxazinones during the cyclization reaction can be formed. However, we observed exclusive formation of a single isomer. Recently, Michelet et al. [70] and others [41,71–72] demonstrated selectively the formation of *Z-*lactones. The anti intramolecular addition of the carboxylic acid to the gold-alkyne intermediate **51**, forms an intermediate **52** where the substituent is in the *endo*-position. Removal of gold results in the formation of *Z-*isomer. Therefore, we assign the *endo-*configuration to the formed oxazinones.

## Conclusion

We have developed a general synthetic methodology of pyrrolo- and indolo-oxazin-1-one derivatives. The key step was a gold-catalyzed cyclization reaction of N-propargyl-substituted pyrrole and indole carboxylic acid derivatives. The hydroxy groups of carboxylic acids attacked the activated triple bond to form 6-*exo*-*dig* cyclization products, oxazin-1-one derivatives. Some of the *exo-*cyclic double bonds underwent isomerization to endo-cyclic compounds upon treatment with TFA, while some did not. DFT studies supported our findings. Moreover, cyclization reactions in the presence of alcohol formed hemiacetal derivatives after gold-catalyzed cascade reactions.

## Supporting Information

File 1 Experimental and analytical data.

File 2 NMR spectra.
