# Supplementary material for: Gold-catalyzed formation of pyrrolo- and indolo-oxazin-1-one derivatives: The key structure of some marine natural products
Source: Beilstein J Org Chem. 2015 May 28;11:897–905. doi: 10.3762/bjoc.11.101 (PMC4464457; doi:10.3762/bjoc.11.101)
Supplement: File 1 — Experimental and analytical data. [file Beilstein_J_Org_Chem-11-897-s001.pdf]

Supporting Information
for
Gold-catalyzed formation of pyrrolo- and indolo-oxazin-1-one derivatives: The key structure of some marine natural products

Sultan Taskaya,[1] Nurettin Menges,[1,2,*] and Metin Balci[1,*]

Address: [1]Middle East Technical University, Department of Chemistry, Ankara, Turkey, 06800 and [2]Yüzüncü Yil University, Faculty of Pharmacy, Van, Turkey, 65100

Email: Nurettin Menges - nurettinmenges@gmail.com; Metin Balci - mbalci@metu.edu.tr

*Corresponding author

Experimental and analytical data

Contents

1. **General Methods** S4

2. **Experimental**.......... S5

   2.1. Synthesis of **11** S5

   2.2. Synthesis of **12** S5

   2.3. General procedure for propargylation of pyrrole and indole derivatives. S5

   2.4. Synthesis of **13** S6

   2.5. General procedure for hydrolysis of esters to carboxylic acids. S6

   2.6. Synthesis of **15** S6

   2.7. General Procedure for gold-catalyzed cyclization of carboxylic acids S7

   2.8. Synthesis of **7** S7

   2.9. General procedure for TFA-catalyzed isomerization of methylene compounds S7

   2.10. Synthesis of **6** S8

   2.11. Synthesis of **16** S8

   2.12. Synthesis of **17** S8

   2.13. Synthesis of **18** S9

   2.14. Synthesis of **19** S9

   2.15. Synthesis of **20** S10

   2.16. Synthesis of **21** S10

   2.17. Synthesis of **22** S10

   2.18. Synthesis of **23** S11

   2.19. Synthesis of **24** S12

   2.20. Synthesis of **25** S12

   2.21. Synthesis of **27** S13

   2.22. Synthesis of **28** S13

   2.23. Synthesis of **29** S14

   2.24. Synthesis of **30** S14

   2.25. Synthesis of **32** S15

   2.26. Synthesis of **33** S15

   2.27. Synthesis of **34** S16

   2.28. Synthesis of **36** S16

   2.29. Synthesis of **37**. S17

   2.30. Synthesis of **38** S17

   2.31. Synthesis of **39** S17

   2.32. Synthesis of **40** S18

   2.33. Synthesis of **41** S18

2.34. Synthesis of **42** S19
2.35. Synthesis of **43** S19
2.36. Synthesis of **44** S20
2.37. Synthesis of **45** S20
2.38. Synthesis of **46** S21
2.39. Reaction of **15** with gold(I) in chloroform in the presence of ethanol. Formation of **48** S21
2.40. Reaction of **15** with gold(I) in chloroform S22
2.41. Reaction of **15** with gold(I) in the presence of CD_3OD........ S22
2.42. Reaction of **7** with gold(I) in the presence of EtOH........ S23
References........S23
3. Theoretical Methods........ S25
3.1. Theoretical matrix for compound 6........ S25
3.2. Theoretical Matrix for compound 7 .... S25
3.3. Theoretical Matrix for compound 30 .... S26
3.4. Theoretical Matrix for compound 31 .... S27

1. General Methods

All reagents were used as purchased from commercial suppliers without further purification. Proton nuclear magnetic resonance spectra (^{1}H NMR) were recorded on an instrument 400 MHz and chemical shifts are reported in parts per million (ppm) downfield from TMS, using residual $CDCl_3$ as an internal standard. The ^{13}C-NMR spectra were recorded on an instrument 100 MHz and are reported in ppm using solvent as an internal standard ($CDCl_3$. Column chromatography was performed on silica gel (60-mesh). TLC was carried out on 0.2 mm silica gel 60 F254 analytical aluminum plates. High resolution Mass spectra were recorded by LC-MS TOF electrospray ionization technique. Chemicals and all solvents were commercially available and used without further purification. Infrared (IR) spectra were recorded in the range 4000-600 cm-1 via ATR diamond. Melting points were measured using melting point apparatus and were uncorrected. Evaporation of solvents was performed at reduced pressure, using a rotary vacuum evaporator.

2. Experimental

2.1. Synthesis of 2,2,2-trichloro-1-(1*H*-pyrrol-2-yl)ethanone (11) [1,2]. To a stirred solution of 2,2,2-trichloroacetyl chloride (25.5 mL, 0.227 mol) in dry ether (45 mL) was added pyrrole (14.3 mL, 0.206 mmol) at 0 ºC dropwise in an ice bath and the mixture was stirred for 2 d. After completion of the reaction, brine solution (300 mL) was added to the solution. The mixture was extracted with ethyl acetate (3 × 100 mL) and water (100 mL). The combined organic extracts were dried over $MgSO_4$ and the solvent was evaporated to give 2,2,2-trichloro-1-(1*H*-pyrrol-2-yl)ethanone as brown solid (43.6 g, 99%). **^{1}H-NMR** (400 MHz, $CDCl_3$) δ 9.60 (bs, 1H, NH), 7.45 – 7.35 (m, 1H), 7.21 – 7.12 (m, 1H), 6.45 – 6.30 (m, 1H). **^{13}C NMR** (100 MHz, $CDCl_3$) δ 173.2, 127.2, 123.0, 121.2, 111.9, 94.9.

2.2. Synthesis of methyl 1*H*-pyrrole-2-carboxylate (12) [1,2]. To a stirred solution of **11** (31.87 g, 0.150 mol) in methanol (50 mL) was added a solution of NaOMe solution in methanol (50 mL), prepared by dissolving of Na (3.46 g, 0.150 mol) in methanol, dropwise at 0 °C for 3 h. The solvent was evaporated and diluted HCl (50 mL) was added to residue. The mixture was extracted with ethyl acetate (3 × 150 mL) and dried over $MgSO_4$. Evaporation of solvent gave the ester **12** as a brown solid (16.5 g, 88%). **^{1}H NMR** (400 MHz, $CDCl_3$) δ 9.73 (s, 1H, NH), 6.98–6.94 (m, 1H), 6.94–6.91 (m, 1H), 6.27–6.23 (m, 1H), 3.85 (s, 3H, OMe). **^{13}C NMR** (100 MHz, $CDCl_3$) δ 162.0, 123.3, 122.5, 115.4, 110.4, 51.5.

2.3. General procedure for propargylation of pyrrole and indole derivatives. To a stirred solution of substituted methyl 1*H*-pyrrole-2-carboxylate (5 mmol) in DMF (10 mL) was added solid NaH (8 mmol) portionwise at 0 °C over 30 min. The reaction mixture was stirred at room temperature for 30 min. followed by dropwise addition of propargyl bromide or 1-bromobut-2-yne (6 mmol) in DMF (5 mL). The resulting mixture was stirred at room temperature for 2 d. Water (30 mL) was added, and the mixture was extracted with

EtOAc (3 × 40 mL). The combined organic extracts were washed with brine (4 × 25 mL) and dried over $MgSO_4$. Evaporation of solvent gave propargyl esters.

2.4. Synthesis of methyl 1-prop-2-ynyl-1*H*-pyrrole-2-carboxylate (13) [3-5]. To a stirred solution of methyl 1*H*-pyrrole-2-carboxylate (**12**) (12.02 g, 96.16 mmol) in DMF (30 mL) was added solid NaH (3.46 g, 144.25 mmol) portionwise at 0 °C over 30 min. followed by dropwise addition of (80%, 10.67 mL, 120.2 mmol) in DMF (10 mL). After work-up as described above, propargyl ester **13** was isolated as pale yellow viscous liquid (14.82, 94%). **^{1}H NMR** (400 MHz, $CDCl_3$) δ 7.04 (dd, $J_{5,4}$ = 2.7 and $J_{5,3}$ = 1.8 Hz, 1H, H-5), 6.89 (dd, $J_{3,4}$ = 4.0 and $J_{3,5}$ = 1.8 Hz, 1H, H-3), 6.09 (dd, $J_{4,3}$ = 4.0 Hz, $J_{4,5}$ = 2.7 Hz, 1H, H-4), 5.09 (d, ^4J = 2.6 Hz, 2H, CH_2), 3.73 (s, 3H, $-OCH_3$), 2.35 (t, ^4J = 2.6 Hz, 1H, C≡CH). **^{13}C NMR** (100 MHz, $CDCl_3$) δ 161.5, 127.9, 121.6, 118.5, 108.6, 78.3, 73.8, 51.1, 38.1.

2.5. General procedure for hydrolysis of esters to carboxylic acids. To a solution of propargyl esters (5 mmol) in methanol (5 mL) was added K_2CO_3 (12 mmol) in $MeOH/H_2O$ (20 mL) mixture and the solution was heated at reflux temperature for 1 d. Then, a solution of diluted HCl was added until the reaction media was acidic. The mixture was extracted with EtOAc (3 × 80 mL) and then with water (30 mL). The combined organic extracts were dried over $MgSO_4$ and the solvent was evaporated to give the corresponding acids.

2.6. Synthesis of 1-prop-2-ynyl-1*H*-pyrrole-2-carboxylic acid (15) [6]. A solution of **13** (2.02 g, 12.4 mmol) in methanol (5 mL) was reacted with K_2CO_3 (3.757 g, 27.24 mmol) in $MeOH/H_2O$ (40 mL) as described above. **15** was isolated as a white solid (1.67 g, 91%). **^{1}H NMR** (400 MHz, $CDCl_3$) δ 7.14 (dd, $J_{5,4}$ = 2.7 Hz, $J_{5,3}$ = 1.8 Hz, 1H, H-5), 7.08 (dd, $J_{3,4}$ = 4.0 Hz, $J_{3,5}$ = 1.8 Hz, 1H, H-3), 6.16 (dd, $J_{4,3}$ = 4.0 Hz, $J_{4,5}$ = 2.7 Hz, 1H, H-4), 5.11 (d, J= 2.6 Hz, 2H, $-CH_2$), 2.39 (t, ^4J = 2.6 Hz, 1H, C≡CH). **^{13}C NMR** (100 MHz, $CDCl_3$) δ 165.9, 129.2, 120.9, 120.8, 109.1, 77.9, 74.1, 38.4.

2.7. General Procedure for gold-catalyzed cyclization of carboxylic acids. To a stirred solution of carboxylic acid (1 mmol) in chloroform (5 mL) was added 3 mol % $AuCl_3$ at room temperature and the reaction mixture was stirred for 2 h. After completion of the reaction, controlled by TLC, the solvent was evaporated to give the cyclization products, which was crystallized from appropriate solvent.

2.8. Synthesis of 3-methylene-3,4-dihydro-1*H*-pyrrolo[2,1-*c*][1,4]oxazin-1-one (7). A stirred solution of **15** (75 mg, 0.5 mmol) in chloroform (5 mL) was reacted with 3 mol % $AuCl_3$ (4 mg) as described above to give the cyclization product **7**. The crude product was crystallized from chloroform under the hexane atmosphere to get analytically pure sample. Colorless needles (72 mg, 96%) from $CHCl_3$/n-hexane, m.p. 65-67 °C. ^{1}**H NMR** (400 MHz, $CDCl_3$) δ 7.13 (dd, $J_{8,7}$ = 4.0 and $J_{8,6}$ = 1.5 Hz, 1H, H-8), 6.89 (dd, $J_{6,7}$ = 2.5 and $J_{6,8}$ = 1.5 Hz, 1H, H-6), 6.34 (dd, $J_{7,8}$ = 4.0 and $J_{7,6}$ = 2.5 Hz, 1H, H-7), 5.02 (bd, $^2J_{gem}$ = 2.2 Hz, 1H, H-1), 4.76 (bs, 2H, CH_2), 4.70 (dt, ^2J = 2.2 Hz, $^4J_{1,4}$ = 1.1 Hz, 1H, H-1′). ^{13}**C NMR** (100 MHz, $CDCl_3$) δ 155.2, 148.6, 124.9, 118.0 (2C), 111.7, 98.3, 45.5 **IR** (ATR) 3114, 2994, 1721, 1663, 1532, 1485, 1399, 1329, 1245, 1206, 1175, 1064, 882, 732. **HRMS** calcd for ($C_8H_7NO_2$) $[M + H]^+$: 150.0550; Found: 150.0556.

2.9. General procedure for TFA-catalyzed isomerization of methylene compounds. To a stirred solution of cyclization product (2 mmol) in chloroform (5 mL) was added excess trifloroacetic acid (15 mmol) at room temperature and the reaction was stirred for 1 d. After completion of the reaction, which was controlled by TLC, the solvent was evaporated under the reduced pressure. Water (50 mL) was added and the mixture was extracted with ethyl acetate (3 × 30 mL). The combined organic extracts were washed with brine and dried over $MgSO_4$. Removal of the solvent gave the corresponding 1*H*-pyrrolo[2,1-*c*][1,4]oxazin-1-one derivatives.

2.10. Synthesis of 3-methyl-1*H*-pyrrolo[2,1-*c*][1,4]oxazin-1-one (6) A stirred solution of 3-methylene-3,4-dihydro-1*H*-pyrrolo[2,1-*c*][1,4]oxazin-1-one (**7**) (0.27 g, 1.8 mmol) in chloroform (5 mL) was treated with excess trifloroacetic acid (15 mmol) at room temperature as described above to give **6**. Colorless needles from chloroform/n-hexane, m.p. 95-97 °C (lit. m.p. 92-94 °C[9a]). **^{1}H NMR** (400 MHz, $CDCl_3$) δ 7.11 (bd, $J_{8,CH3}$ =4.1 Hz, 1H, H-8), 6.97 (dd, $J_{6,7}$ = 2.5, $J_{6,8}$ = 1.5 Hz, 1H, H-6), 6.75–6.71 (m, 1H, H-4), 6.43 (dd, $J_{7,8}$ = 4.1, $J_{7,6}$ = 2.5 Hz, 1H, H-7), 2.08 (d, J = 1.1 Hz, 3H, CH_3). **^{13}C NMR** (100 MHz, $CDCl_3$) δ 155.4, 141.0, 120.7, 116.5, 115.1, 112.8, 104.9, 16.7.

2.11. Synthesis of methyl 1-but-2-ynyl-1*H*-pyrrole-2-carboxylate (16). A stirred solution of methyl 1*H*-pyrrole-2-carboxylate (**12**) (0.300 g, 2.4 mmol) in DMF (5 mL), NaH (92 mg, 3.8 mmol), and 1-bromobut-2-yne (0.275 ml, 0.414 g, 3.1 mmol) in DMF (2 mL) were reacted as described above to give **16**. Yellow viscous liquid (410 mg, 96%). **^{1}H NMR** (400 MHz, $CDCl_3$) δ 7.17–7.13 (m, 1H, H-5), 6.96 (dd, $J_{3,4}$ = 3.9 and $J_{3,5}$ = 1.8 Hz, 1H, H-3), 6.16 (dd, $J_{4,3}$ = 3.9 and $J_{4,5}$ = 2.7 Hz, 1H, H-4), 5.11 (q, $^5J_{1,4}$ = 2.4 Hz, 2H, CH_2), 3.81 (s, 3H, OMe), 1.85 (t, $^5J_{4,1}$ = 2.4 Hz, 3H, -CH_3). **^{13}C NMR** (100 MHz, $CDCl_3$) δ 161.5, 127.9, 121.5, 118.4, 108.2, 81.7, 73.5, 51.1, 38.6, 3.6. **IR** (ATR) 2921, 2852, 1699, 1531, 1437, 1410, 1345, 1237, 1196, 1104, 739. **HRMS** calcd for ($C_{10}H_{11}NO_2$) $[M + H]^+$: 178.0863; found: 178.0858.

2.12. Synthesis of 1-but-2-ynyl-1*H*-pyrrole-2-carboxylic acid (17). A solution of methyl 1-but-2-ynyl-1*H*-pyrrole-2-carboxylate (**16**) (0.354 g, 2 mmol) in methanol (3 mL) was hydrolyzed with K_2CO_3 (0.607 g, 4.4 mmol) in MeOH/H_2O (8 mL) mixture as described above to give **17**. Pale yellow plates (310 mg, 95%), m.p. 133-135 °C from methanol. **^{1}H NMR** (400 MHz, CD_3 OD) δ 7.17 – 7.14 (m, 1H, H-5), 6.94 (dd, $J_{3,4}$ = 3.9 and $J_{3,5}$ = 1.8 Hz, 1H, H-3), 6.14 (dd, $J_{4,3}$ = 3.9 and $J_{4,5}$ = 2.7 Hz, 1H, H-4), 5.11 (q, $^5J_{1,4}$ = 2.4 Hz, 2H, CH_2), 1.83 (t, $^5J_{4,1}$ = 2.4 Hz, 3H, CH_3). **^{13}C NMR** (100 MHz, CD_3OD) δ 164.1, 129.5,

123.0, 119.9, 109.1, 81.9, 75.0, 39.2, 3.1. **IR** (ATR) 3352, 2921, 2617, 1663, 1532, 1436, 1320, 1259, 1109, 1073, 885, 741, 727, 612. **HRMS** calcd for ($C_9H_9NO_2$) $[M - H]^+$: 162.0561; found: 162.0591.

2.13. Synthesis of (3*Z*)-3-Ethylidene-3,4-dihydro-1*H*-pyrrolo[2,1-*c*][1,4]oxazin-1-one (18). A stirred solution of 1-but-2-ynyl-1***H***-pyrrole-2-carboxylic acid (**17**) (100 mg, 0.61 mmol) in chloroform (4 mL) was reacted with 3 mol % $AuCl_3$ (5.5 mg) at room temperature as described above to give **18**. The compound was purified by silica gel column chromatography eluting with dichloromethane. Viscous oil (85 mg, 85%). **^{1}H NMR** (400 MHz, $CDCl_3$) δ 7.12 (dd, $J_{3,4}$ = 4.0 and $J_{3,5}$ = 1.4 Hz, 1H, H-3), 6.86 (dd, $J_{5,4}$ = 2.5 and $J_{5,3}$ = 1.4 Hz, 1H, H-5), 6.31 (dd, $J_{4,3}$ = 4.0 and $J_{4,5}$ = 2.5 Hz, 1H, H-4), 5.08 (qt, $J_{H,CH3}$ = 6.9 and $^4J_{H,CH2}$ = 1.1 Hz, 1H, C=CH), 4.66 − 4.68 (m, CH_2), 1.78 (dd, $J_{CH3,H}$ = 6.9 Hz, $^5J_{CH3,CH2}$= 1.1 Hz, 3H, -CH_3). **^{13}C NMR** (100 MHz, $CDCl_3$) δ 155.8, 141.7, 124.5, 118.6, 117.7, 111.4, 109.3, 46.2, 9.8. **IR** (ATR) 1732, 1694, 1532, 1483, 1397, 1338, 1306, 1169, 1095, 1054, 964, 883, 735. **HRMS** calcd for ($C_9H_9NO_2$) $[M + H]^+$: 164.0706; found: 164.0689.

2.14. Synthesis of 3-ethyl-1*H*-pyrrolo[2,1-c][1,4]oxazin-1-one (19). A stirred solution of (3*Z*)-3-ethylidene-3,4-dihydro-1*H*-pyrrolo[2,1-c][1,4]oxazin-1-one (**18**) (70 mg, 0.43 mmol) in chloroform (5 mL) was reacted with trifloroacetic acid as described above to give the isomerized product **19**. The compound was purified by silica gel column chromatography eluting with n-hexane/EtOAc (3:1). Colorless viscous liquid (15 mg, 21%), crude yield 27%. **^{1}H NMR** (400 MHz, $CDCl_3$) δ 7.21 (bd, $J_{8,7}$ = 4.0, 1H, H-8), 7.07 (dd, $J_{6,7}$ = 2.3 and $J_{6,8}$ = 1.4 Hz, 1H, H-6), 6.82 (bs, 1H, H-4), 6.53 (dd, $J_{7,8}$ = 4.0 Hz, $J_{7,6}$ = 2.3 Hz, 1H, H-7), 2.48 (q, J = 7.3 Hz, 2H, CH_2), 1.25 (t, J = 7.3 Hz, 3H, CH_3). **^{13}C NMR** (100 MHz, $CDCl_3$) δ 155.6, 146.0, 120.8, 116.7, 115.1, 112.8, 104.0, 24.2, 11.1. **IR** (ATR)

3121, 2970, 2922, 1717, 1691, 1531, 1485, 1458, 1378, 1344, 1213, 1090, 1072, 1036, 1014, 936, 728, 630. **HRMS** calcd for ($C_9H_9NO_2$) $[M + H]^+$: 164.0706; found: 164.0716.

2.15. Synthesis of methyl 4,5-dibromo-1-prop-2-ynyl-1*H*-pyrrole-2-carboxylate (20). A stirred solution of methyl 4,5-dibromo-1*H*-pyrrole-2-carboxylate [7,8] (0.283 g, 1 mmol) in DMF (5 mL) was reacted with NaH (0.036 g, 1.5 mmol) and propargyl bromide (80%, 0.110 ml, 1.25 mmol) as described above to give the propargyl ester **20**. Pale yellow needles from diethyl ether, (0.308 g, 96%), m. p. 82-84 °C. **^{1}H NMR** (400 MHz, $CDCl_3$) δ 7.04 (s, 1H, H-3), 5.30 (d, J = 2.5 Hz, 2H, CH_2), 3.85 (s, 3H, OCH_3), 2.32 (t, J = 2.5 Hz, 1H, C≡CH). **^{13}C NMR** (100 MHz, $CDCl_3$) δ 159.9, 123.2, 120.2, 113.1, 100.0, 77.5, 72.8, 51.7, 37.7. **IR** (ATR) 2922, 1697, 1508, 1433, 1391, 1320, 1253, 1204, 1114, 1089, 937, 821, 747.

2.16. Synthesis of 4,5-dibromo-1-prop-2-ynyl-1*H*-pyrrole-2-carboxylic acid (21). Methyl 4,5-dibromo-1-(prop-2-yn-1-yl)-1*H*-pyrrole-2-carboxylate (**20**) (0.294 g, 0.916 mmol) in methanol (3 mL) was hydrolyzed with K_2CO_3 (0.278 g, 2 mmol) in MeOH/H_2O (14 mL) as described above to give the corresponding acid **21**. White powder (0.254 g, 90%) from diethyl ether, m.p. 186-189 °C. ^{1}H NMR (400 MHz, CD_3OD) δ 7.05 (s, H-3), 5.36 (d, J = 2.4 Hz, 2H, CH_2), 2.75 (t, J = 2.4 Hz, 1H, C≡CH). **^{13}C NMR** (100 MHz, CD_3OD) δ 162.1, 125.3, 121.1, 113.7, 100.5, 78.9, 74.0, 38.4. **IR** (ATR) 3280, 2847, 1669, 1525, 1420, 1319, 1258, 1215, 1134, 980, 936, 890, 756, 697. **HRMS** calcd for ($C_8H_5Br_2NO_2$) $[M - H]^-$: 303.8614; found: 303.8650.

2.17. Synthesis of 6,7-dibromo-3-methylene-3,4-dihydro-1*H*-pyrrolo[2,1-*c*][1,4]oxazin-1-one (22). A stirred solution of 4,5-dibromo-1-(prop-2-yn-1-yl)-1*H*-pyrrole-2-carboxylic acid (**21**) (0.123 mg, 0.4 mmol) in methanol (5 mL) was reacted with 3 mol % $AuCl_3$ (3.6 mg) as described above to give the cyclization product **22**. Yellow needles (0.117 g, 95%) from $CHCl_3$/n-hexane, m.p. 149-152 °C. **^{1}H NMR** (400 MHz, $CDCl_3$) δ 7.11 (s, 1H, H-8),

5.03 (bd, ${}^{2}J_{1,1'}$ = 2.5 Hz, 1H, H-1), 4.75 (dt, ${}^{2}J_{1,1'}$ = 2.5 Hz, ${}^{4}J_{1,4}$ = 1.1 Hz, 1H, H-1′), 4.62 (bs, 2H, H-4). ^{13}C NMR (100 MHz, $CDCl_3$) δ 153.4, 147.2, 119.7, 119.5, 109.6, 102.3, 100.1, 45.4. **IR** (ATR) 3134, 1726, 1670, 1536, 1460, 1388, 1340, 1303, 1261, 1193, 1128, 1075, 992, 895, 731. **HRMS** calcd for $[M - H]^-$: 303.8614; found: 303.8647.

2.18. Synthesis of 6,7-dibromo-3-methyl-1*H*-pyrrolo[2,1-*c*][1,4]oxazin-1-one (23). A stirred solution of 6,7-dibromo-3-methylene-3,4-dihydro-1*H*-pyrrolo[2,1-***c***][1,4]oxazin-1-one (**22**) (70 mg, 0.43 mmol) in chloroform (5 mL) was reacted with trifloroacetic acid as described above to give the isomerized product **23.** The compound was purified by silica gel column chromatography eluting with n-hexane/EtOAc (8:1). Pale yellow needles (25 mg, 42%), crude yield 54% from chloroform, m.p. 187-190 °C. **^{1}H NMR** (400 MHz, $CDCl_3$) δ 7.26 (s, 1H), 6.89 (bs, 1H), 2.20 (d, J = 1.0 Hz, 3H, CH_3). **^{13}C NMR** (100 MHz, $CDCl_3$) δ 153.3, 142.6, 117.7, 116.8, 105.0, 104.1, 103.2, 17.1. **IR** (ATR) 3119, 1732, 1685, 1440, 1391, 1378, 1350, 1298, 1220, 1179, 1128, 1065, 992, 818, 773, 731, 629, 545. **HRMS** calcd for ($C_8H_5Br_2NO_2$) $[M - H]^-$: 303.8614; found: 303.8639.

Synthesis of Dimethyl 3,5-dimethyl-1H-pyrrole-2,4-dicarboxylate [9]. To a mixture of methylacetoacetate (17.2 ml, 0.16 mol) and acetic acid (40 ml) was added an aqueous solution of $NaNO_2$ (5.52 g, 0.08 mol) in water (10 mL) over 30 min at 0 °C. The yellowish reaction mixture was stirred for 2.5 h at 10 °C. Then, Zn powder (10.46 g, 0.16 mol) was added to this solution portionwise at room temperature and the resulting mixture was then heated to 50 °C for 10 min. to allow excess amount of Zn powder to react completely. Heating was then continued at 95 °C for 1 h. After cooling to room temperature, the yellowish precipitate was collected and washed with ice water (300 mL) to give dimethyl 3,5-dimethyl-1*H*-pyrrole-2,4-dicarboxylate. Pale yellow powder (7.6 g, 45%). **^{1}H-NMR** (400 MHz, $CDCl_3$) δ 9.26 (bs, 1H, NH), 3.79 (s, 3H, OMe), 3.75 (s, 3H, OMe), 2.48 (s,

3H, CH_3), 2.44 (s, 3H, CH_3). **^{13}C-NMR** (100 MHz, $CDCl_3$) δ 165.9, 162.3, 159.2, 139.4, 131.1, 117.8, 113.4, 51.3, 50.7, 14.2, 12.0.

2.19. Synthesis of dimethyl 3,5-dimethyl-1-prop-2-ynyl-1*H*-pyrrole-2,4-dicarboxylate (24) [10]. A stirred solution of dimethyl 3,5-dimethyl-1*H*-pyrrole-2,4-dicarboxylate (3.011 g, 14.26 mmol) in DMF (27 mL), solid NaH (0.683 g, 28.45 mmol), and propargyl bromide (80%, 2.23 mL, 24.95 mmol) were reacted as described above to give dimethyl 3,5-dimethyl-1-(prop-2-yn-1-yl)-1*H*-pyrrole-2, 4-dicarboxylate (**24**). Pale yellow cubic crystals (3.336 g, 94%) from EtOAc, m. p. 98-100 °C. **^{1}H NMR** (400 MHz, $CDCl_3$) δ 5.09 (d, $J_{1,3}$ = 2.5 Hz, 2H, CH_2), 3.80 (s, 3H, OCH_3), 3.75 (s, 3H, OCH_3), 2.54 (s, 3H, CH_3), 2.46 (s, 3H, CH_3), 2.21 (t, $J_{3,1}$ = 2.5 Hz, 1H, H-3). **^{13}C NMR** (100 MHz, $CDCl_3$) δ 165.8, 162.4, 141.5, 131.9, 119.2, 113.3, 78.3, 72.2, 51.1, 50.8, 34.8, 12.7, 11.8. **IR** (ATR) 3283, 2956, 2364, 1688, 1540, 1432, 1287, 1248, 1217, 1180, 1136, 1102, 770, 628. **HRMS** calcd for $[M + H]^+$: 250.1074; found: 250.1058.

2.20. Synthesis of 4-(methoxycarbonyl)-3,5-dimethyl-1-prop-2-ynyl-1*H*-pyrrole-2-carboxylic acid (25). A solution of dimethyl 3,5-dimethyl-1-(prop-2-yn-1-yl)-1*H*-pyrrole-2,4-dicarboxylate (**24**) (1.226 g, 4.92 mmol) in methanol was reacted with K_2CO_3 (1.5 g, 10.82 mmol) in MeOH/H_2O (30 mL) as described above. The mono acid **25** was separated by column chromatography on silica gel eluting with hexane/EtOAc (1:1) to yield 4-(methoxycarbonyl)-3,5-dimethyl-1-(prop-2-yn-1-yl)-1*H*-pyrrole-2-carboxylic acid (**25**). White powder (0.185 g, 16%) from $CHCl_3$/n-hexane, m.p. 165-167 ºC. **^{1}H NMR** (400 MHz, CD_3COCD_3) δ 5.34 (d, $J_{1,3}$ = 2.5 Hz, 2H, CH_2), 3.80 (s, 3H, OCH_3), 2.85 (t, $J_{3,1}$ = 2.5 Hz, 1H, H-3), 2.64 (s, 3H, CH_3), 2.56 (s, 3H, CH_3). **^{13}C NMR** (100 MHz, CD_3COCD_3) δ 166.0, 163.1, 142.3, 132.1, 120.1, 113.9, 79.7, 73.7, 50.9, 35.1, 12.9, 11.8. **IR** (ATR) 3272, 2620, 1699, 1645, 1540, 1486, 1432, 1373, 1262, 1219, 1148, 1112, 920, 783, 705, 661. **HRMS** calcd for $[M - H]^-$: 234.0772; found: 234.0782.

2.21. Synthesis of methyl 3,6,8-trimethyl-1-oxo-1*H*-pyrrolo[2,1-*c*][1,4]oxazine-7-carboxylate (27). A stirred solution of 4-(methoxycarbonyl)-3,5-dimethyl-1-prop-2-ynyl-1*H*-pyrrole-2-carboxylic acid (**25)** (70 mg, 0.3 mmol) in chloroform (4 mL) was reacted with 3 mol % $AuCl_3$ (2.7 mg) as described above above. The analysis of the mixture with ^{1}H-NMR indicated the presence of two cyclization products **26** and **27**. Then, the crude product was reacted with TFA in $CHCl_3$ at room temperature to give methyl 3,6,8-trimethyl-1-oxo-1*H*-pyrrolo[2,1-***c***][1,4]oxazine-7-carboxylate (**27**). Pale yellow cubical crystals (60 mg, 86%) from chloroform/n-hexane, m.p. 182-184 °C. ^{1}**H NMR** (400 MHz, $CDCl_3$) δ 6.56-6.58 (m, 1H), 3.79 (s, 3H, $-OCH_3$), 2.62 (s, 3H, CH_3), 2.50 (s, 3H, CH_3), 2.07 (d, J = 1.0 Hz, 3H, CH_3). ^{13}**C NMR** (100 MHz, $CDCl_3$) δ 165.3, 155.3, 141.8, 133.3, 132.4, 115.4, 112.7, 101.3, 51.1, 17.1, 12.1, 11.0. **IR** (ATR) 1726, 1684, 1557, 1406, 1252, 1191, 1130, 1027, 983, 953, 786, 746. **HRMS** calcd for ($C_{12}H_{13}NO_4$) $[M + H]^+$: 236.0917; found: 236.0905.

2.22. Synthesis of methyl 1-(3-phenylprop-2-ynyl)-1*H*-pyrrole-2-carboxylate (28) [11]. To a solution of methyl 1-prop-2-ynyl-1*H*-pyrrole-2-carboxylate (**13**) (0.5 g, 3.06 mmol) in dry THF (7 mL) and dry diisopropyl amine (3 mL, 0.021 mmol) was added iodobenzene (0.625 g, 3.06 mmol), palladium acetate (9 mg, 0.04 mmol), cuprous iodide (4.6 mg, 0.024 mmol) and triphenyl phosphine (15 mg, 0.057 mmol). The mixture was heated at reflux temperature for 24 h. After evaporation of the solvent, H_2O (50 mL) was added to the residue and extracted with EtOAc (3 × 50 mL). The combined organic extracts were dried over $MgSO_4$. Removal of the solvent under the reduced pressure gave the crude product (0.650 g). The residue was purified by silica gel column chromatography eluting with 5:1 hexane/EtOAc to afford methyl 1-(3-phenylprop-2-ynyl)-1*H*-pyrrole-2-carboxylate (**28**) (0.494 g, 67%) as a yellow liquid. ^{1}**H NMR** (400 MHz, $CDCl_3$) δ 7.38 – 7.34 (m, 2H), 7.25–7.21 (m, 3H), 7.16 (dd, $J_{5,4}$ = 2.7 and $J_{5,3}$ = 1.8 Hz, 1H, H-5), 6.92 (dd, $J_{3,4}$ = 4.0 and

$J_{3,5}$ = 1.8 Hz, 1H, H-3), 6.11 (dd, $J_{4,3}$ = 4.0 and $J_{4,5}$ = 2.7 Hz, 1H, H-4), 5.32 (s, 2H, CH_2), 3.75 (s, 3H, OCH_3).**^{13}C NMR** (100 MHz, $CDCl_3$) δ 161.6, 131.8, 128.6, 128.3, 128.0, 122.4, 121.7, 118.5, 108.5, 85.6, 83.5, 51.2, 39.0.

2.23. Synthesis of 1-(3-phenylprop-2-ynyl)-1*H*-pyrrole-2-carboxylic acid (29). A solution of methyl 1-(3-phenylprop-2-ynyl)-1*H*-pyrrole-2-carboxylate (**28**) (0.238 g, 1 mmol) in methanol (3 mL) was reacted with 2 N KOH in MeOH (4 mL) and H_2O (0.5 mL) mixture as described above to give **29**. Yellow cubical crystals (0.187 g, 83%) from chloroform, m.p. 146-148 °C. **^{1}H NMR** (400 MHz, $CDCl_3$) δ 7.40 – 7.36 (m, 2H), 7.27 – 7.22 (m, 4H, 3 arom. 1 pyrrole), 7.09 (dd, $J_{3,4}$ = 4.0 and $J_{3,5}$ = 1.8 Hz, 1H, H-3), 6.17 (dd, $J_{4,3}$ = 4.0 and $J_{4,5}$ = 2.7 Hz, 1H, H-4), 5.32 (s, 2H, CH_2). **^{13}C NMR** (100 MHz, $CDCl_3$) δ 166.1, 131.8, 129.2, 128.7, 128.3, 122.3, 122.1, 120.8, 108.9, 85.9, 83.2, 39.3. **IR** (ATR) 2868, 2624, 1663, 1534, 1441, 1326, 1266, 1114, 1075, 907, 752, 729, 689. **HRMS** Calcd for ($C_{14}H_{11}NO_2$) $[M - H]^-$: 224.0717; Found: 224.0724.

2.24. Synthesis of (3*Z*)-3-Benzylidene-3,4-dihydro-1*H*-pyrrolo[2,1-*c*][1,4]oxazin-1-one (30). A stirred solution of 1-(3-phenylprop-2-ynyl)-1*H*-pyrrole-2-carboxylic acid (**29**) (100 mg, 0.44 mmol) in chloroform (5 mL) was reacted with 3 mol % $AuCl_3$ (4 mg) as described above to give the cyclization product **30**. Pale yellow plates (73 mg, 73%) from chloroform/n-hexane, m.p. 86-88 °C. **^{1}H NMR** (400 MHz, $CDCl_3$) δ 7.63 (d, J = 8.1 Hz, 2H), 7.29 (bt, J = 7.8 Hz, 2H), 7.19 (tt, J = 7.4 and J = 1.0 Hz, 1H), 7.08 (dd, J = 4.0 and J = 1.5 Hz, 1H), 6.84 (dd, J = 2.5 and J = 1.5 Hz, 1H), 6.26 (dd, J = 4.0 and J= 2.5 Hz, 1H), 5.76 (s, 1H, H-1), 4.75 (s, 2H, H-4). **^{13}C NMR** (100 MHz, $CDCl_3$) δ 154.9, 141.1, 132.7, 129.5, 128.6, 128.0, 124.8, 118.2, 118.0, 112.8, 111.7, 46.7. **IR** (ATR) 2920, 1718, 1684, 1532, 1483, 1395, 1308, 1252, 1163, 1064, 1017, 936, 857, 756, 729, 693. **HRMS** calcd for ($C_{14}H_{11}NO_2$) $[M + H]^+$: 226.0863; found: 226.0852.

2.25. Synthesis of methyl 1-[3-(4-methylphenyl)prop-2-ynyl]-1*H*-pyrrole-2-carboxylate (32). Cuprous iodide (4.6 mg, 0.024 mmol), triphenyl phosphine (15.0 mg, 0.057 mmol), palladium acetate (9.0 mg, 0.01 mmol) and dry diisopropyl amine (3 mL, 0.021 mmol) was added to a solution of methyl 1-prop-2-ynyl-1*H*-pyrrole-2-carboxylate (**13**) (0.5 g, 3.06 mmol) in dry THF (7 mL). Then, 4-iodotoluene (0.673 g, 3.06 mmol) was added to the reaction mixture at room temperature. The mixture was heated at reflux temperature during stirring for 24 h. After cooling to room temperature, solvent was removed under reduced pressure. H_2O (50 mL) was added to the residue and extracted with ethyl acetate (3 × 50 mL) and lastly the combined organic layers were washed with brine (80 mL) dried over $MgSO_4$ and removal of the solvent under the reduced pressure gave the crude product (0.695 g). Chromatography on a silica gel column eluting with hexane/EtOAc (5:1) afforded the coupling product **32**. Yellow solid (0.496 g, 64%) from EtOAc, m.p. 47-49 °C. **^{1}H NMR** (400 MHz, $CDCl_3$) δ 7.26 (bd, J = 8.1 Hz, 2H), 7.18 (dd, $J_{5,4}$ = 2.7 and $J_{5,3}$ = 1.8 Hz, 1H, H-5), 7.04 (bd, J = 8.1 Hz, 2H), 6.92 (dd, $J_{3,4}$ = 3.9 and $J_{3,5}$ = 1.8 Hz, 1H, H-3), 6.11 (dd, $J_{4,3}$ = 3.9 and $J_{4,5}$ = 2.7 Hz, 1H, H-4), 5.31 (s, 2H, CH_2), 3.76 (s, 3H, OCH_3), 2.27 (s, 3H, CH_3). **^{13}C NMR** (100 MHz, $CDCl_3$) δ 161.6, 138.8, 131.7, 129.1, 128.0, 121.7, 119.3, 118.5, 108.4, 85.8, 82.7, 51.2, 39.1, 21.5. **IR** (ATR) 2945, 1705, 1509, 1477, 1437, 14010, 1340, 1291, 1229, 1101, 1070, 808, 756, 733, 604. **HRMS** calcd for ($C_{16}H_{15}NO_2$) [M + H]: 254.1176; found: 254.1187.

2.26. Synthesis of 1-[3-(4-methylphenyl)prop-2-ynyl]-1*H*-pyrrole-2-carboxylic acid (33). A solution of methyl 1-[3-(4-methylphenyl)prop-2-ynyl]-1*H*-pyrrole-2-carboxylate (**32**) (0.120 g, 0.5 mmol) in methanol and KOH (2 N) in MeOH (7 mL) were reacted as described above to give 1-[3-(4-methylphenyl)prop-2-ynyl]-1H-pyrrole-2-carboxylic acid (**33**) Yellow needles (0.110 g, 97%) from chloroform, m.p. 139-142 °C. **^{1}H NMR** (400 MHz, $CDCl_3$) δ 7.27 (bd, J = 8.0 Hz, 2H), 7.25 (dd, $J_{5,4}$ = 2.7 and $J_{5,3}$ = 1.6 Hz, 1H, H-5),

7.09 (dd, $J_{3,4}$ = 3.9 and $J_{3,5}$ = 1.6 Hz, 1H, H-3), 7.05 (bd, J = 8.0 Hz, 2H), 6.16 (dd, $J_{4,3}$ = 3.8 and $J_{4,5}$ = 2.7 Hz, 1H, H-4), 5.31 (s, 2H, CH_2), 2.27 (s, 3H, CH_3). **^{13}C NMR** (100 MHz, $CDCl_3$) δ 166.2, 138.9, 131.7, 129.3, 129.1, 121.0, 120.8, 119.2, 108.9, 86.1, 82.5, 39.3, 21.5. **IR** (ATR) 3200, 2924, 1725, 1667, 1430, 1332, 1251, 1106, 1070, 811, 760, 732, 579. **HRMS** calcd for ($C_{15}H_{13}NO_2$) $[M - H]^-$: 238.0873; found: 238.0891.

2.27. Synthesis of (3*Z*)-3-(4-methylbenzylidene)-3,4-dihydro-1*H*-pyrrolo[2,1-c][1,4]oxazin-1-one (34). A stirred solution of 1-[3-(4-methylphenyl)prop-2-ynyl]-1*H*-pyrrole-2-carboxylic acid (**33**) (104 mg, 0.43 mmol) in chloroform (5 mL) was reacted with 3 mol % $AuCl_3$ (4.0 mg) as described above. The crude product (94 mg) was purified on a silica gel column eluting with n-hexane/EtOAc (5:1) to afford the cyclization product **34**. Pale yellow pellets (70 mg, 67%) from chloroform/n-hexane, m.p. 175-178 °C. **^{1}H NMR** (400 MHz, $CDCl_3$) δ 7.61 (bd, J = 8.1 Hz, 2H), 7.20–7.13 (m, 3H), 6.91 (dd, J = 2.5 and 1.3 Hz,1H), 6.34 (dd, J = 4.0 and J = 2.5 Hz, 1H), 5.81 (s, 1H), 4.83 (s, 2H, CH_2), 2.35 (s, 3H, CH_3). **^{13}C NMR** (100 MHz, $CDCl_3$) δ 155.0, 140.4, 138.0, 129.9, 129.4, 129.3, 124.7, 118.1, 112.8, 111.6, 46.9, 21.3. **IR** (ATR) 2960, 2920, 2849, 2718, 1734, 1466, 1395, 1328, 1152, 751, 731. **HRMS** calcd for ($C_{15}H_{13}NO_2$) [M + H]: 240.1019; found: 240.0991.

2.28. Synthesis of ethyl 1-prop-2-ynyl-1*H*-indole-2-carboxylate (36) [12,13]. A stirred solution of ethyl 1*H*-indole-2-carboxylate (0.7 g, 3.7 mmol) in DMF (6 mL), NaH (0.142 g, 5.92 mmol), and propargyl bromide (80%, 0.431 ml, 5 mmol) in DMF (3 mL) were reacted as described above to give propargyl indole derivative **36**. White powder (0.830 g, 98%) from petroleum ether m.p. 64-66 °C. **^{1}H NMR** (400 MHz, $CDCl_3$) δ 7.61 (bd, J = 8.0 Hz, 1H), 7.42 (dd, J = 8.4 and J = 0.7 Hz, 1H), 7.31 (ddd, J = 8.3, 7.0, and 1.1 Hz, 1H), 7.26 (d, J = 0.7 Hz, 1H), 7.11 (ddd, J = 8.0, 7.0, and 0.8 Hz, 1H), 5.36 (d, $J_{1,3}$ = 2.5 Hz, 2H, CH_2), 4.32 (q, J = 7.1 Hz, 2H, CH_2), 2.17 (t, J= 2.5 Hz, 1H, C≡CH), 1.33 (t, J = 7.1

Hz, 3H, CH_3). **^{13}C NMR** (100 MHz, $CDCl_3$) δ 162.0, 139.0, 126.9, 126.3, 125.5, 122.8, 121.2, 111.4, 110.5, 78.8, 72.0, 60.8, 33.9, 14.3.

2.29. Synthesis of 1-prop-2-ynyl-1*H*-indole-2-carboxylic acid (37). A solution of ethyl 1-prop-2-ynyl-1*H*-indole-2-carboxylate (**36**) (0.475 g, 2.1 mmol) in methanol (5 mL) was reacted with K_2CO_3 (0.636 g, 4.6 mmol) in MeOH/H_2O (10 mL) mixture as described above to give indole carboxylic acid **37**. Colorless needles (0.405 g, 2.0 mmol, 97%) from chloroform, m.p. 194.0-198 °C (Lit. m.p. 190-193 °C). **^{1}H NMR** (400 MHz, $CDCl_3$) δ 7.66 (bd, J = 7.9 Hz, 1H), 7.46 (d, J = 8.0 Hz, 1H), 7.45 (bs, 1H), 7.38 (t, J = 7.7 Hz, 1H), 7.17 (t, J = 7.4 Hz, 1H), 5.39 (d, J = 2.1 Hz, 2H, CH_2), 2.20 (t, J = 2.1 Hz, 1H, C≡CH). **^{13}C NMR** (100 MHz, CD_3OD) δ 164.8, 140.5, 128.5, 127.7, 126.3, 123.6, 122.0, 112.6, 111.8, 80.1, 73.0, 34.4.

2.30. Synthesis of 3-methylene-3,4-dihydro-1*H*-[1,4]oxazino[4,3-*a*]indol-1-one (38). A stirred solution of 1-(prop-2-yn-1-yl)-1*H*-indole-2-carboxylic acid (**37**) (0.199 g, 1 mmol) in methanol (5 mL) was reacted with 3 mol % $AuCl_3$ (9.1 mg) as described above to afford the cyclization product **38**. Pale yellow needles (197 mg, 99%) from chloroform/n-hexane, m.p. 201-204 °C. **^{1}H NMR** (400 MHz, $CDCl_3$) δ 7.67 (bd, J = 8.0 Hz, 1H), 7.41 (d, J = 0.8 Hz, 1H), 7.35 (ddd, J = 8.4, 7.0, and 1.0 Hz , 1H), 7.27 (dd, J = 8.4 and 0.8 Hz, 1H), 7.15 (ddd, J = 8.0, 7.0, and 1.0 Hz, 1H), 5.02 (bd, ^{2}J = 2.3 Hz, 1H, C=CH), 4.81 (bs, 2H, CH_2), 4.74 (dt, ^{2}J = 2.3 and ^{4}J = 1.1 Hz, 1H, C=CH). **^{13}C NMR** (100.6 MHz, $CDCl_3$) δ 156.2, 148.7, 136.6, 127.2, 126.4, 123.4, 122.1, 121.8, 110.8, 110.0, 98.8, 42.5. **IR** (ATR) 1729, 1664, 1534, 1476, 1459, 1352, 1313, 1243, 1164, 1136, 1079, 998, 869, 729. **HRMS** calcd for ($C_{12}H_9NO_2$) $[M + H]^+$: 200.0706; found: 200.0708.

2.31. Synthesis of 3-Methyl-1H-[1,4]oxazino[4,3-a]indol-1-one (39). A stirred solution of 3-methylene-3,4-dihydro-1*H*-indolo[2,1-*c*][1,4]oxazin-1-one (**38**) (0.127 g, 0.63 mmol) in chloroform (5 mL) was reacted with trifloroacetic acid as described above to give the

isomerized product **39**. Yellow plates (106 mg, 83%) from chloroform/n-hexane, m.p. 205-206 °C. **^{1}H NMR** (400 MHz, $CDCl_3$) δ 7.80 (d, J = 8.1 Hz, 1H), 7.55 (d, J = 8.5 Hz, 1H), 7.52 (s, 1H), 7.45 (ddd, J = 8.3, 7.0, and 1.0 Hz, 1H), 7.32 – 7.27 (m, 1H), 7.09 (bs, 1H), 2.25 (d, J = 1.1 Hz, 3H, CH_3). **^{13}C NMR** (100 MHz, $CDCl_3$) δ 156.6, 138.8, 132.8, 127.4, 125.9, 123.2, 122.6, 120.3, 110.4, 107.7, 102.4, 16.9. **IR** (ATR) 1732, 1559, 1539, 1458, 1406, 1351, 1328, 1248, 1166, 1135, 1069, 996, 809, 728. **HRMS** calcd for $[M + H]^+$: 200.0706; found: 200.0697.

2.32. Synthesis of ethyl 1-but-2-ynyl-1*H*-indole-2-carboxylate (40). A stirred solution of ethyl 1*H*-indole-2-carboxylate (0.270 g, 1.4 mmol) in DMF (3 mL) NaH (52 mg, 2.1 mmol), and 1-bromobut-2-yne (0.126 ml, 0.190 g, 1.4 mmol) in DMF (2 mL) were reacted as described above to give the propargyl indole derivative **40**. White crystals like snowflake (0.240 g, 70%) from petroleum ether, m.p. 66-68 °C. **^{1}H NMR** (400 MHz, $CDCl_3$) δ 7.71 (bd, J = 8.0 Hz, 1H), 7.54 (dd, J = 8.4 and J = 0.7 Hz, 1H), 7.41 (ddd, J = 8.2, 7.0, and 1.0 Hz, 1H), 7.35 (d, J = 0.7 Hz, 1H), 7.00 (ddd, J = 7.9, 7.1, and 0.8 Hz, 1H), 5.39 (q, $^5J_{1,4}$ = 2.4 Hz, 2H, CH_2), 4.40 (q, J = 7.1 Hz, 2H, CH_2), 1.75 (t, $^5J_{4,1}$ = 2.4 Hz, 3H, CH_3), 1.42 (t, J = 7.1 Hz, 3H, CH_3). **^{13}C NMR** (100 MHz, $CDCl_3$) δ 162.0, 139.0, 127.0, 126.2, 125.5, 122.7, 120.9, 111.0, 110.8, 79.7, 74.2, 60.7, 34.2, 14.4, 3.6. **IR** (ATR) 3058, 2973, 1698, 1519, 1473, 1453, 1340, 1317, 1262, 1249, 1195, 1144, 1094, 1029, 822, 766, 736. **HRMS** calcd for ($C_{15}H_{15}NO_2$) $[M + H]^+$: 242.1176; found: 242.1174.

2.33. Synthesis of 1-but-2-ynyl-1*H*-indole-2-carboxylic acid (41) A solution of ethyl 1-but-2-ynyl-1*H*-indole-2-carboxylate (**40**) (0.307 g, 1.27 mmol) in methanol (5 mL) was reacted with K_2CO_3 (0.387 g, 2.8 mmol) in $MeOH/H_2O$ (10 mL as described above to give indolecarboxylic acid **41**. White needle (0.405 g, 97%) from chloroform, m.p. 190-193 °C. **^{1}H NMR** (400 MHz, $CDCl_3$) δ 7.64 (d, J = 8.0 Hz, 1H), 7.46 (d, J = 8.5 Hz, 1H), 7.42 (s, 1H), 7.34 (ddd, J = 8.0, 7.0, and 0.9 Hz, 1H), 7.15–7.10 (m, 1H), 5.31 (q, $^5J_{1,4}$ = 2.3 Hz,

2H, CH_2), 1.68 (t, $^5J_{4,1}$ = 2.3 Hz, 3H, CH_3). **^{13}C NMR** (100 MHz, $CDCl_3$) δ 166.9, 139.6, 126.1, 126.0, 125.7, 123.0, 121.2, 113.5, 111.0, 80.0, 74.0, 34.3, 3.6 **IR** (ATR) 2851, 2513, 1654, 1518, 1482, 1438, 1264, 1206, 1142, 829, 733, 618 **HRMS** calcd for ($C_{13}H_{11}NO_2$) $[M - H]^-$: 212.0717; found: 212.0767.

2.34. Synthesis of (3*Z*)-3-ethylidene-3,4-dihydro-1*H*-[1,4]oxazino[4,3-*a*]indol-1-one (42). A stirred solution of 1-but-2-ynyl-1***H***-indole-2-carboxylic acid (**41**) (80 mg, 0.37 mmol) in chloroform (4 mL) was reacted with 3 mol % $AuCl_3$ (3.4 mg) as described above to give the cyclization product **42**. Yellow plates (77 mg, 96%) from chloroform/n-hexane, m.p. 102-105 °C. **^{1}H NMR** (400 MHz, $CDCl_3$) δ 7.72 (dd, *J* = 8.1, 0.7 Hz, 1H), 7.44 (s, 1H), 7.42–7.37 (m, 1H), 7.32 (d, *J* = 8.4 Hz, 1H), 7.19 (dd, *J* = 8.0 and 7.0 Hz, 1H), 5.18 (q, *J* = 6.8 Hz, 1H, C=CH), 4.77 (d, *J* = 1.0 Hz, 2H, -CH_2-), 1.81 (dd, *J* = 6.8 and *J* = 1.0 Hz, 3H, CH_3). **^{13}C NMR** (100 MHz, $CDCl_3$) δ 156.8, 141.8, 136.4, 127.1, 126.2, 123.3, 122.7, 121.6, 110.4, 110.0, 109.7, 43.1, 9.9. **IR** (ATR) 2862, 1727, 1697, 1530, 1466, 1415, 1374, 1297, 1242, 1215, 1157, 1094, 1060, 812, 724. **HRMS** calcd for ($C_{13}H_{11}NO_2$) $[M + H]^+$: 214.0863; found: 214.0864.

2.35. Synthesis of 3-ethyl-1*H*-[1,4]oxazino[4,3-*a*]indol-1-one (43). A stirred solution of (3*Z*)-3-ethylidene-3,4-dihydro-1*H*-[1,4]oxazino[4,3-*a*]indol-1-one (**42**) (120 mg, 0.56 mmol) in chloroform (5 mL) was reacted with trifloroacetic acid as described above to give the isomerized product **43**. Pale yellow solid (25 mg, 21%) from EtOAc (crude yield 37%), m.p. 96-98 °C. **^{1}H NMR** (400 MHz, $CDCl_3$) δ 7.84 (bd, *J* = 8.0 Hz, 1H), 7.60 (bd, *J* = 8.3 Hz, 1H), 7.55 (s, 1H), 7.48 (ddd, *J* = 8.3, 7.0, 0.9 Hz, 1H), 7.32 (ddd, *J* = 8.0, 7.0, 0.9 Hz, 1H), 7.11 (bs, 1H), 2.59 (dd, *J* = 1,0 and 7.5 Hz, 2H, CH_2), 1.33 (t, *J* = 7.5 Hz, 3H, CH_3). **^{13}C NMR** (100 MHz, $CDCl_3$) δ 156.8, 143.9, 132.9, 127.5, 125.9, 123.3, 122.6, 110.4, 107.7, 101.5, 100.0, 24.4, 11.4 **IR** (ATR) 2918, 1724, 1691, 1536, 1459, 1408,

1351, 1245, 1169, 1134, 1039, 1013, 806, 728. **HRMS** calcd for ($C_{13}H_{11}NO_2$) $[M + H]^+$: 214.0863; found: 214.0873.

2.36. Synthesis of ethyl 3-formyl-1-prop-2-ynyl-1*H*-indole-2-carboxylate (44). A stirred solution of ethyl 3-formyl-1*H*-indole-2-carboxylate [12] (0.600 g, 2.76 mmol) in DMF (6 mL), NaH (0.1 g, 4.14) and propargyl bromide (80%, 0.31 ml, 3.6 mmol) in DMF (3 mL) were reacted as described above to give propargyl derivative **44**. White needles (0.510 g, 72%) from chloroform, m.p. 119-122 °C. **^{1}H NMR** (400 MHz, $CDCl_3$) δ 10.66 (s, 1H), 8.52 (d, J = 8.1 Hz, 1H), 7.55 (bd, J = 8.4 Hz, 1H), 7.49 (ddd, J = 8.0, 6.8, and 1.0 Hz, 1H), 7.39 (ddd, J = 8.0, 7.0, and 1.0 Hz, 1H), 5.41 (d, J = 2.5 Hz, 2H, CH_2), 4.55 (q, J = 7.1 Hz, 2H, CH_2), 2.33 (t, J = 2.4 Hz, 1H), 1.49 (t, J = 7.1 Hz, 3H, CH_3).**^{13}C NMR** (100 MHz, $CDCl_3$) δ 188.5, 160.8, 137.4, 132.2, 126.7, 124.7, 124.4, 124.0, 120.8, 110.5, 77.5, 73.2, 62.4, 34.7, 14.2. **IR** (ATR) 3247, 2121, 1698, 1655, 1510, 1474, 1428, 1364, 1270, 1246, 1212, 1169, 1142, 1039, 1013, 814, 785, 705. **HRMS** calcd for ($C_{15}H_{13}NO_3$) $[M+H]^+$: 256.0968; found: 256.0975.

2.37. Synthesis of 3-formyl-1-prop-2-ynyl-1*H*-indole-2-carboxylic acid (45). A solution of ethyl 3-formyl-1-prop-2-ynyl-1*H*-indole-2-carboxylate (**44**) (0.255 g, 1 mmol) in methanol (4 mL) was hydrolized with K_2CO_3 (0.304 g, 2.2 mmol) in MeOH/H_2O (10 mL) mixture as described above to give the carboxylic acid **45**. Yellow powder (0.220 g, 97%) from chloroform, m.p. 190-193 °C. **^{1}H NMR** (400 MHz, CD_3COCD_3) δ 10.53 (s, 1H, CHO), 8.29 (d, J = 8.1 Hz, 1H), 7.65 (d, J = 8.5 Hz, 1H), 7.39 (ddd, J = 8.4, 7.2, and 1.1 Hz, 1H), 7.26 (bt, J = 7.6 Hz, 1H), 5.47 (d, J = 2.5 Hz, 2H, CH_2), 2.77 (t, J = 2.5 Hz, 1H, C≡CH). **^{13}C NMR** (100 MHz, CD_3COCD_3) δ 188.9, 161.8, 138.5, 133.7, 127.3, 125.7, 125.0, 123.7, 121.0, 112.3, 79.0, 74.5, 35.3. **IR** (ATR) 3264, 2375, 2315, 1685, 1559, 1518, 1458, 1373, 1333, 1272, 1252, 1215, 1176, 1040, 898, 810, 744, 679. **HRMS** calcd for ($C_{13}H_9NO_3$) $[M+H]^+$: 228.0655, found: 228.0658.

2.38. Synthesis of 3-methylene-1-oxo-3,4-dihydro-1*H*-[1,4]oxazino[4,3-*a*]indole-10-carbaldehyde (46). A stirred solution of 3-formyl-1-prop-2-ynyl-1*H*-indole-2-carboxylic acid (**45**) (150 mg, 0.66 mmol) in chloroform (5 mL) was reacted with 3 mol % $AuCl_3$ (6.0 mg) as described above to give the cyclization product. Orange plates (138 mg, 92%) from chloroform/n-hexane, m.p. 193-194 °C. **^{1}H NMR** (400 MHz, $CDCl_3$) δ 10.74 (s, 1H, CHO), 8.46 (d, J = 8.5 Hz, 1H), 7.54 – 7.48 (m, 1H), 7.43 – 7.37 (m, 2H), 5.21 (d, J = 2.6 Hz, 1H, C=CH), 4.96 (s, 2H, CH_2), 4.95 – 4.92 (m, 1H, C=CH). **^{13}C NMR** (100 MHz, $CDCl_3$) δ 187.7, 154.6, 147.2, 135.2, 127.6, 125.4, 125.1, 124.9, 124.3, 121.2, 110.0, 100.3, 42.4. **IR** (ATR) 3034, 2848, 1735, 1648, 1535, 1471, 1426, 1310, 1251, 1206, 1158, 1109, 1043, 997, 866, 844, 746. **HRMS** calcd for ($C_{13}H_9NO_3$) $[M + H]^+$: 228.0655; found: 228.0657.

2.39. Reaction of 1-prop-2-ynyl-1*H*-pyrrole-2-carboxylic acid (15) with gold(I) in chloroform in the presence of ethanol. Formation of 1-(2-oxopropyl)-1*H*-pyrrole-2-carboxylic acid (48). To a solution of 1-prop-2-ynyl-1*H*-pyrrole-2-carboxylic acid (**15**) (0.149 g, 1 mmol) in dry $CHCl_3$ (5 mL) was added 3 mol % 1,3-bis (2,6-di-isopropylphenyl)imidazole-2-ylidene gold(I) (18.6 mg), 5 mol % AgOTf (12.8 mg) and EtOH (1 mmol, 58 µL). The solution was stirred at room temperature for 1 d. After the completion of the reaction, controlled by TLC, the solvent was evaporated to give crude product. The ^{1}H NMR spectral analysis of the residue revealed the formation of three products; **47**, **48**, and **7** in yields of 51%, 39%, and 10%, respectively. The ^{1}H NMR spectral data of **47** were extracted from the spectrum of the mixture. The residue was chromatographed on silica gel eluting with n-hexane/EtOAc (5:1) to yield **48** as pale yellow needles (147 mg, 88%) from chloroform, m.p. 140-142 °C. **^{1}H NMR** (400 MHz, $CDCl_3$) δ 7.17 (dd, $J_{3,4}$ = 4.0 and $J_{3,5}$ = 1.7 Hz, 1H, H-3), 6.85 (dd, $J_{5,4}$ = 2.6 and $J_{5,3}$ =1.7 Hz, 1H, H-5), 6.28 (dd, $J_{4,3}$ = 4.0 Hz, $J_{4,5}$ = 2.6 Hz, 1H, H-4), 5.06 (s, 2H, CH_2), 2.22 (s,

3H, CH_3). **^{13}C NMR** (100 MHz, $CDCl_3$) δ 202.4, 166.0, 130.8, 121.2, 120.5, 109.4, 58.4, 26.8. **IR** (ATR) 2917, 2868, 2587, 1731, 1646, 1533, 1470, 1432, 1328, 1274, 1174, 1114, 1081, 927, 747, 605, 581, 547. **HRMS** calcd for ($C_8H_9NO_3$) $[M+Na]^+$: 190.0475, found: 190.0471.

3-Ethoxy-3-methyl-3,4-dihydro-1*H*-pyrrolo[2,1-*c*][1,4]oxazin-1-one (47). **^{1}H NMR** (400 MHz, $CDCl_3$) δ 7.14 (dd, $J_{3,4}$ = 4.0 and $J_{3,5}$ = 1.6 Hz, 1H, H-3), 6.85 (dd, $J_{5,4}$ = 2.6 and $J_{5,3}$ =1.6 Hz, 1H, H-5), 6.29 (dd, $J_{4,3}$ = 4.0 Hz, $J_{4,5}$ = 2.5 Hz, 1H, H-4), 4.17 (d, A-part of AB-system, ^2J = 12.8 Hz, 1H, NCH_2), 4.12 (d, B-part of AB-system, ^2J = 12.8 Hz, 1H, NCH_2), 3.81-3.67 (m, 2H, OCH_2), 1.67 (s, 3H, CH_3), 1.10 (t, J = 7.1 Hz, 3H, CH_3).

2.40. Reaction of 1-prop-2-ynyl-1H-pyrrole-2-carboxylic acid (15) with gold(I) in chloroform. The reaction was carried out with 1 mol carboxylic acid **15** as described above. The ^{1}H NMR spectral analysis of the reaction mixture indicated the sole formation of **6** and **7** in 83% and 17% yields, respectively.

2.41. Reaction of 1-prop-2-ynyl-1*H*-pyrrole-2-carboxylic acid (15) with gold(I) in the presence of CD_3OD. A solution of 1-prop-2-ynyl-1*H*-pyrrole-2-carboxylic acid (**15**) (1 mmol) and CD_3OD in dry $CHCl_3$ (5 mL) was reacted with 3 mol % 1,3-bis(2,6-di-isopropylphenyl)imidazole-2-ylidene gold(I) (18.6 mg), 5 mol % AgOTf (12.8 mg) as described above. The ^{1}H NMR spectral analysis of the residue revealed the formation of three products; **49a/49b**, and **50a/50b** in yields of 65% and 35%, respectively. The ^{1}H NMR spectral data for **49** were extracted from the spectrum of the mixture. Then, the mixture was submitted to silica gel chromatography. The product **50a/50b** was isolated as the sole product in 92%.

3-Methoxy-3-methyl-3,4-dihydro-1*H*-pyrrolo[2,1-*c*][1,4]oxazin-1-one (49a and 49b). **^{1}H NMR** (400 MHz, $CDCl_3$) δ 7.13 (dd, $J_{3,4}$ = 3.9 and $J_{3,5}$ = 1.2 Hz, 1H, H-3), 6.87 (bs, 1H, H-5), 6.32 (dd, $J_{4,3}$ = 3.9 Hz, $J_{4,5}$ = 2.5 Hz, 1H, H-4), 4.20 (d, A-part of AB-system, J =

12.9 Hz, 1H, NCH_2), 4.16 (d, B-part of AB-system, J = 12.9 Hz, 1H, NCH_2), 1.69 (s, 3H, CH_3), 1.68 (t, $^2J_{H,D}$ = 1.6 Hz, 2H, CH_2D). **^{13}C NMR** (100 MHz, $CDCl_3$) δ 157.6, 145.5, 124.4, 117.3, 110.0, 102.7, 51.8 (CH_2), 49.4 (h, $J_{C,D}$ = 21.7 Hz, OCD_3), 20.8 (CH_3), 20.6 (t, $J_{C,D}$ = 19.8 Hz, CH_2D).

1-(2-Oxopropyl)-1*H*-pyrrole-2-carboxylic acid (50a and 50b (Ratio 31/69)). **^{1}H NMR** (400 MHz, $CDCl_3$) δ 7.08 (dd, $J_{3,4}$ = 4.0 and $J_{3,5}$ = 1.7 Hz, 1H, H-3), 6.76 (bdd, $J_{5,4}$ = 2.5 and $J_{5,3}$ = 1.7 Hz, 1H, H-5), 6.18 (dd, $J_{4,3}$ = 4.0 Hz, $J_{4,5}$ = 2.5 Hz, 1H, H-4), 4.96 (s, 2H, NCH_2), 2.12 (s, 3H, CH_3), 2.10 (t, $^2J_{H,D}$ = 2.2 Hz, 2H, CH_2D). **^{13}C NMR** (100 MHz, $CDCl_3$) δ 202.4, 165.9, 130.8, 121.2, 120.5, 109.4, 58.4, 26.8 (t, $J_{C,D}$ = 20.1 Hz).

2.42. Reaction of 3-methylene-3,4-dihydro-1*H*-pyrrolo[2,1-*c*][1,4]oxazin-1-one (7) with gold(I) in the presence of EtOH. A solution **7** (1 mmol) and EtOH in $CHCl_3$ (5 mL) was reacted with 3 mol % 1,3-bis (2,6-di-isopropylphenyl)imidazole-2-ylidene gold(I) 5 mol % AgOTf as described above. The ^{1}H NMR spectral analysis of the residue revealed the formation of two products; **47**, and **48** in yields of 39% and 61%, respectively.

References

1. Hewlett, N. M.; Tepe, J. J. *Org. Lett.* **2011**, *13*, 4550–4553.
2. Bailey, D. M.; Johnson, R. E.; Albertson, N. F. *Org. Synth*. **1971**, *51*, 100–102.
3. Menges, N.; Sari, O.; Abdullayev, Y.; Erdem, S. S.; Balci, M. *J. Org. Chem.* **2013**, *78*, 5184–5195.
4. Nguyen, T. M.; Guzei, I. A. Lee, D. *J. Org. Chem.* **2002**, *47*, 6553–6556.
5. Nguyen, T. M.; Lee, D. *Org. Lett.* **2001**, *3*, 3161–3163.
6. Molteni, G. *Heterocycles* **2004**, *63*, 1423–1428.
7. Forenza, S.; Minale, L.; Riccio, R.; Fattorusso, E. *J. Chem. Soc., Chem. Commun*. **1971**, 1129–1130.

8. Wischang, D. Hartung, J. *Tetrahedron* **2011**, *67*, 4048–4054.

9. Groening, C.; Kemper, R.; Frede, M.; Ebel, K. *Ger. Offen.* **2000**, DE 19858352 A1 20000621.

10. Meng, G., Zheng, M., Wang, M. *Org. Prep. Proced. Int.* **2011**, *43*, 308–311.

11. Fumie, S., PCT Int. Appl. **1996**, 232 pp. WO 9628250 A1 19960919.

12. Verniest, G.; Padwa, A. *Org. Lett.* **2008**, *10*, 4379–4382.

13. Kuuloja, N.; Tois, J.; Franzen, R. *Tetrahedron Asymmetry* **2011**, *22*, 468–475.

3. Theoretical Methods

Geometric optimizations were performed by using Gaussian 09 through Gaussview 5.0.9. and the method of DFT/B3LYP/6-311G**. Absolute values of NMR shielding were calculated by using the Gauge-Independent Atomic Orbital (GIAO) method with the restricted closed shell formalism employing DFT/B3LYP/6-311G** basis set over DFT/B3LYP/6-311G** optimized. NICS values were obtained by calculating absolute NMR shielding at different points of the rings. The normal mode analyses for each structure have yielded no imaginary frequencies for the 3N _ 6 vibrational degrees of freedom, where N is the number of atoms in the system, which indicates that the structure of each molecule corresponds to at least a local minimum on the potential energy surface.

3.1. Theoretical matrix for compound 6

Atomic Type	Coordinates (Angstroms) X	Y	Z
C	-1.826296	-1.433392	0.014540
C	-2.758054	-0.464475	-0.244334
C	-2.077170	0.773269	-0.260996
C	-0.762171	0.500942	-0.017002
N	-0.618235	-0.848480	0.138976
H	-1.941610	-2.493473	0.112310
H	-3.802609	-0.628324	-0.409826
H	-2.486130	1.746042	-0.437103
C	0.645823	-1.433413	0.527840
H	0.709576	-1.487689	1.611732
H	0.733621	-2.430114	0.121466
C	0.390057	1.394930	0.096321
O	1.588557	0.781778	0.235909
C	1.744545	-0.558707	-0.003159
O	0.336442	2.568640	0.092157
C	2.832473	-0.985963	-0.599438
H	3.589023	-0.294287	-0.918160
H	2.990539	-2.035292	-0.760414

3.2. Theoretical Matrix for compound 7

Atomic Type	Coordinates (Angstroms) X	Y	Z
C	1.802884	-1.535879	0.000176
C	2.813882	-0.612412	0.000109

C	2.215997	0.667151	0.000287
C	0.865869	0.467882	0.000078
N	0.624054	-0.879577	-0.000341
H	1.840817	-2.605470	-0.000061
H	3.860956	-0.833465	-0.000142
H	2.702811	1.619640	0.000259
C	-0.683969	-1.370553	-0.000407
H	-0.820081	-2.431213	-0.000437
C	-0.249704	1.404942	-0.000083
O	-1.479753	0.834394	-0.000083
C	-1.685407	-0.513161	0.000164
O	-0.158224	2.577176	-0.000130
C	-3.136195	-0.855873	0.000280
H	-3.615976	-0.433280	-0.876266
H	-3.277760	-1.929231	-0.000854
H	-3.615476	-0.435079	0.877962

3.3. Theoretical Matrix for compound 30

Atomic Type	Coordinates (Angstroms) X	Y	Z
C	-3.703792	-1.369681	-0.304231
C	-4.489360	-0.383585	-0.839106
C	-3.772089	0.827244	-0.723677
C	-2.581725	0.522275	-0.128402
N	-2.546412	-0.821410	0.113835
H	-3.889758	-2.418992	-0.199427
H	-5.459730	-0.519902	-1.269363
H	-4.074944	1.803226	-1.040432
C	-1.454944	-1.429790	0.844895
H	-1.318410	-2.450128	0.519460
C	-1.461025	1.377790	0.253442
O	-0.373270	0.727314	0.733326
C	-0.214613	-0.625423	0.582888
O	-1.448474	2.551475	0.192447
C	0.959246	-1.157588	0.296268
H	0.963996	-2.231538	0.233696
C	2.273740	-0.527259	0.057384
C	3.323943	-1.367771	-0.307066
C	2.531208	0.839655	0.173425
C	4.590135	-0.869900	-0.554438
H	3.147738	-2.425476	-0.399432
C	3.798616	1.334366	-0.073826
H	1.749403	1.513601	0.455602
C	4.832076	0.487095	-0.438689
H	5.382500	-1.540430	-0.835553
H	3.977694	2.390549	0.020297
H	5.814222	0.881324	-0.629420
H	-1.682369	-1.427255	1.908335

3.4. Theoretical Matrix for compound 31

Atomic Type	X	Y	Z
C	3.402761	-1.578855	-0.338072
C	4.334361	-0.718876	-0.854575
C	3.793519	0.583834	-0.778574
C	2.555063	0.461180	-0.219011
N	2.325854	-0.862370	0.046661
H	3.431153	-2.642151	-0.219522
H	5.292699	-0.996173	-1.241755
H	4.245343	1.501778	-1.090732
C	1.122905	-1.277398	0.622506
H	0.988689	-2.323026	0.801820
C	1.540065	1.453196	0.110074
O	0.413291	0.954998	0.676371
C	0.210493	-0.370728	0.911776
O	1.631120	2.611697	-0.068543
C	-1.131404	-0.642876	1.522454
H	-1.160314	-1.678435	1.839159
H	-1.223076	-0.028742	2.412700
C	-2.288727	-0.358566	0.580747
C	-2.812882	0.921703	0.462190
C	-2.836881	-1.379080	-0.183839
C	-3.862493	1.174769	-0.403996
H	-2.393022	1.724766	1.040674
C	-3.888264	-1.129156	-1.049490
H	-2.443728	-2.378147	-0.102550
C	-4.404041	0.150198	-1.161863
H	-4.256110	2.172142	-0.486239
H	-4.303757	-1.932196	-1.631976
H	-5.220997	0.347148	-1.832789
