# Supplementary material for: Gold-catalyzed formation of pyrrolo- and indolo-oxazin-1-one derivatives: The key structure of some marine natural products
Source: Beilstein J Org Chem. 2015 May 28;11:897–905. doi: 10.3762/bjoc.11.101 (PMC4464457; doi:10.3762/bjoc.11.101)
Supplement: File 2 — NMR spectra. [file Beilstein_J_Org_Chem-11-897-s002.pdf]

Supporting Information
for
Gold-catalyzed formation of pyrrolo- and indolo-oxazin-1-one derivatives: The key structure of some marine natural products

Sultan Taskaya,[1] Nurettin Menges,[1,2,*] and Metin Balci[1,*]

Address: [1]Middle East Technical University, Department of Chemistry, Ankara, Turkey, 06800 and [2]Yüzüncü Yil University, Faculty of Pharmacy, Van, Turkey, 65100

Email: Nurettin Menges - nurettinmenges@gmail.com; Metin Balci - mbalci@metu.edu.tr

*Corresponding author

NMR spectra

Supporting Information

NMR Spectra

Table of Figures

Figure 1: ^{1}H and ^{13}C NMR Spectra of **13** in $CDCl_3$ S2
Figure 2: ^{1}H and ^{13}C NMR Spectra of **14** in $CDCl_3$ S3
Figure 3: ^{1}H and ^{13}C NMR Spectra of **15** in $CDCl_3$ S4
Figure 4: ^{1}H and ^{13}C NMR Spectra of **7** in $CDCl_3$ S5
Figure 5: DEPT 90 and DEPT 135 Spectra of **7** in $CDCl_3$ S6
Figure 6: COSY and HMBC Spectra of **7** in $CDCl_3$ S7
Figure 7: HSQC Spectra of **7** in $CDCl_3$ S8
Figure 8: ^{1}H and ^{13}C NMR Spectra of **6** in $CDCl_3$ S9
Figure 9: ^{1}H and ^{13}C NMR Spectra of **16** in $CDCl_3$ S10
Figure 10: ^{1}H and ^{13}C NMR Spectra of **17** in CD_3OD S11
Figure 11: ^{1}H and ^{13}C NMR Spectra of **18** in $CDCl_3$ S12
Figure 12: ^{1}H and ^{13}C NMR Spectra of **19** in $CDCl_3$ S13
Figure 13: ^{1}H and ^{13}C NMR Spectra of **20** in $CDCl_3$ S14
Figure 14: ^{1}H and ^{13}C NMR Spectra of **21** in CD_3OD S15
Figure 15: ^{1}H and ^{13}C NMR Spectra of **22** in $CDCl_3$ S16
Figure 16: ^{1}H and ^{13}C NMR Spectra of **23** in $CDCl_3$ S17
Figure 17: ^{1}H and ^{13}C NMR Spectra of **24** in $CDCl_3$ S18
Figure 18: ^{1}H and ^{13}C NMR Spectra of **25** in CD_3COCD_3 .... S19
Figure 19: ^{1}H and ^{13}C NMR Spectra of **27** in $CDCl_3$ S20
Figure 20: ^{1}H and ^{13}C NMR Spectra of **28** in $CDCl_3$ S21
Figure 21: ^{1}H and ^{13}C NMR Spectra of **29** in $CDCl_3$ S22
Figure 22: ^{1}H and ^{13}C NMR Spectra of **30** in $CDCl_3$ S23
Figure 23: ^{1}H and ^{13}C NMR Spectra of **32** in $CDCl_3$ S24
Figure 24: ^{1}H and ^{13}C NMR Spectra of **33** in $CDCl_3$ S25
Figure 25: ^{1}H and ^{13}C NMR Spectra of **34** in $CDCl_3$ S26
Figure 26: ^{1}H and ^{13}C NMR Spectra of **36** in $CDCl_3$ S27
Figure 27: ^{1}H and ^{13}C NMR Spectra of **37** in CD_3OD S28
Figure 28: ^{1}H and ^{13}C NMR Spectra of **38** in $CDCl_3$ S29
Figure 29: ^{1}H and ^{13}C NMR Spectra of **39** in $CDCl_3$ S30
Figure 30: ^{1}H and ^{13}C NMR Spectra of **40** in $CDCl_3$ S31
Figure 31: ^{1}H and ^{13}C NMR Spectra of **41** in $CDCl_3$ S32
Figure 32: ^{1}H and ^{13}C NMR Spectra of **42** in $CDCl_3$ S33
Figure 33: ^{1}H and ^{13}C NMR Spectra of **43** in $CDCl_3$ S34
Figure 34: ^{1}H and ^{13}C NMR Spectra of **44** in $CDCl_3$ S35
Figure 35: ^{1}H and ^{13}C NMR Spectra of **45** in CD_3COCD_3 .... S36
Figure 36: ^{1}H and ^{13}C NMR Spectra of **46** in $CDCl_3$ S37
Figure 37: ^{1}H and ^{13}C NMR Spectra of **48** in $CDCl_3$ S38
Figure 38: ^{1}H NMR Spectra of the reaction of **15** with gold(I) in chloroform in the presence of ethanol. S39
Figure 39: ^{1}H and ^{13}C NMR Spectra of the reaction of **15** with gold(I) in the presence of CD_3OD. S40
Figure 40: ^{1}H NMR Spectra of the reaction of **15** with gold(I) in chloroform S41
Figure 41: ^{1}H NMR Spectra of the reaction of **7** with gold(I) in the presence of EtOH S42
Figure 42: ^{1}H and ^{13}C NMR Spectra of **50a** and **50b** in $CDCl_3$ S43

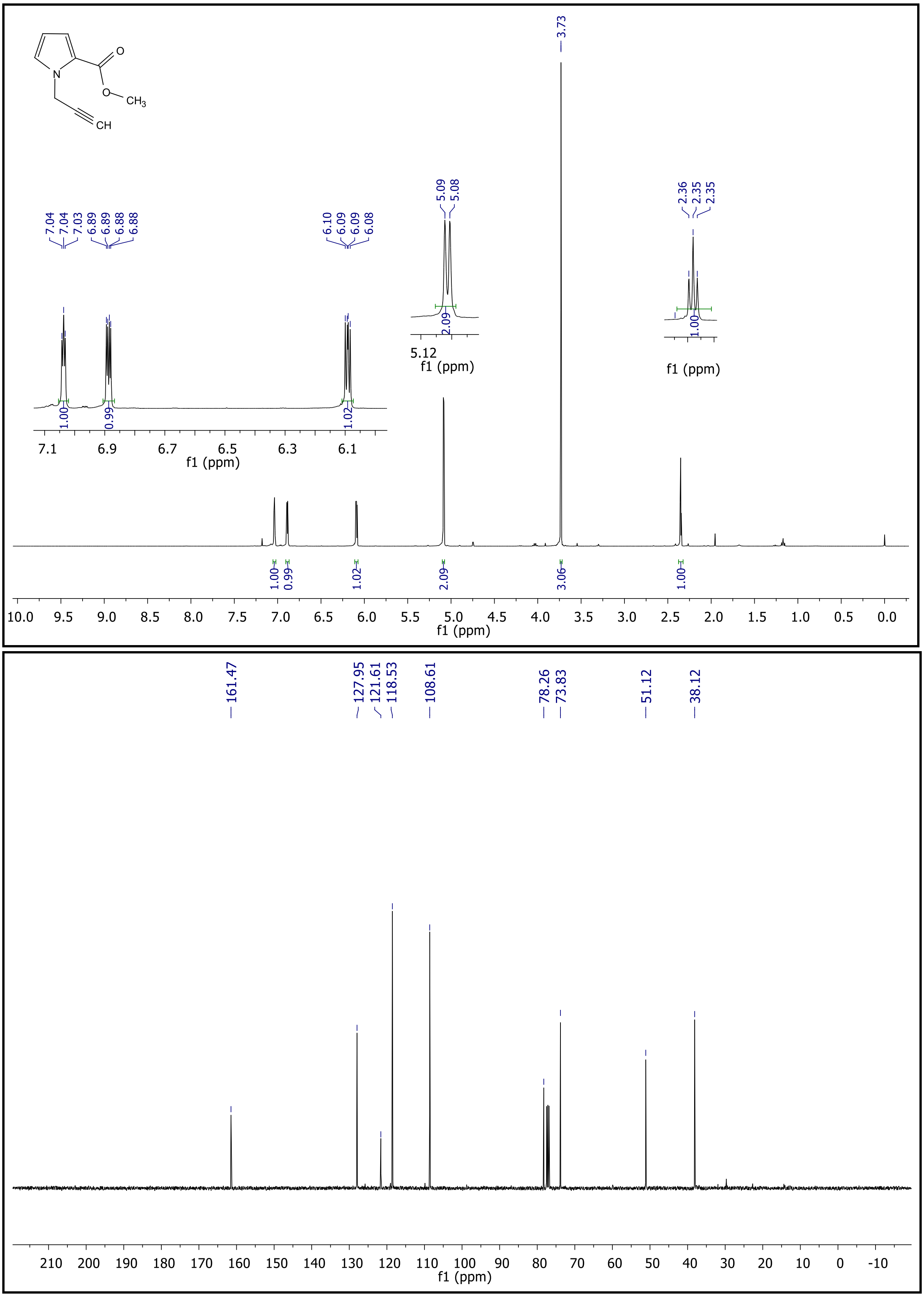

Figure 1: ^{1}H and ^{13}C NMR Spectra of **13** in $CDCl_3$

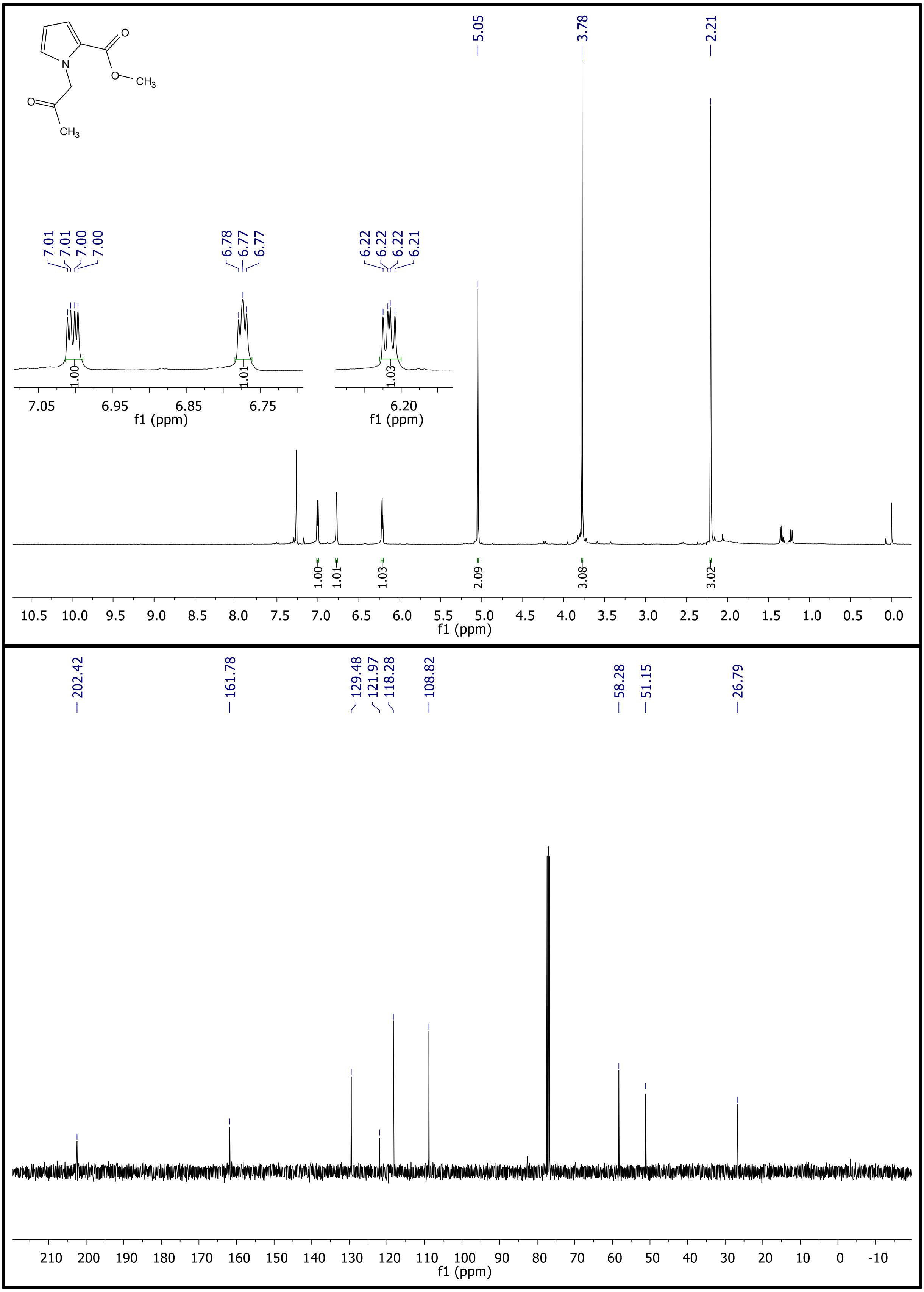

Figure 2: ^{1}H and ^{13}C NMR Spectra of **14** in $CDCl_3$

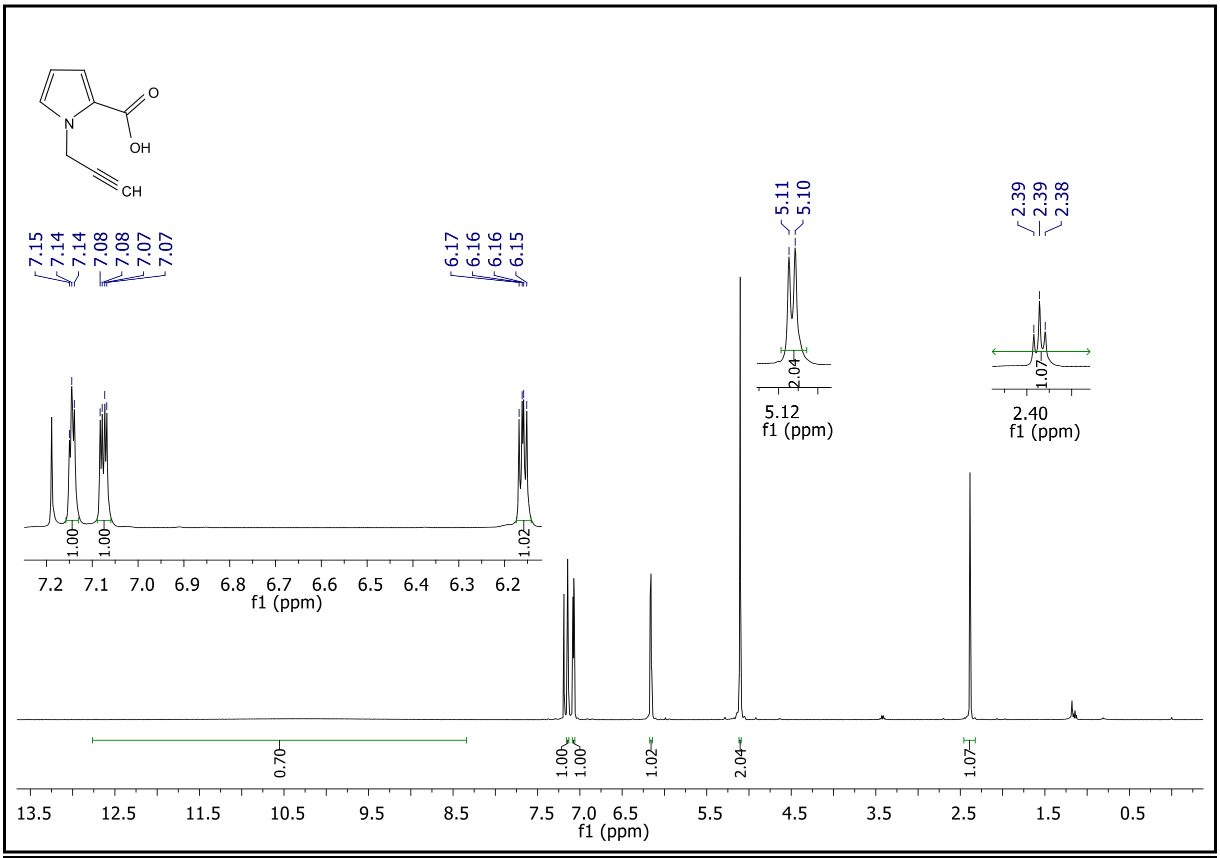

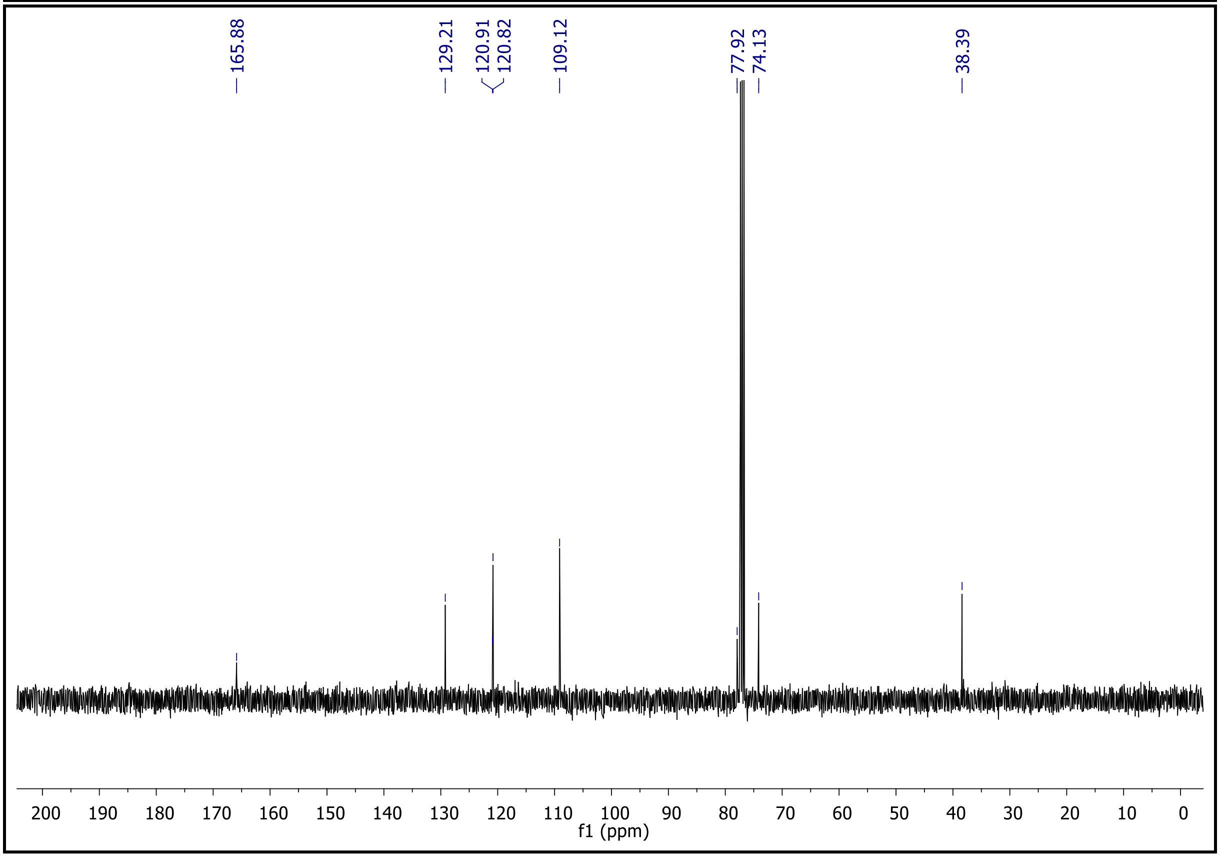

Figure 3: ^{1}H and ^{13}C NMR Spectra of **15** in $CDCl_3$

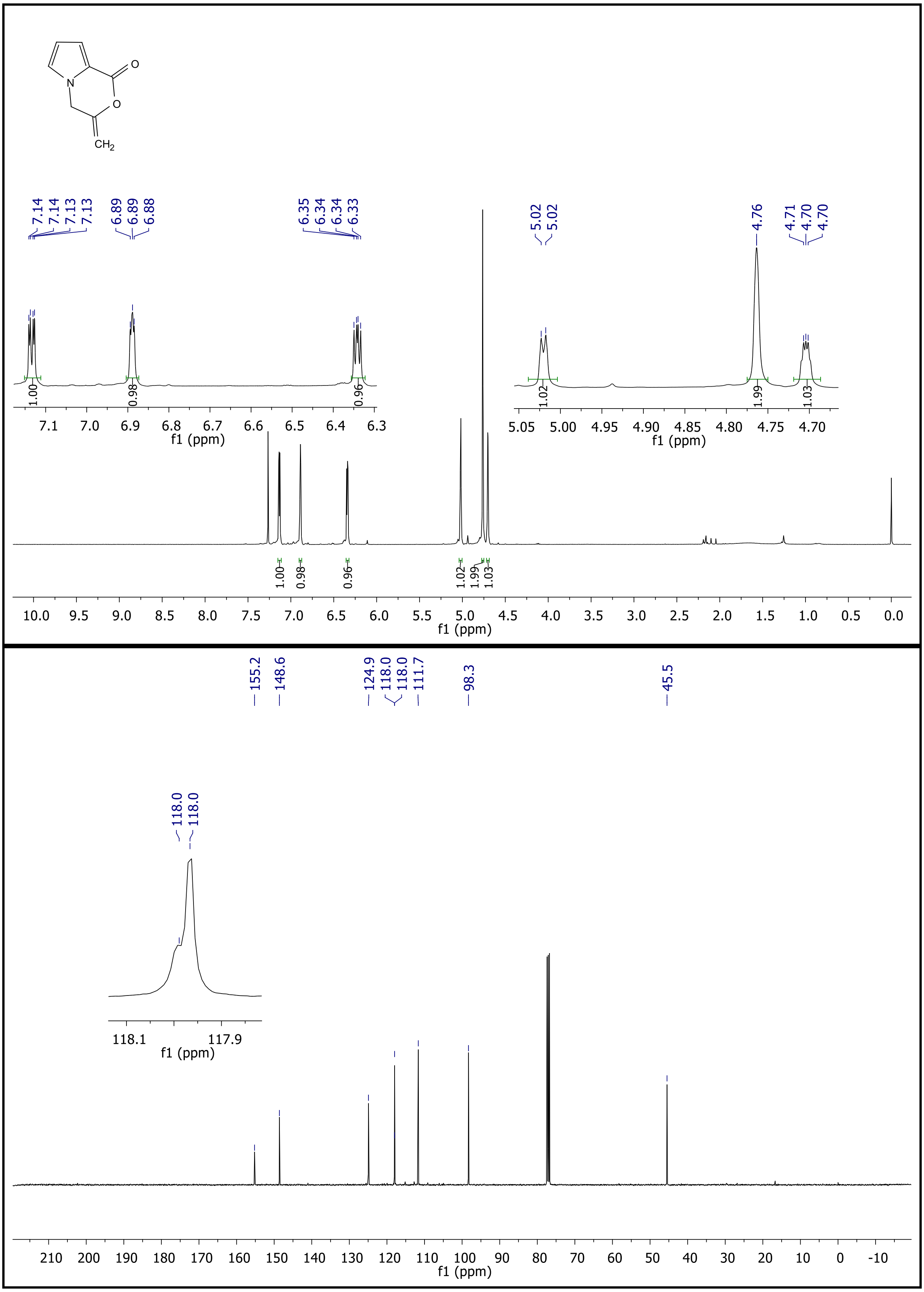

Figure 4: ^{1}H and ^{13}C NMR Spectra of **7** in $CDCl_3$

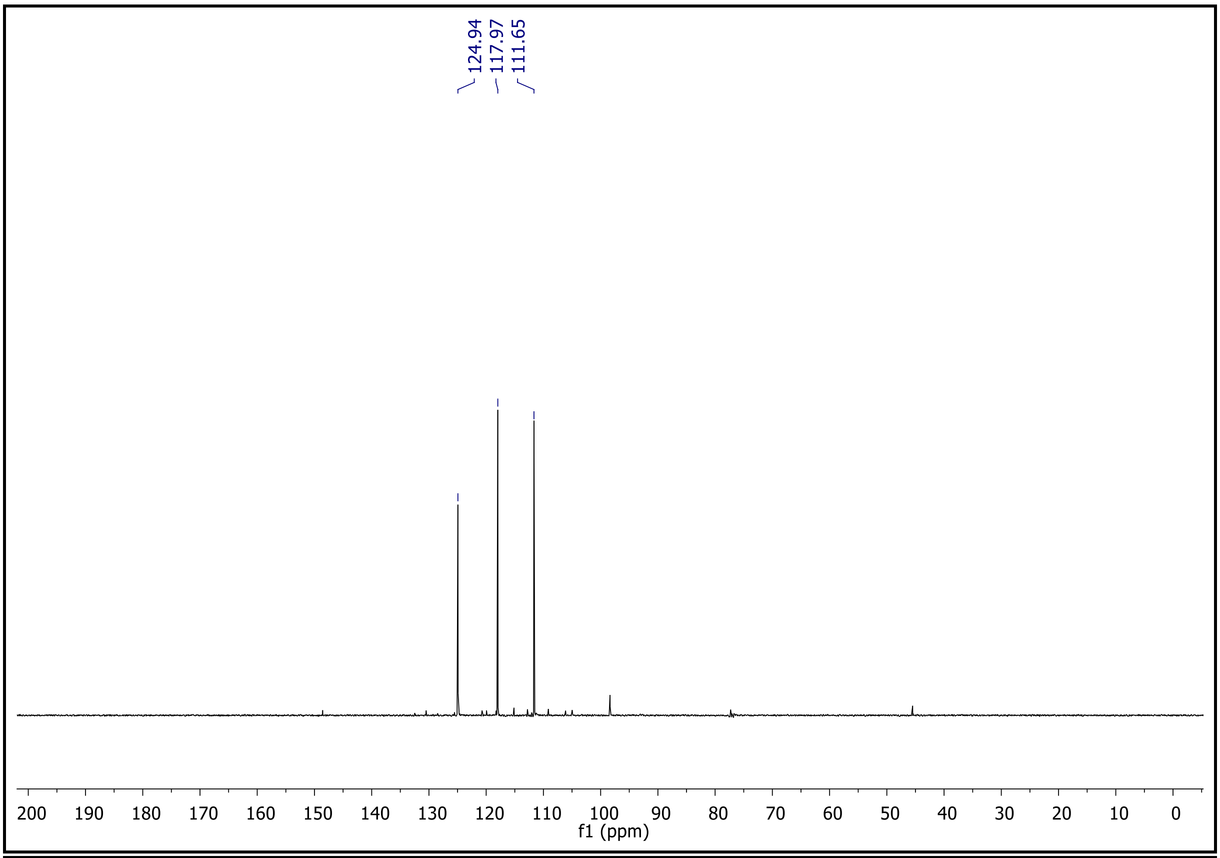

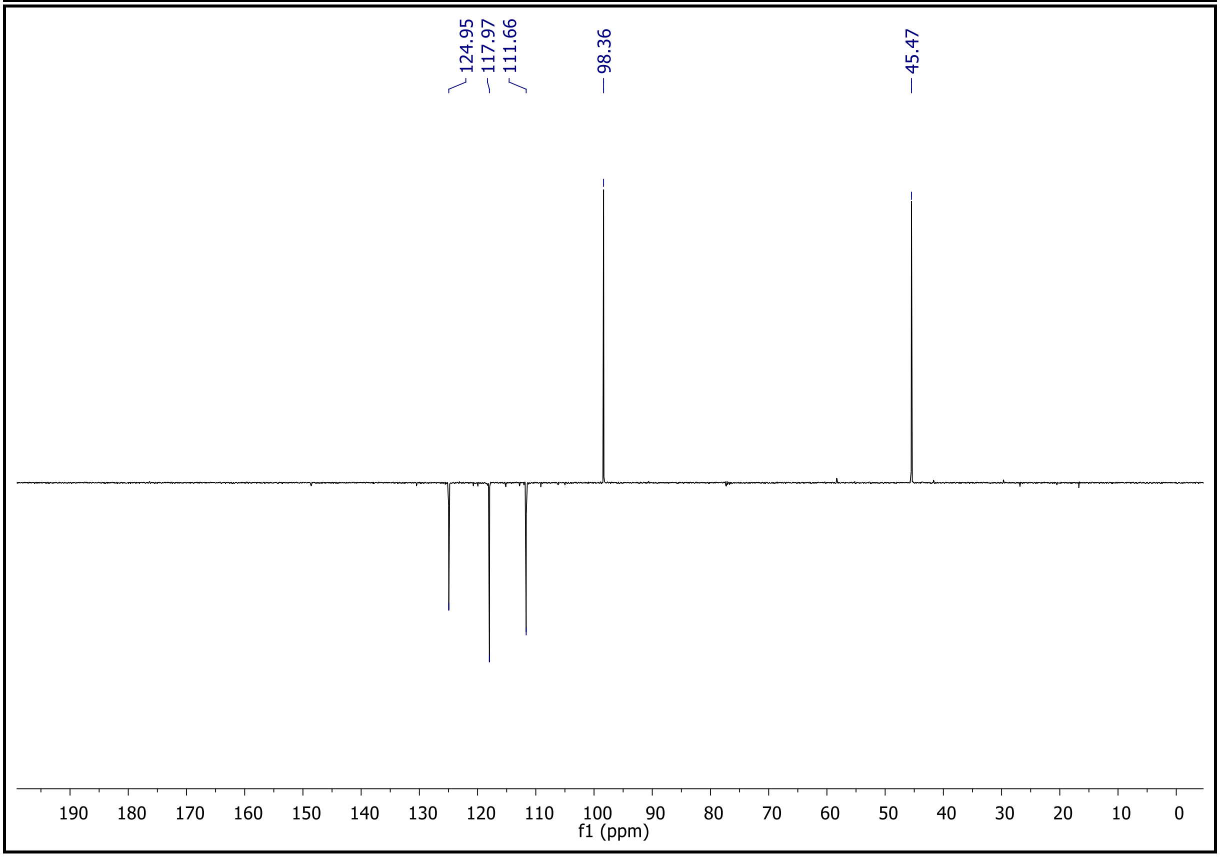

Figure 5: DEPT 90 and DEPT 135 Spectra of **7** in $CDCl_3$

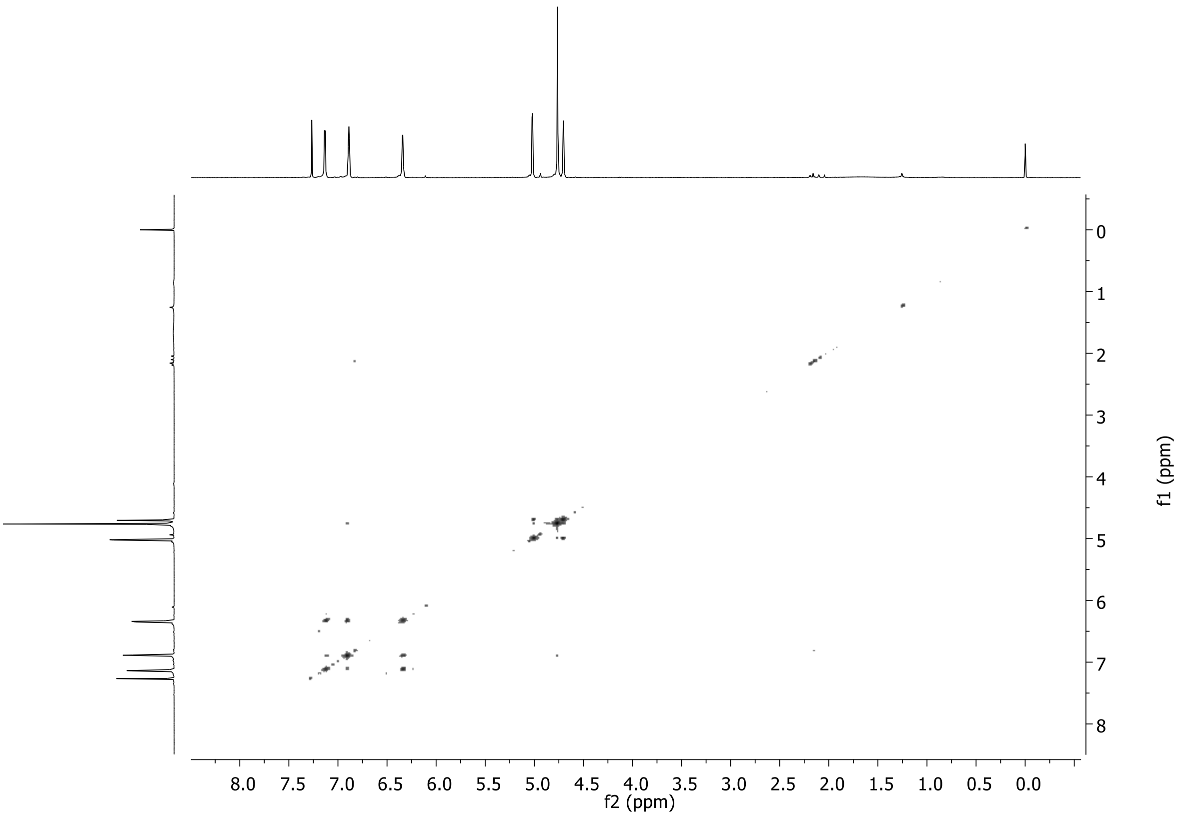

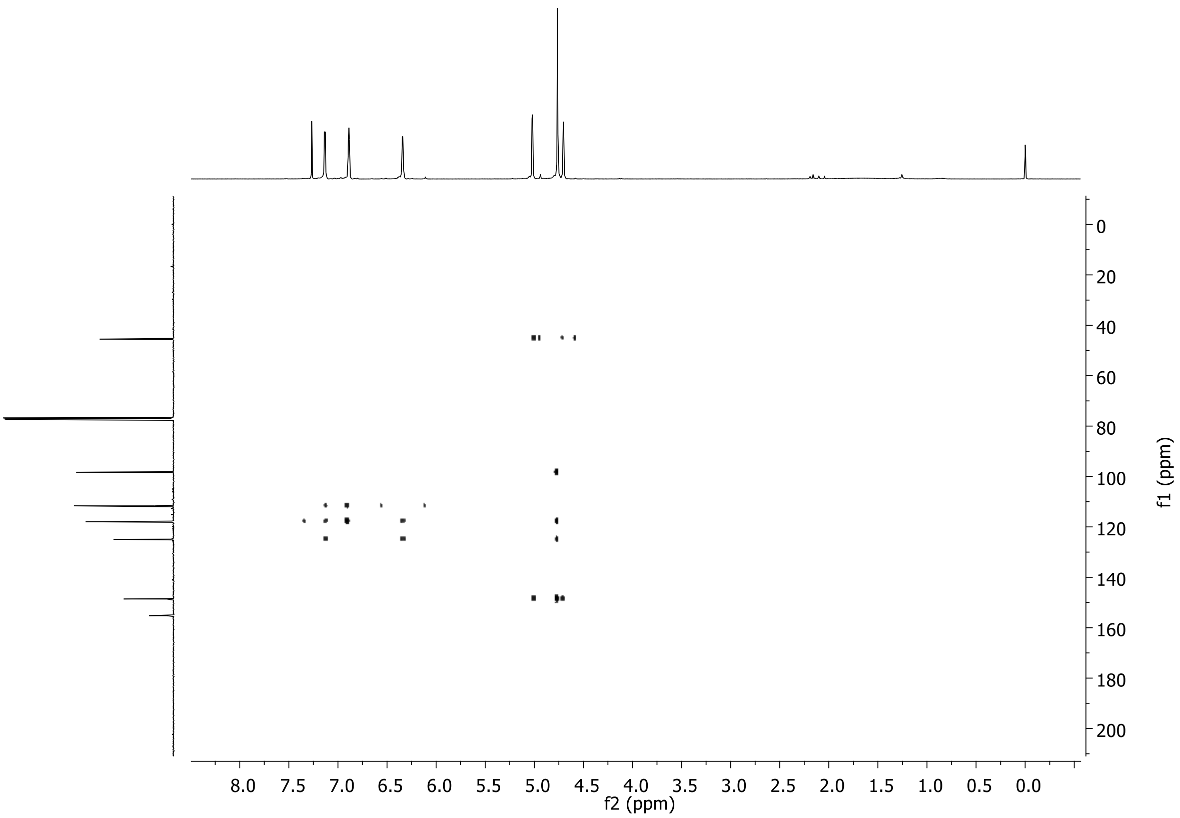

Figure 6: COSY and HMBC Spectra of **7** in $CDCl_3$

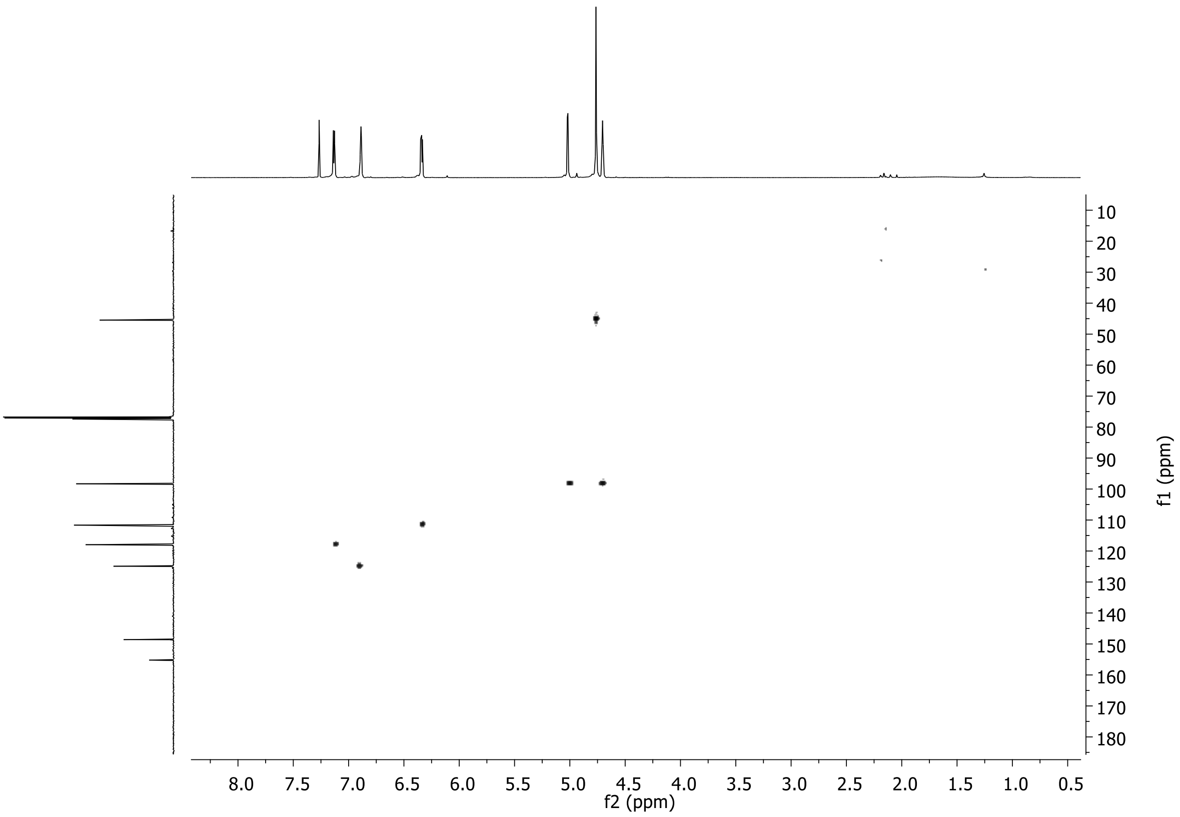

Figure 7: HSQC Spectra of **7** in $CDCl_3$

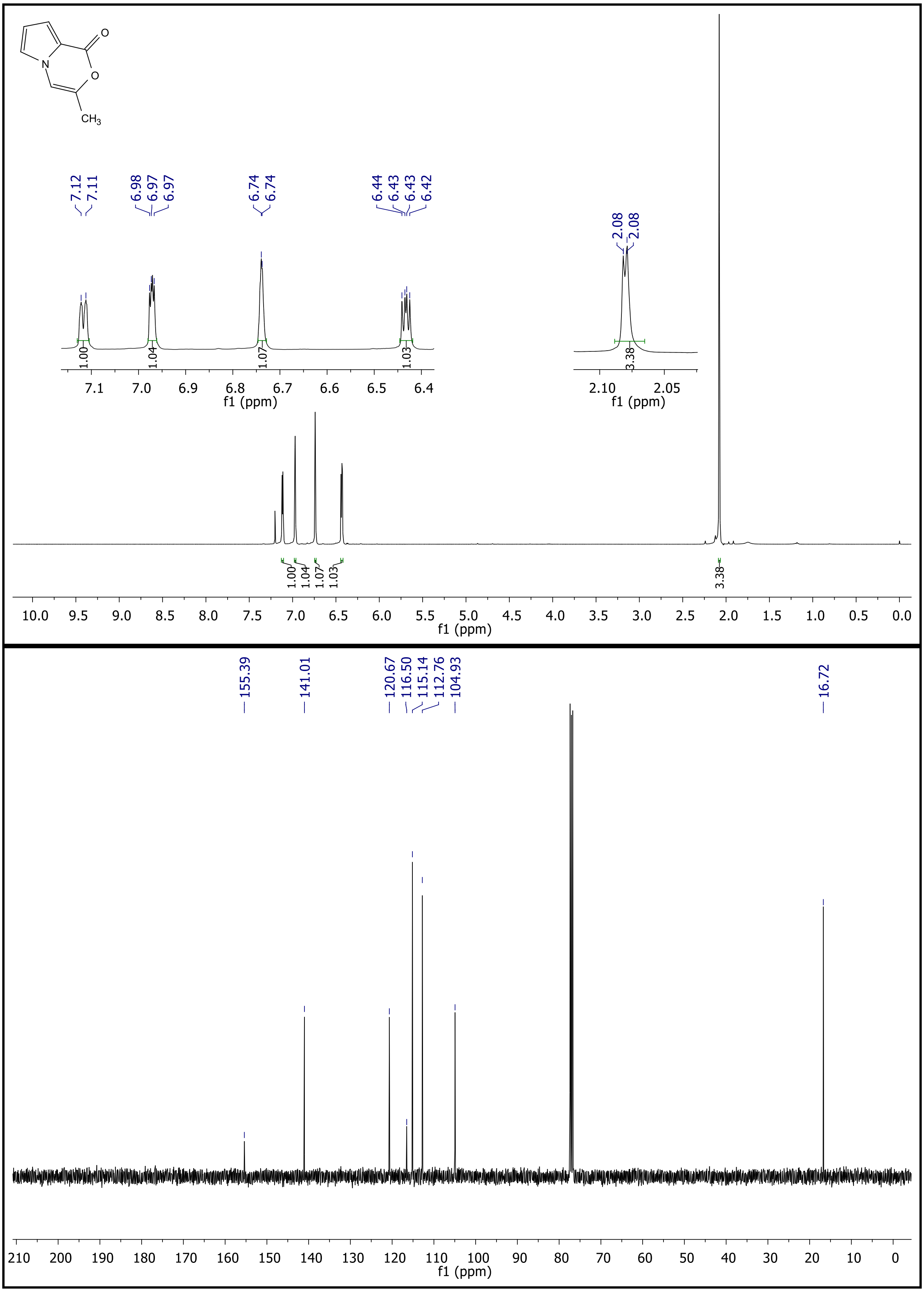

Figure 8: ^{1}H and ^{13}C NMR Spectra of **6** in $CDCl_3$

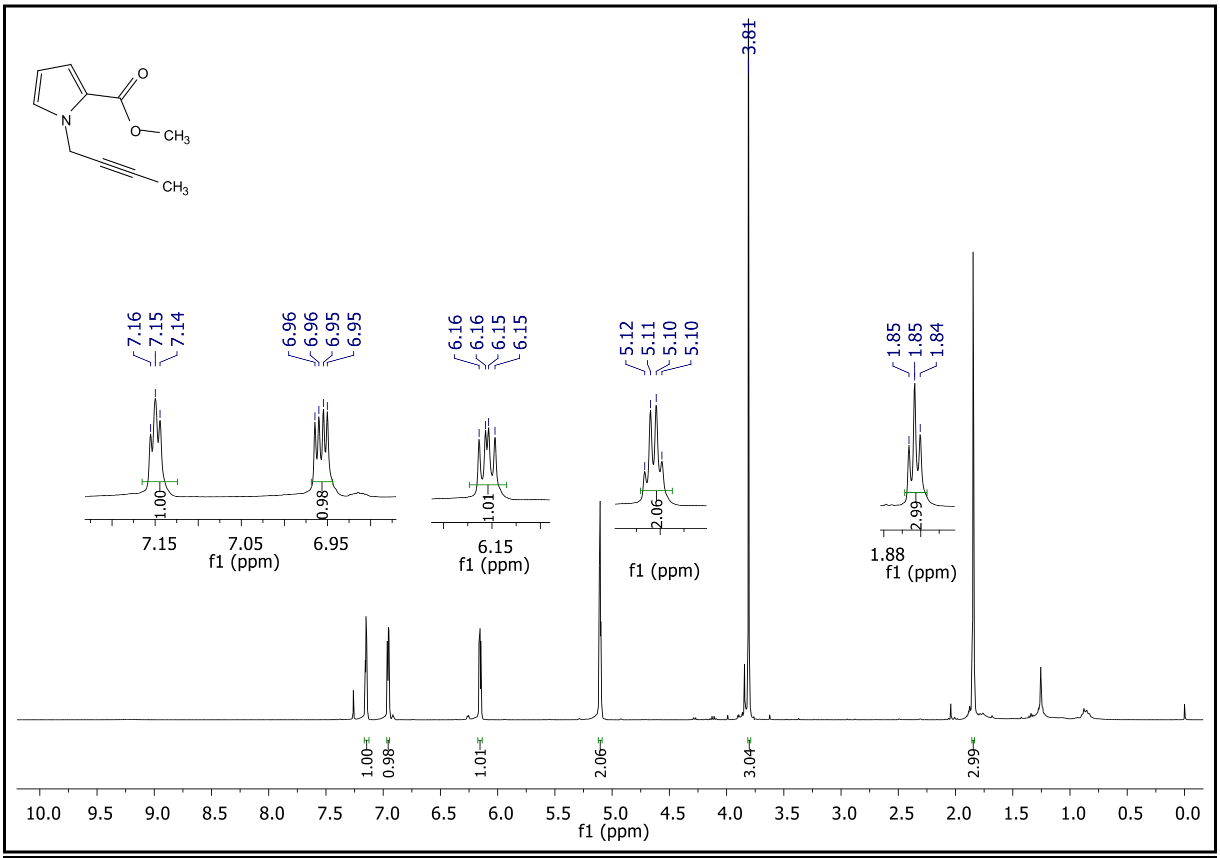

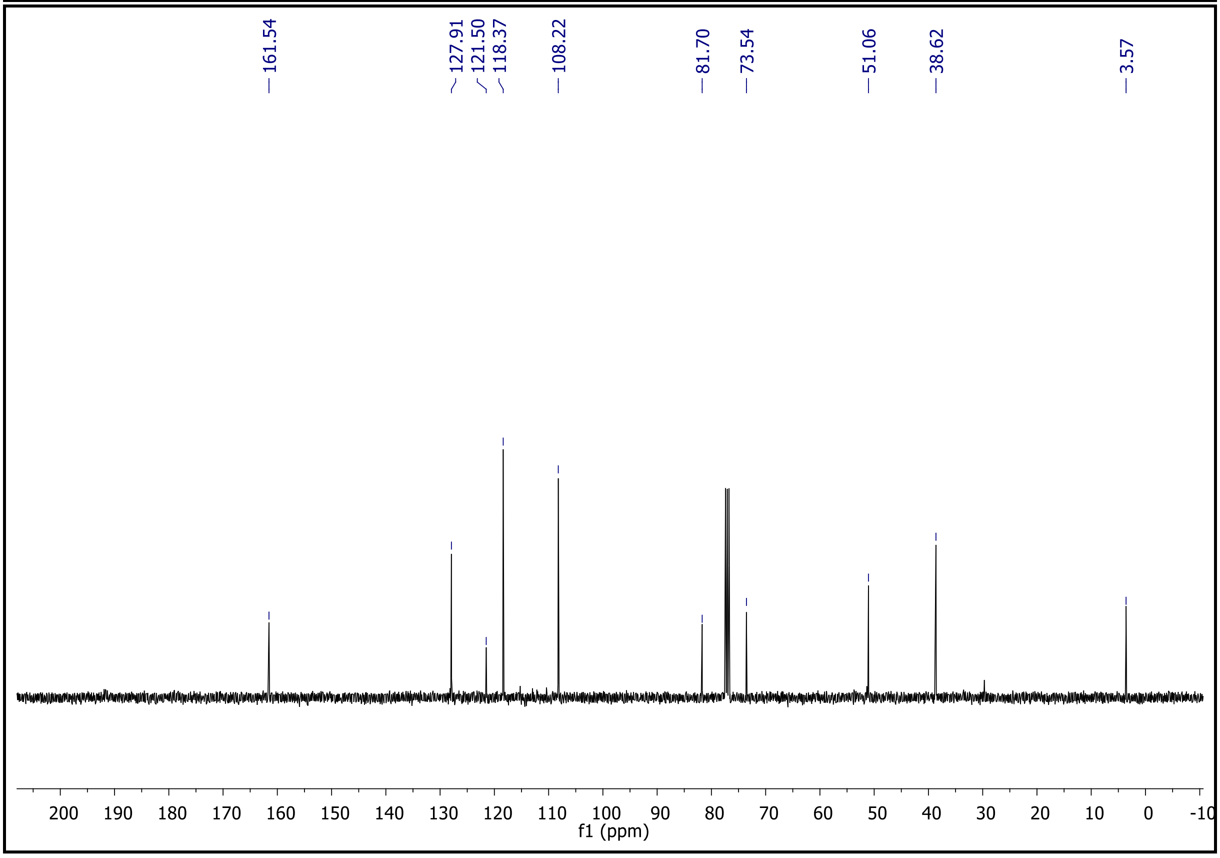

Figure 9: ^{1}H and ^{13}C NMR Spectra of **16** in $CDCl_3$

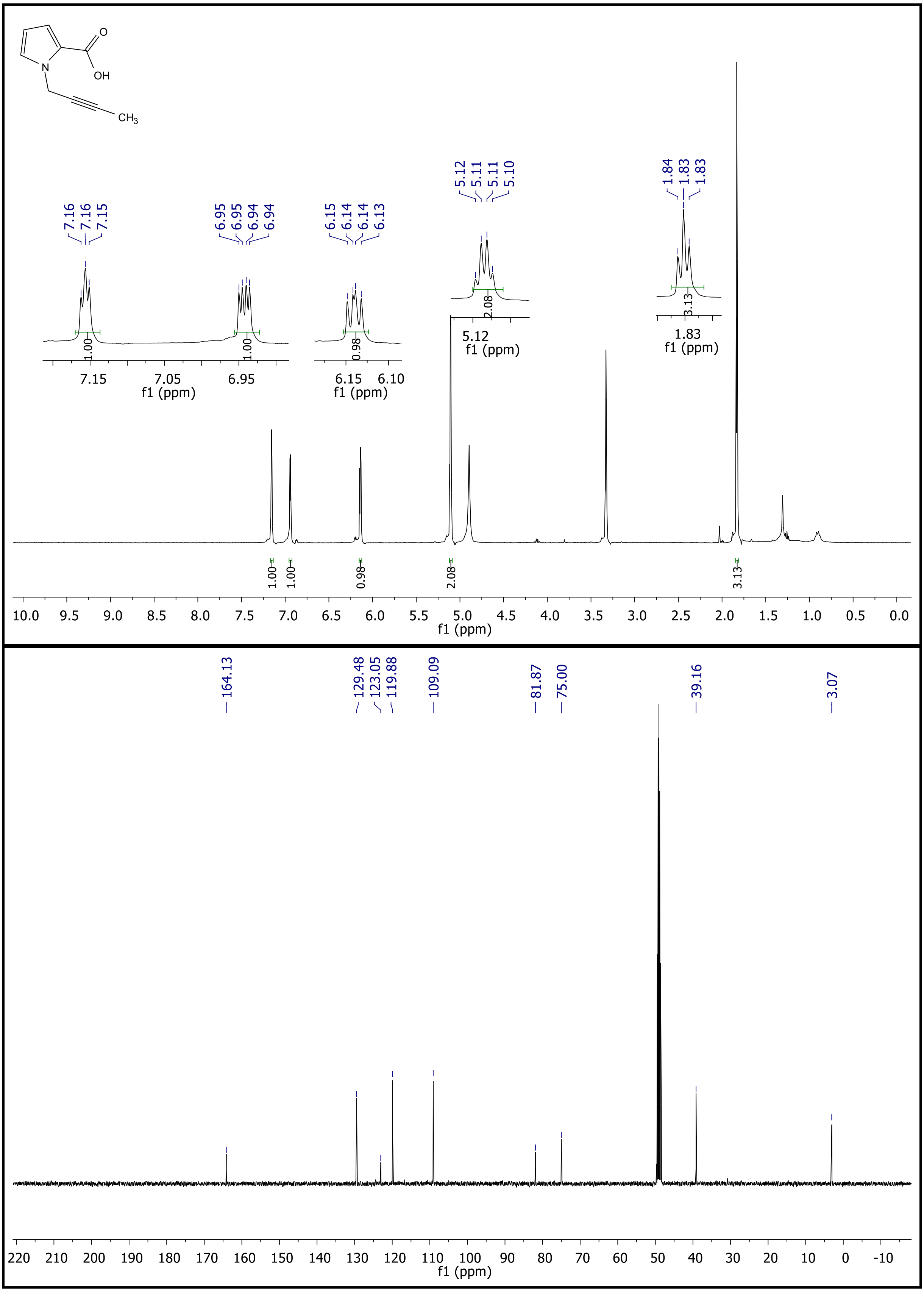

Figure 10: ^{1}H and ^{13}C NMR Spectra of **17** in CD_3OD

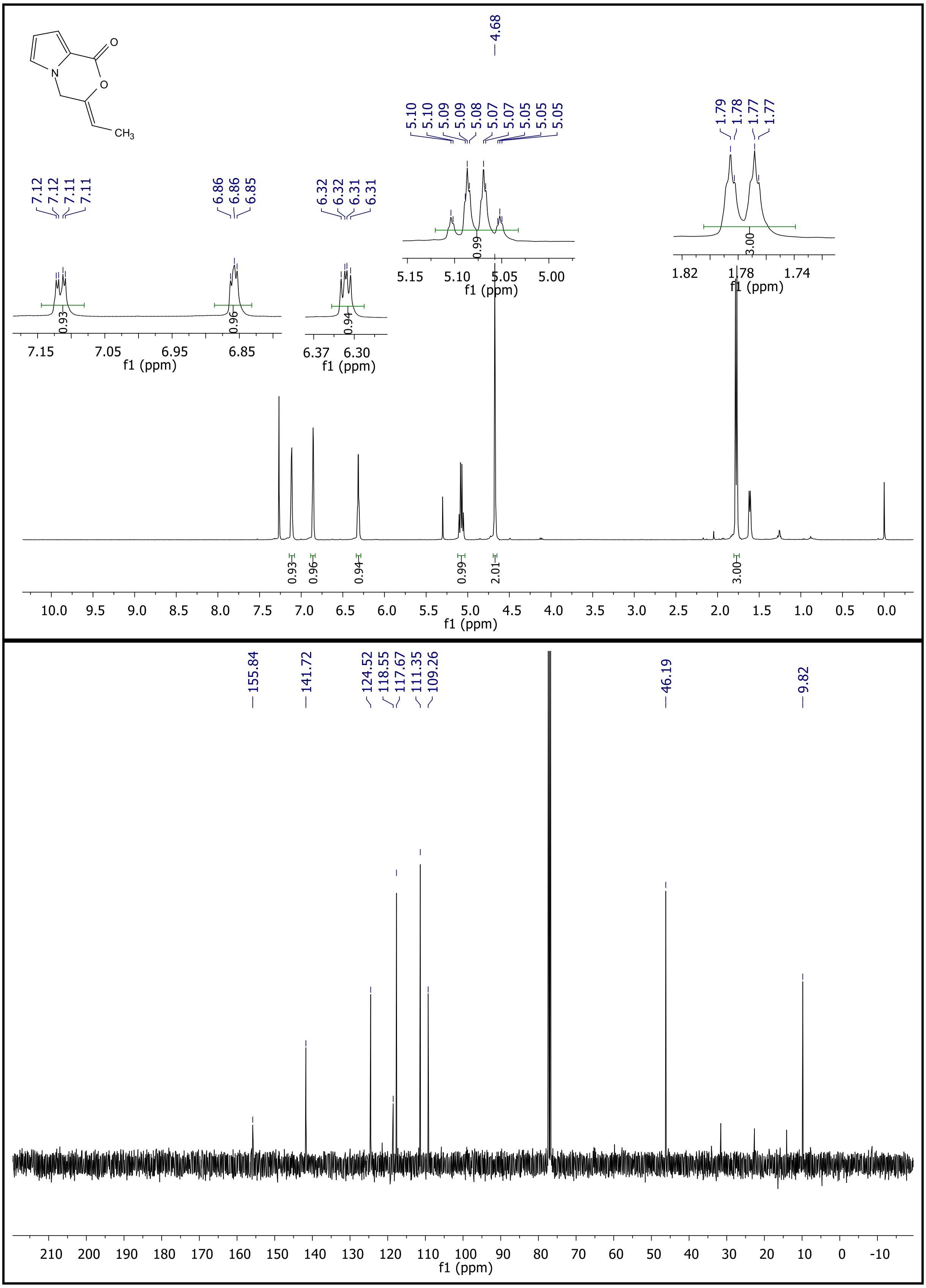

Figure 11: ^{1}H and ^{13}C NMR Spectra of **18** in $CDCl_3$

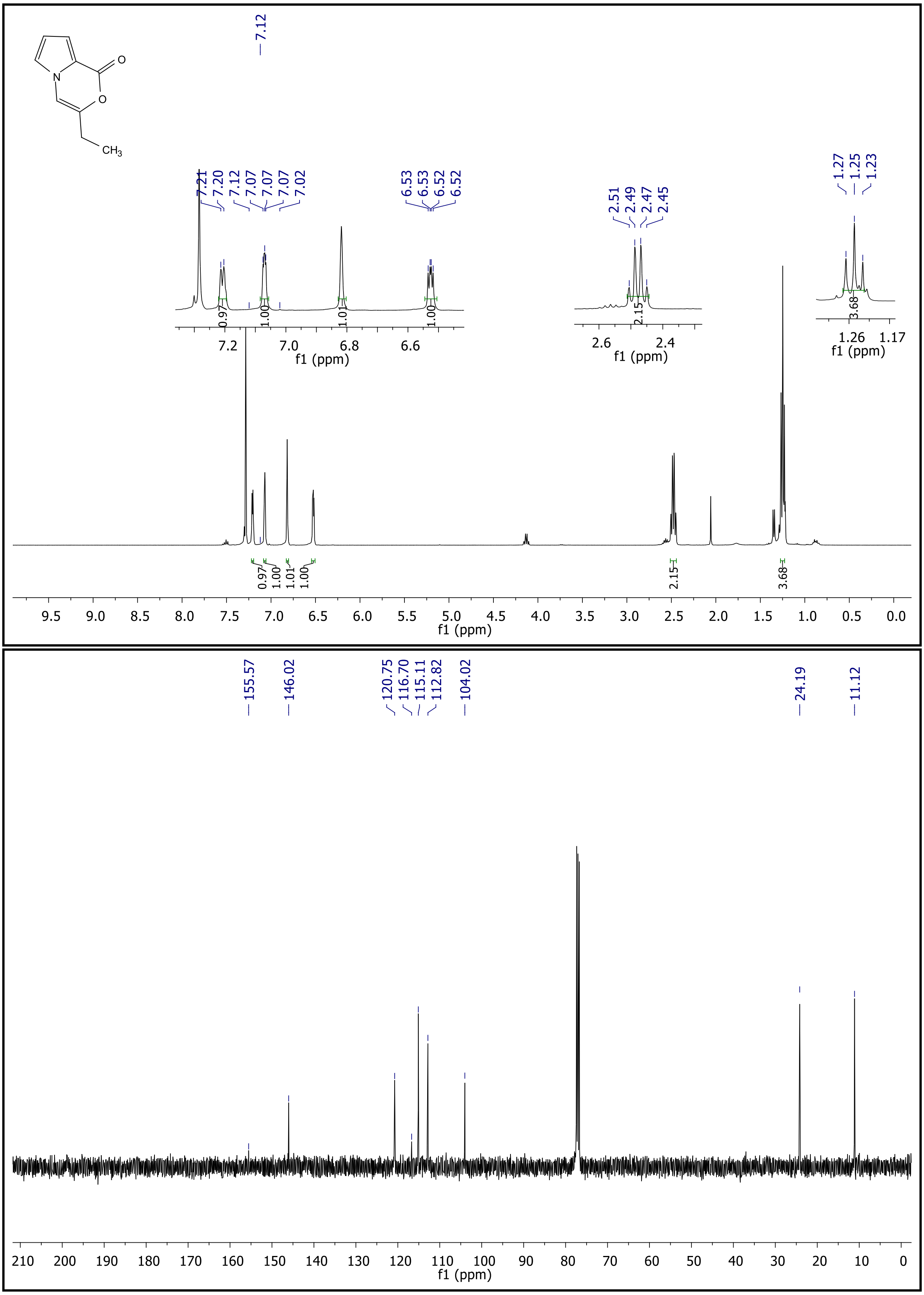

Figure 12: ^{1}H and ^{13}C NMR Spectra of **19** in $CDCl_3$

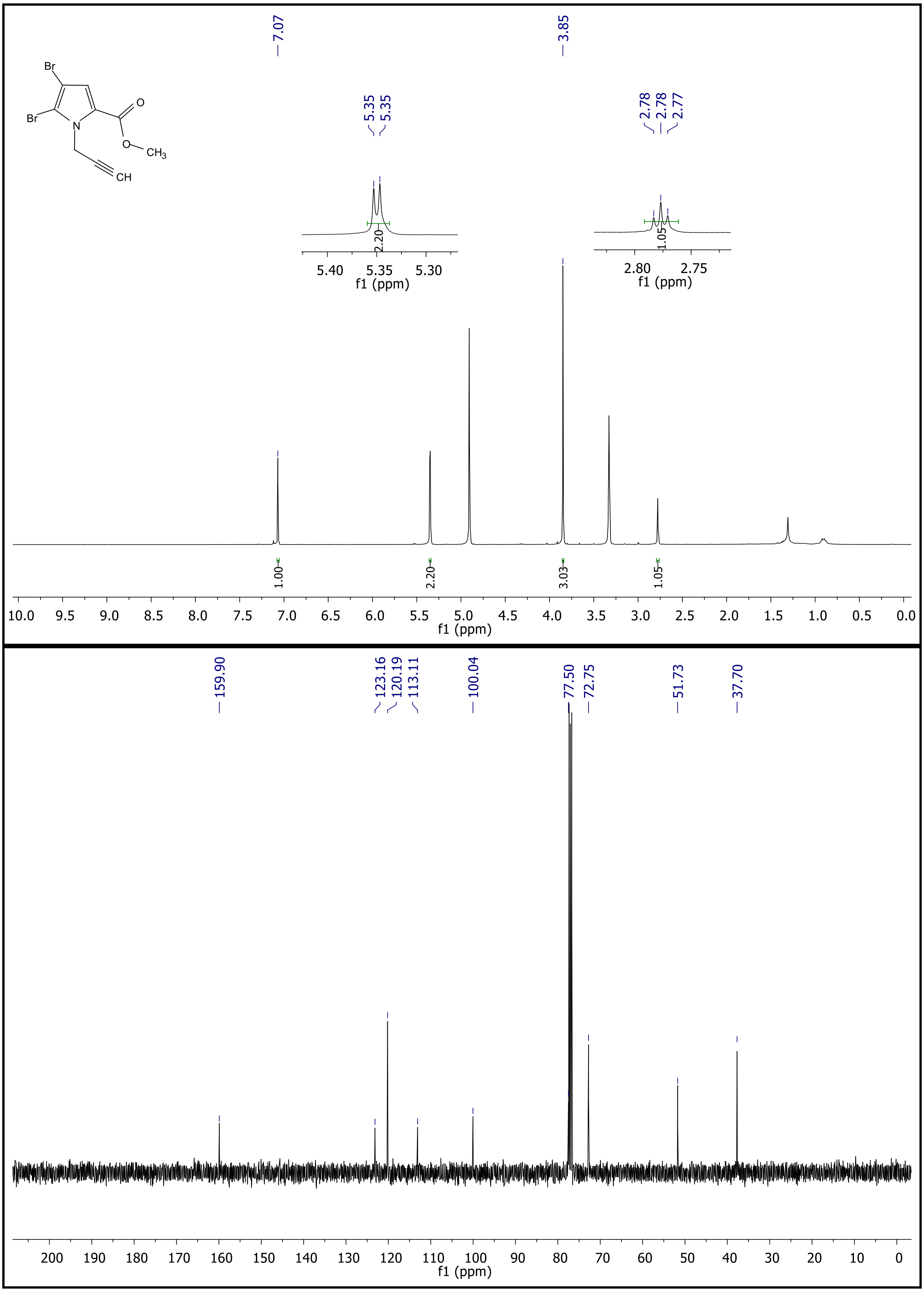

Figure 13: ^{1}H and ^{13}C NMR Spectra of **20** in $CDCl_3$

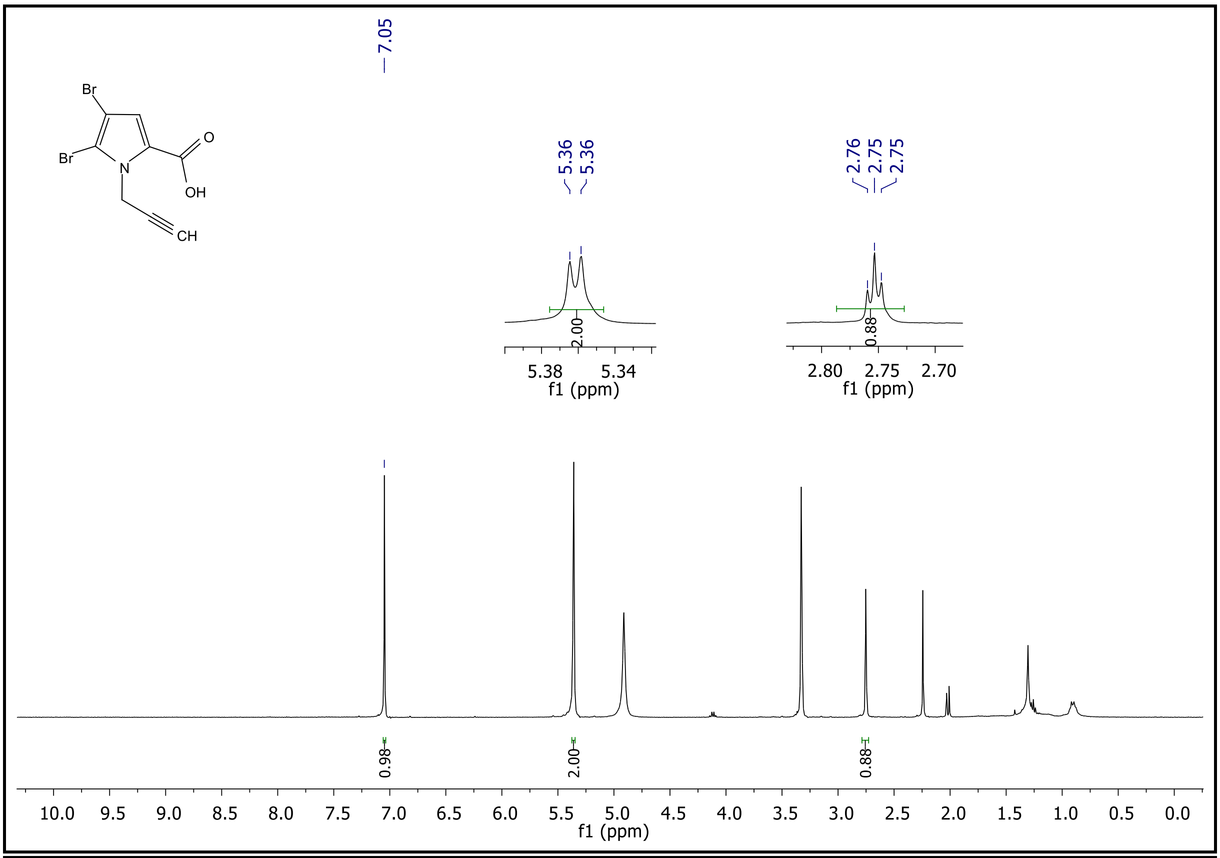

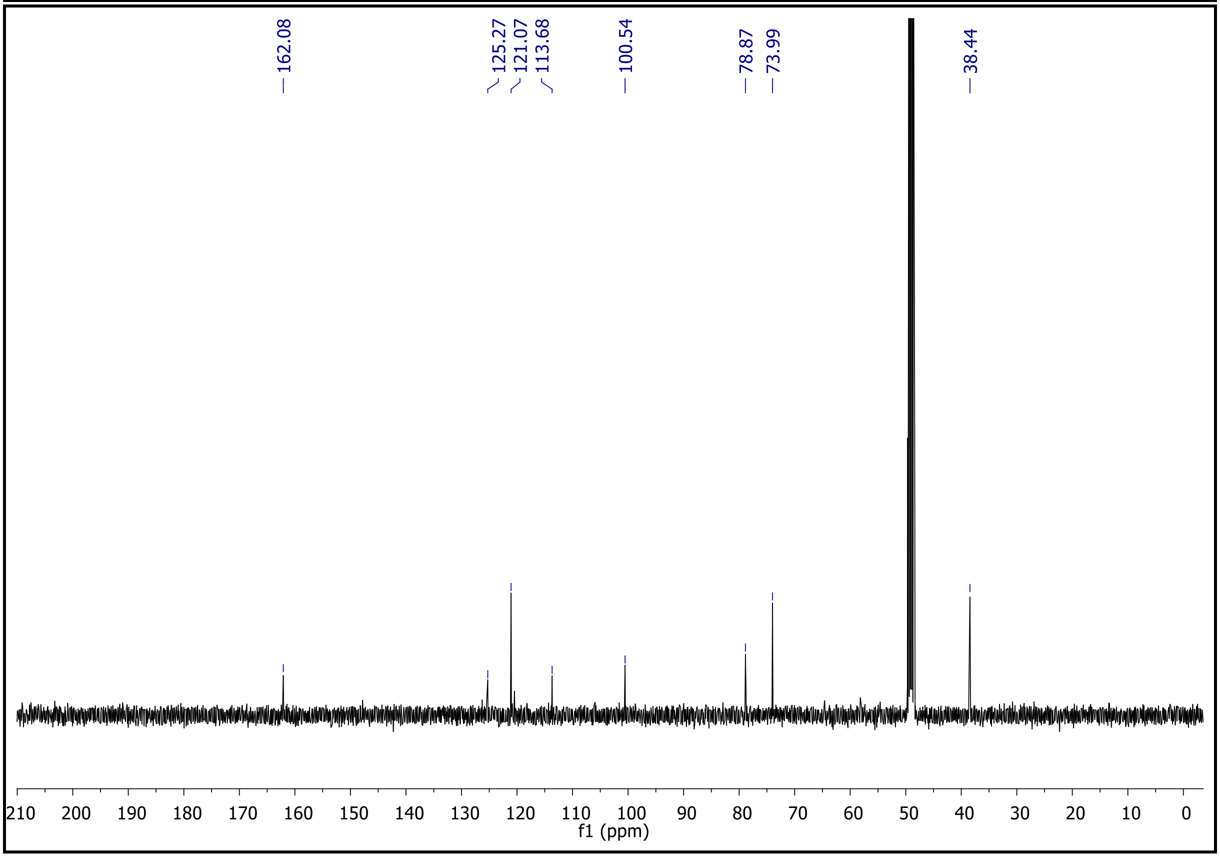

Figure 14: ^{1}H and ^{13}C NMR Spectra of **21** in CD_3OD

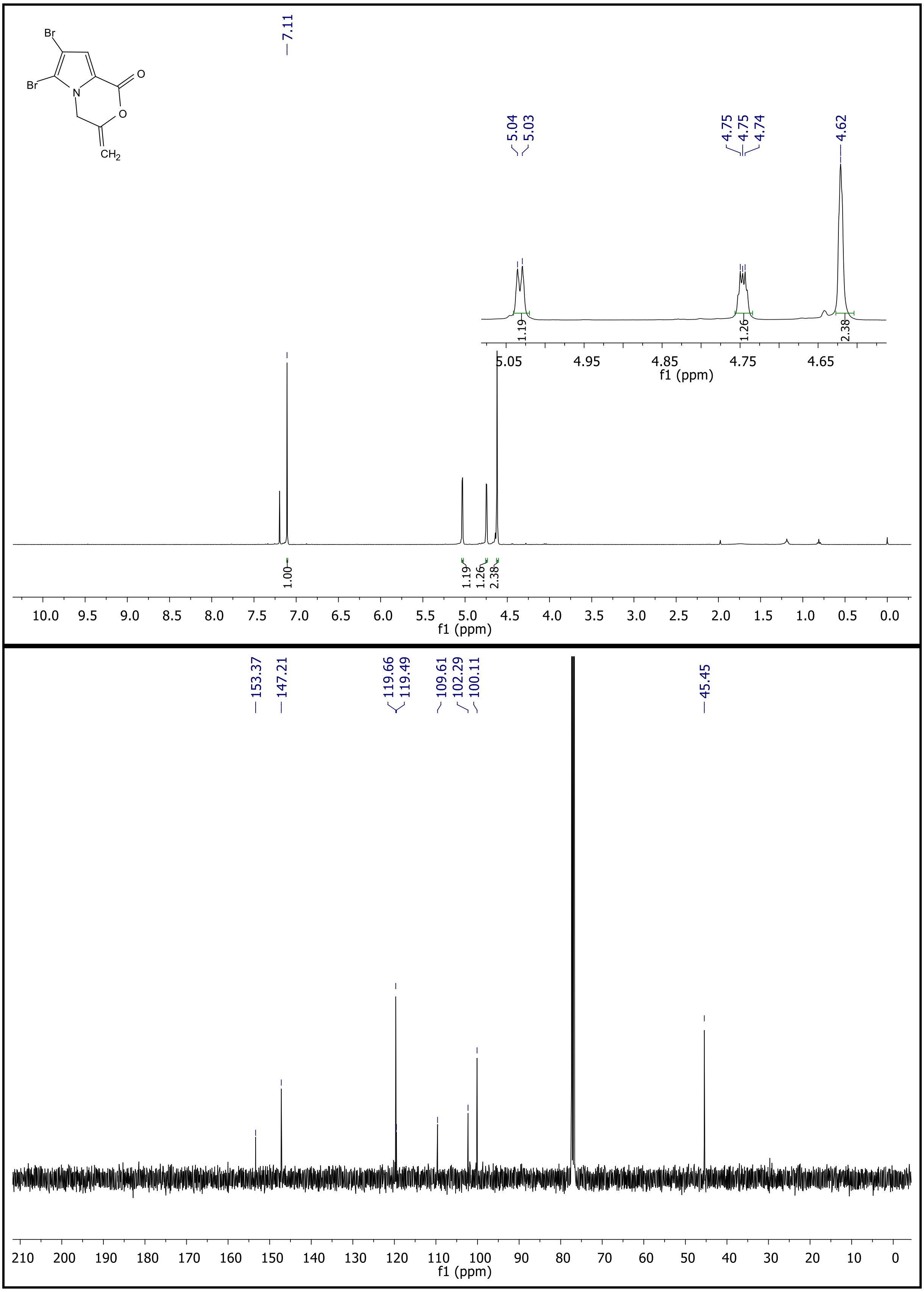

Figure 15: ^{1}H and ^{13}C NMR Spectra of **22** in $CDCl_3$

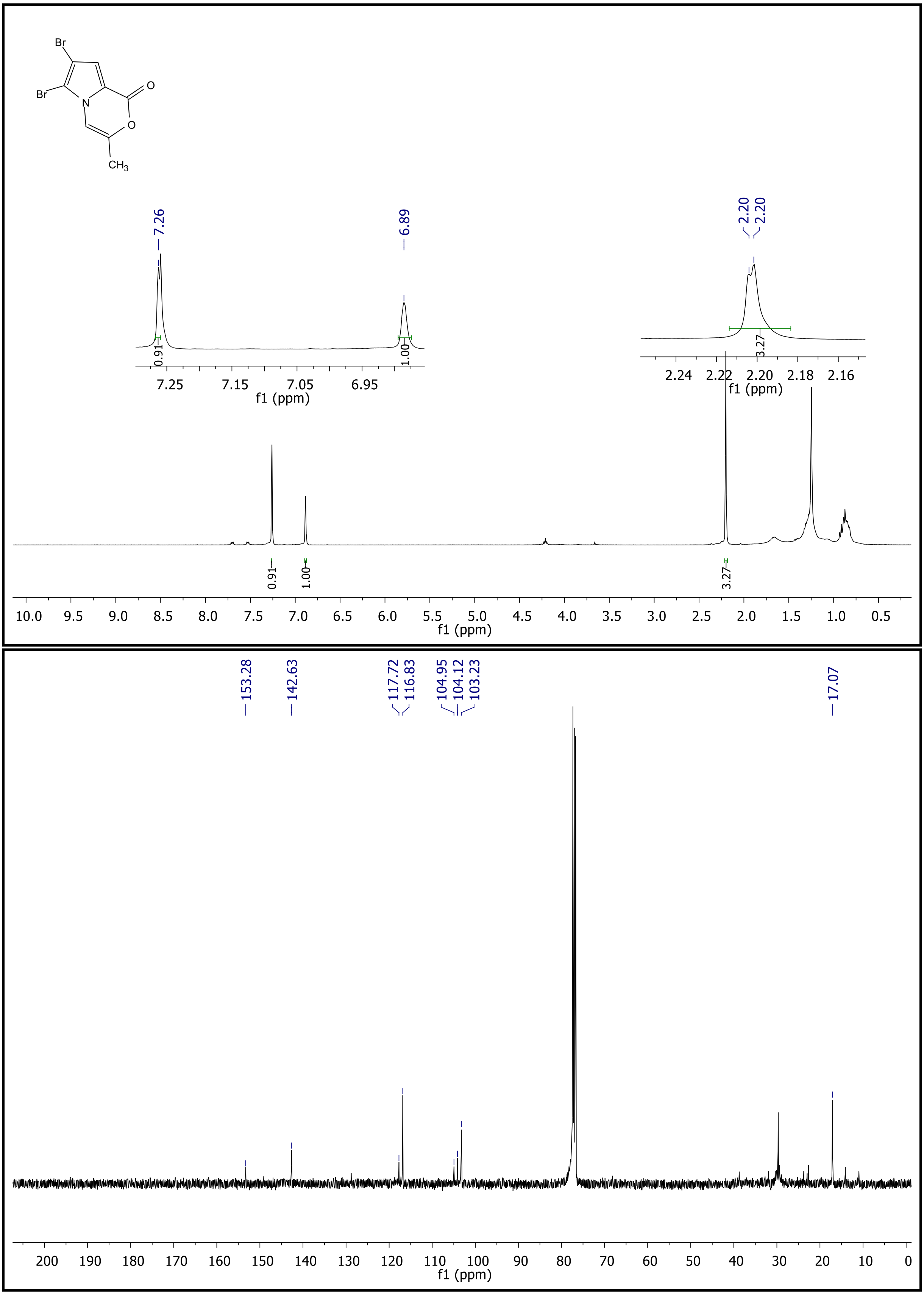

Figure 16: ^{1}H and ^{13}C NMR Spectra of **23** in $CDCl_3$

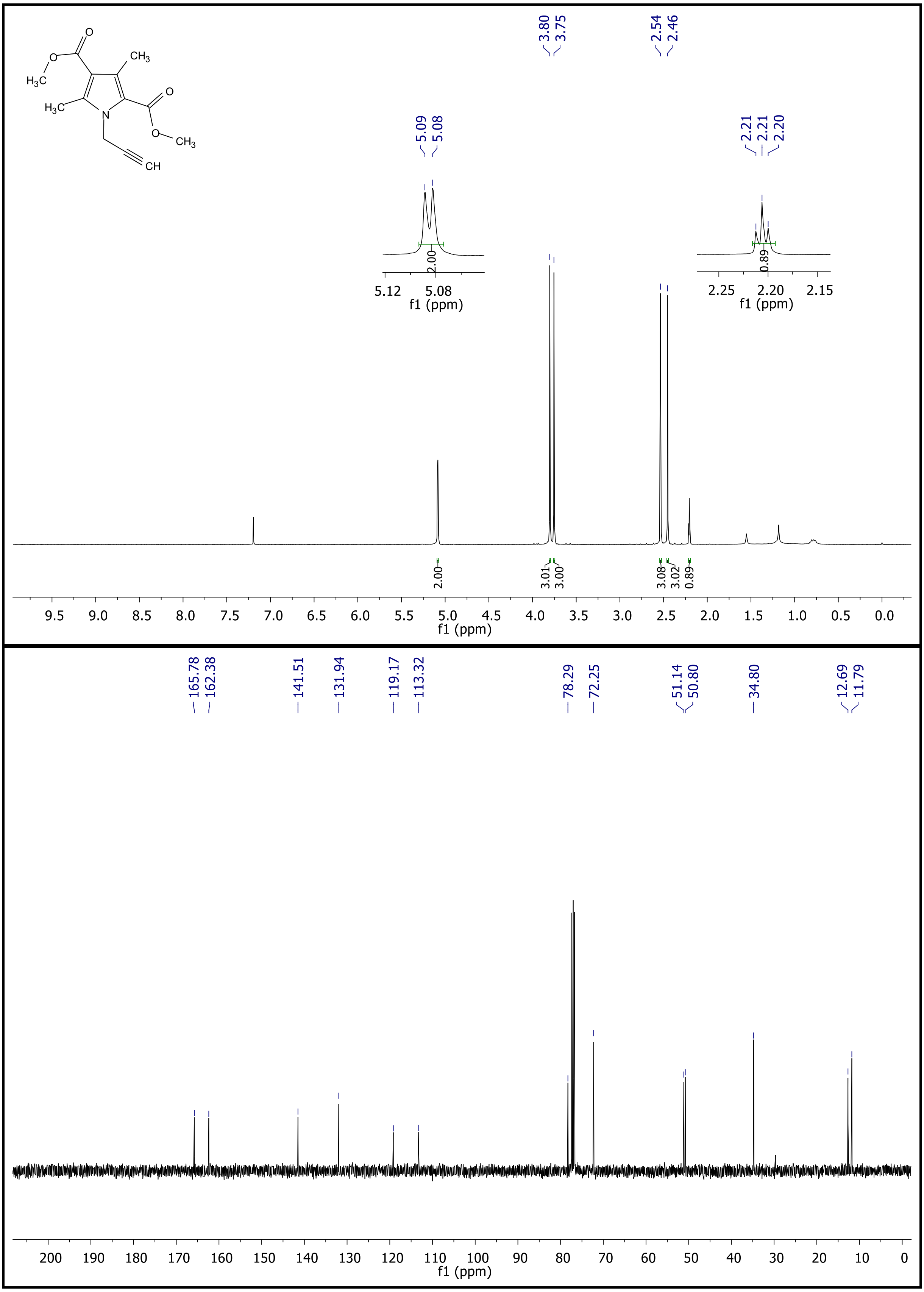

Figure 17: ^{1}H and ^{13}C NMR Spectra of **24** in $CDCl_3$

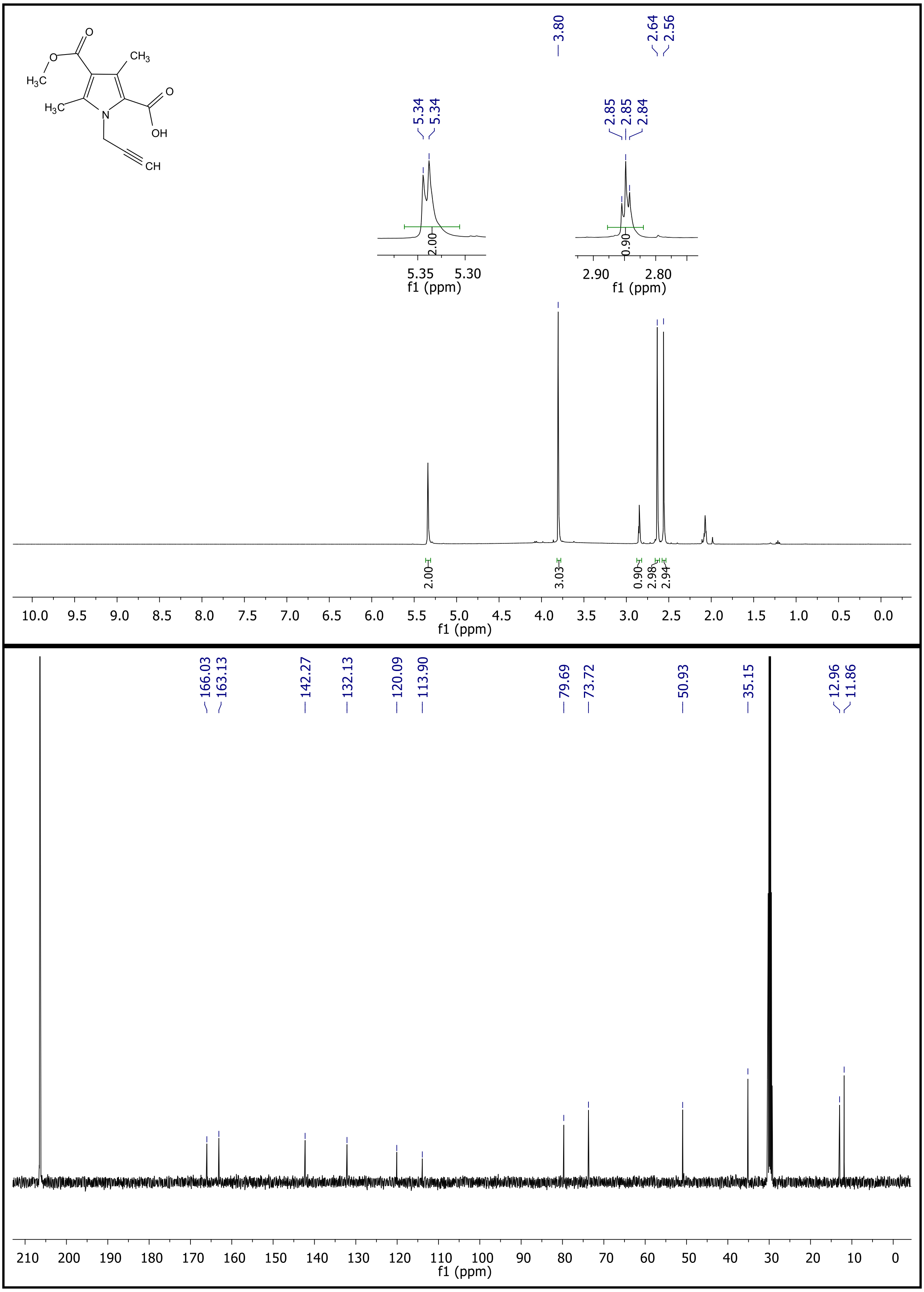

Figure 18: ^{1}H and ^{13}C NMR Spectra of **25** in CD_3COCD_3

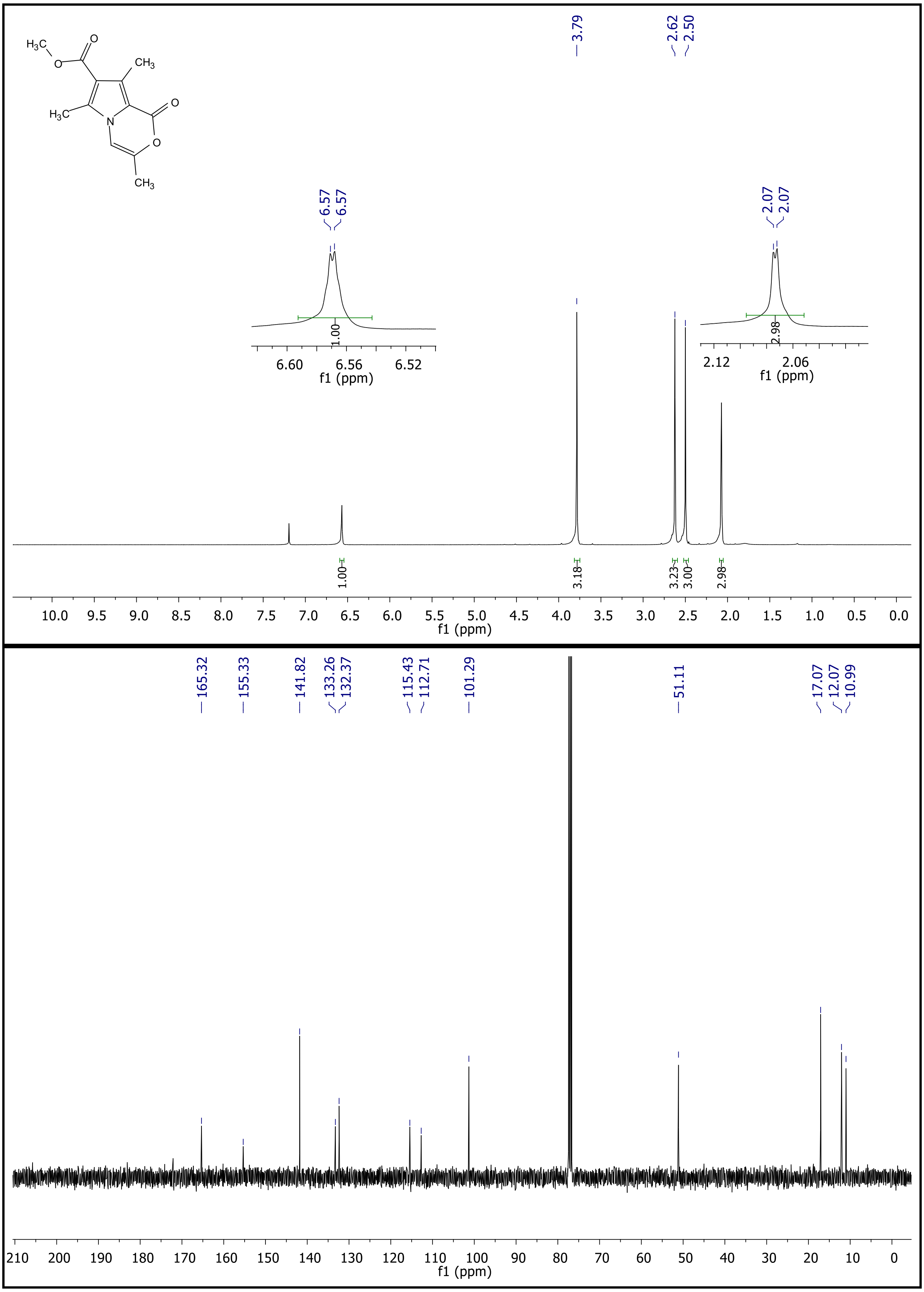

Figure 19: ^{1}H and ^{13}C NMR Spectra of **27** in $CDCl_3$

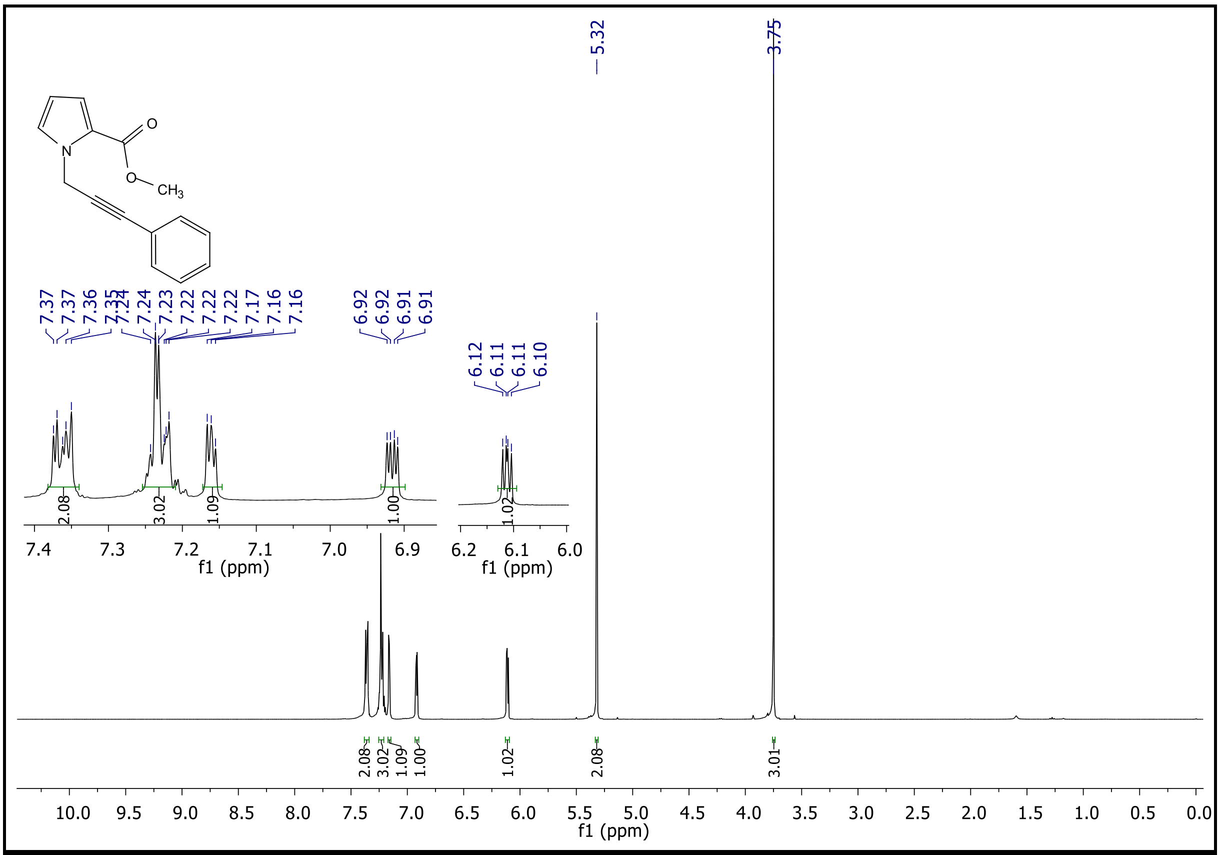

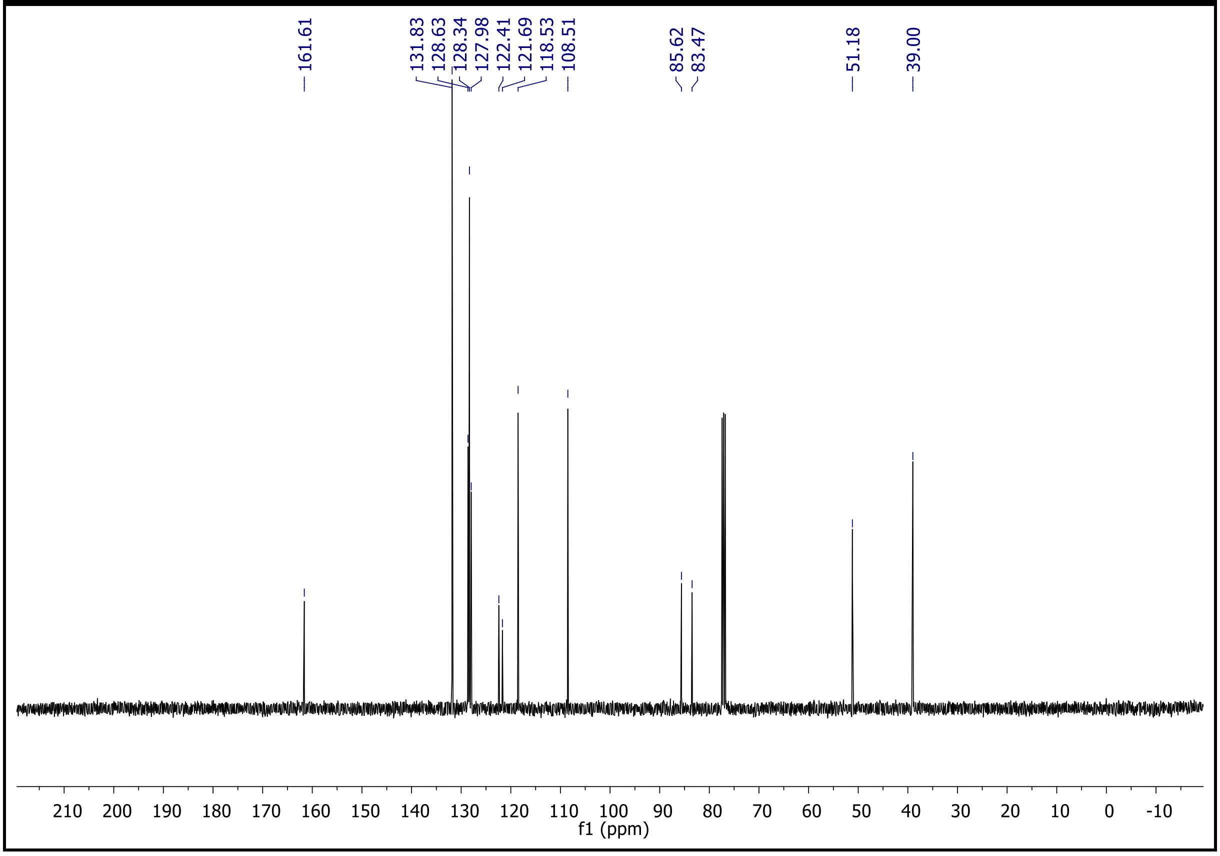

Figure 20: ^{1}H and ^{13}C NMR Spectra of **28** in $CDCl_3$

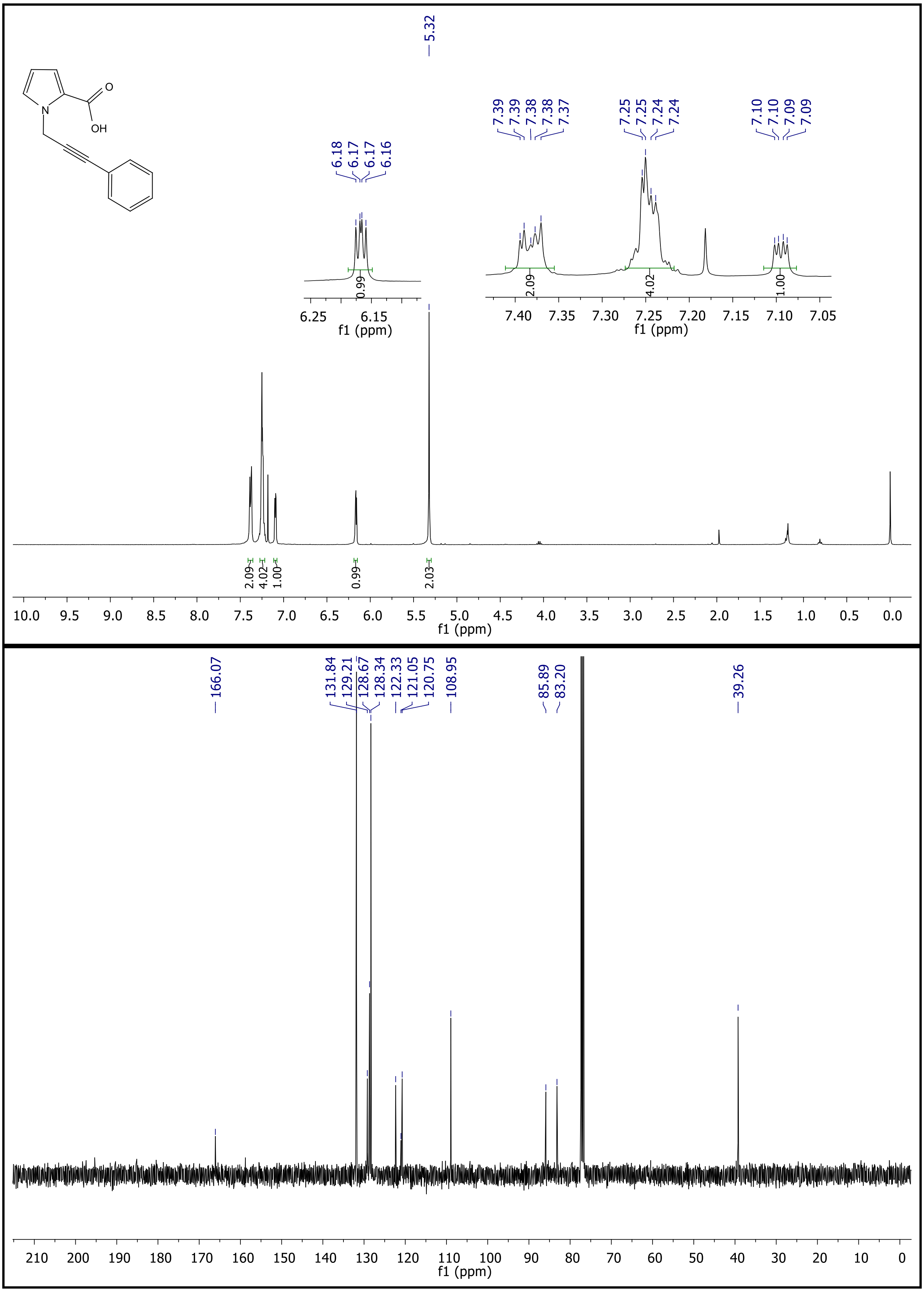

Figure 21: ^{1}H and ^{13}C NMR Spectra of **29** in $CDCl_3$

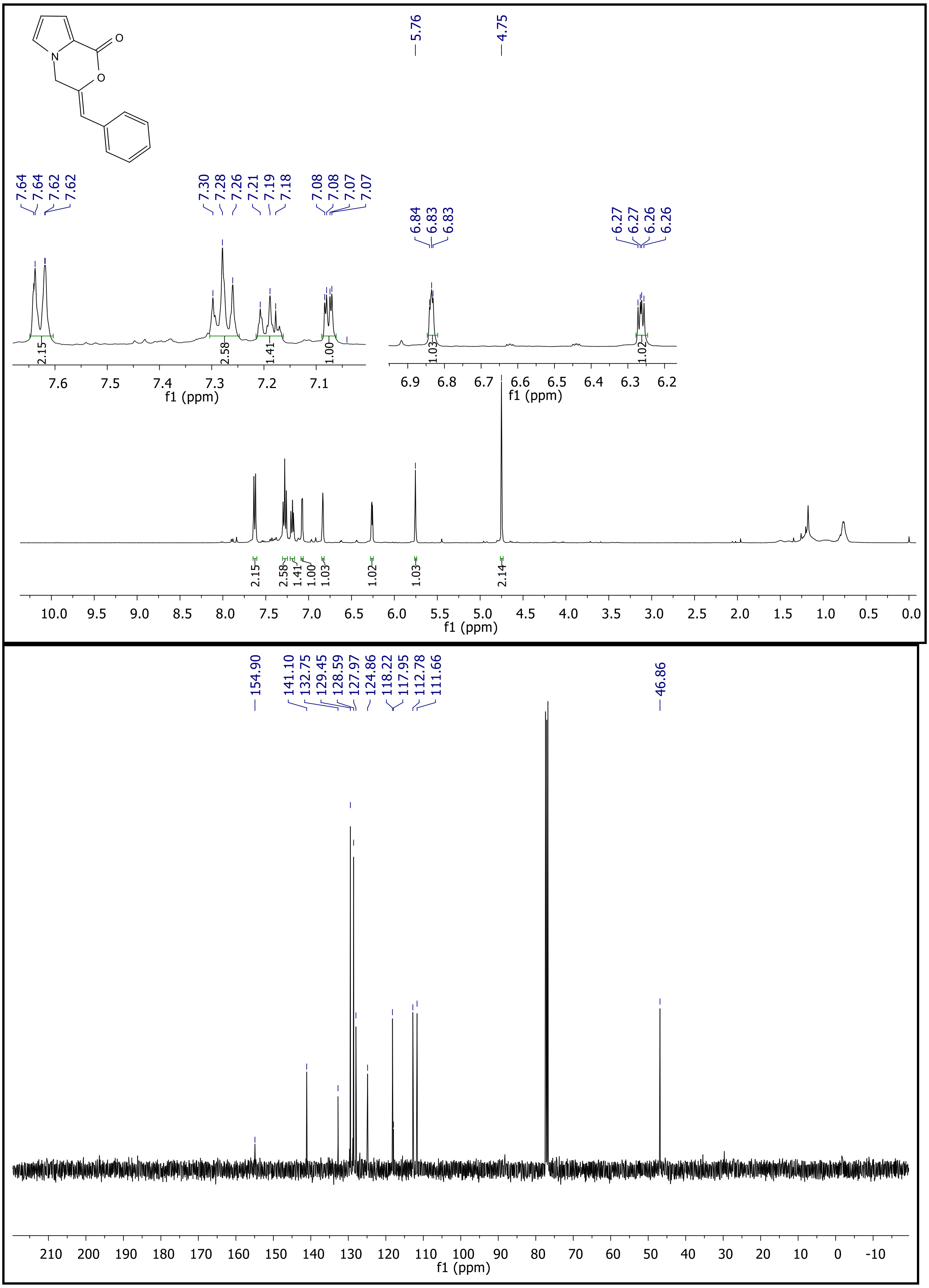

Figure 22: ^{1}H and ^{13}C NMR Spectra of **30** in $CDCl_3$

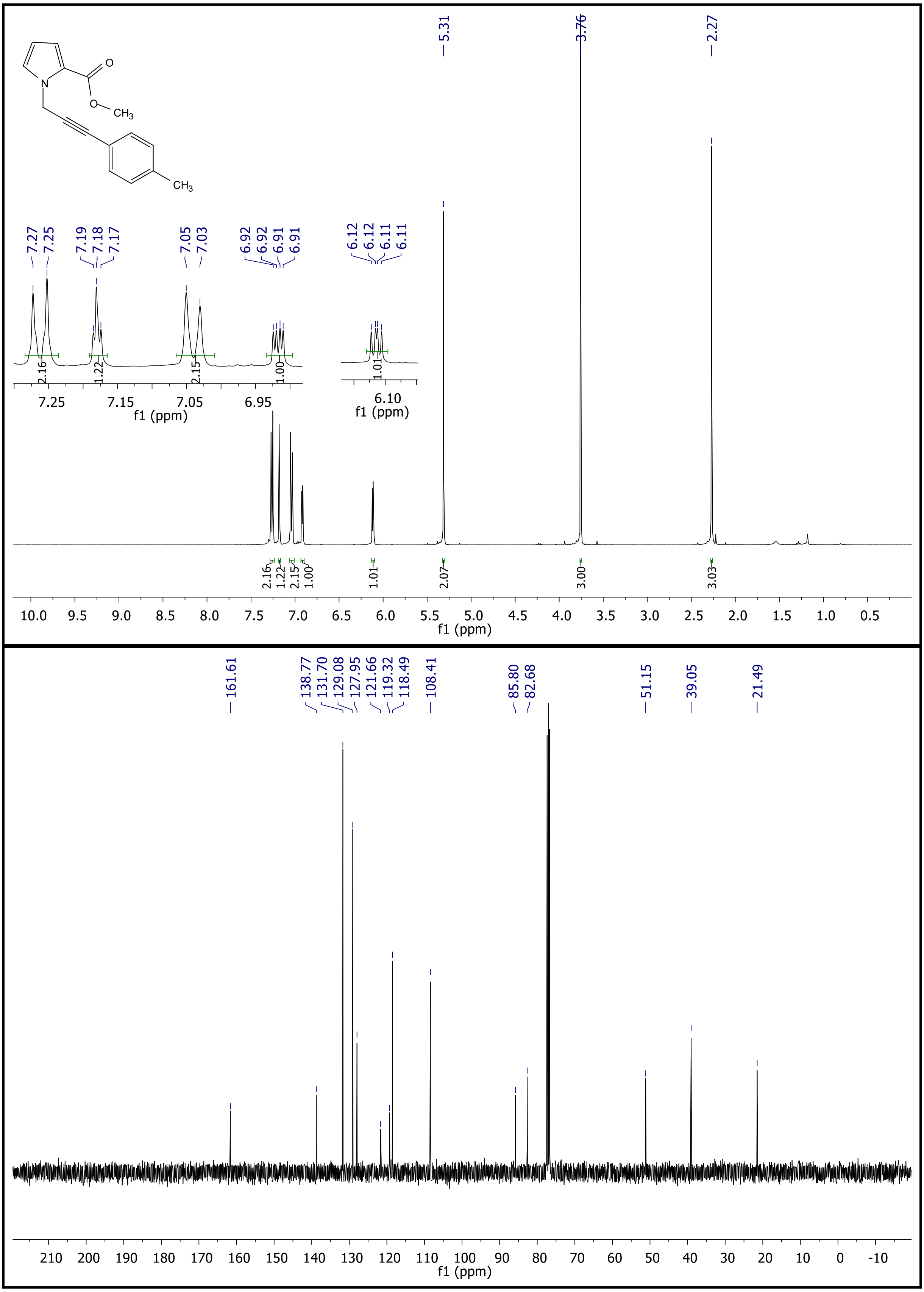

Figure 23: ^{1}H and ^{13}C NMR Spectra of **32** in $CDCl_3$

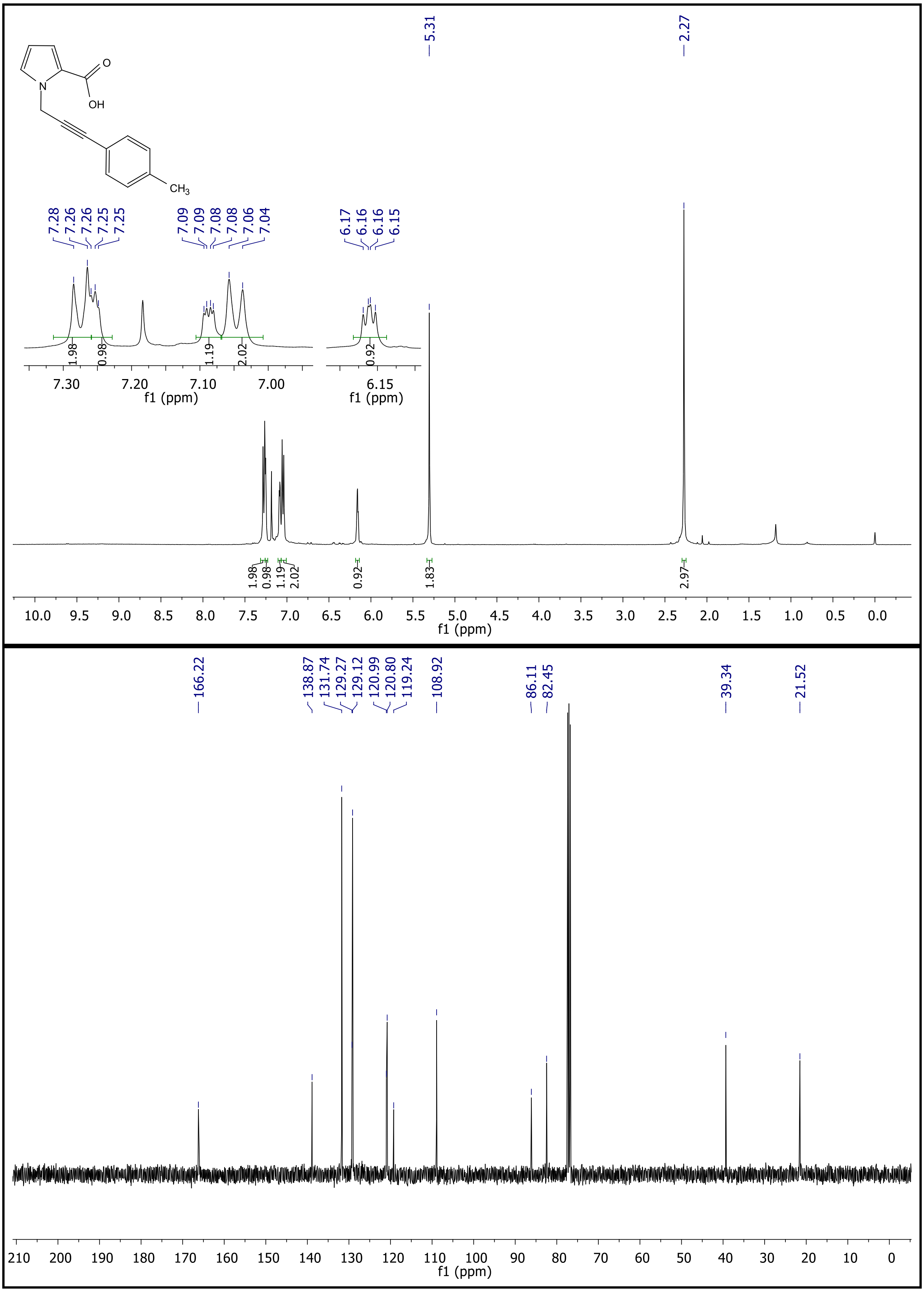

Figure 24: ^{1}H and ^{13}C NMR Spectra of **33** in $CDCl_3$

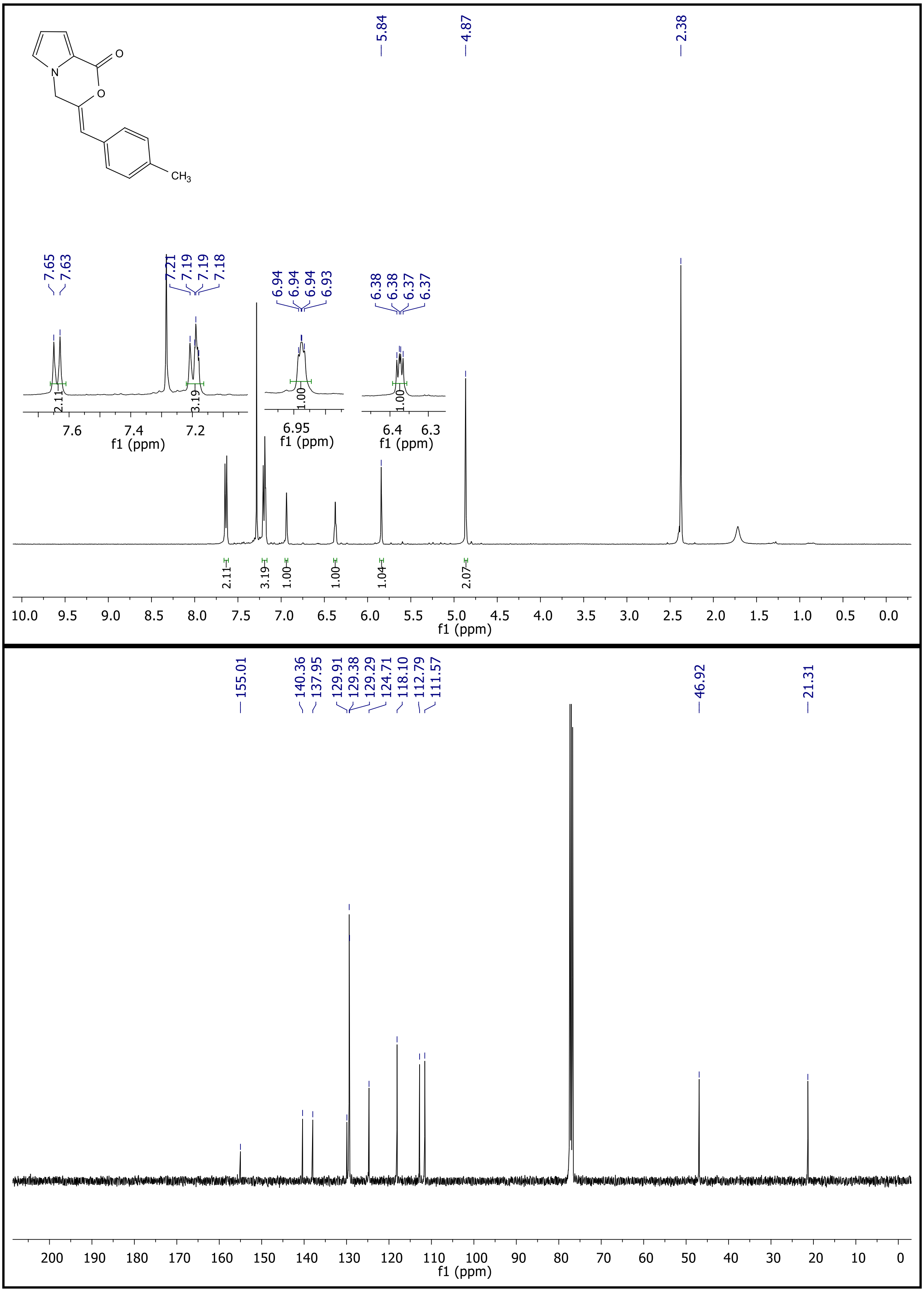

Figure 25: ^{1}H and ^{13}C NMR Spectra of **34** in $CDCl_3$

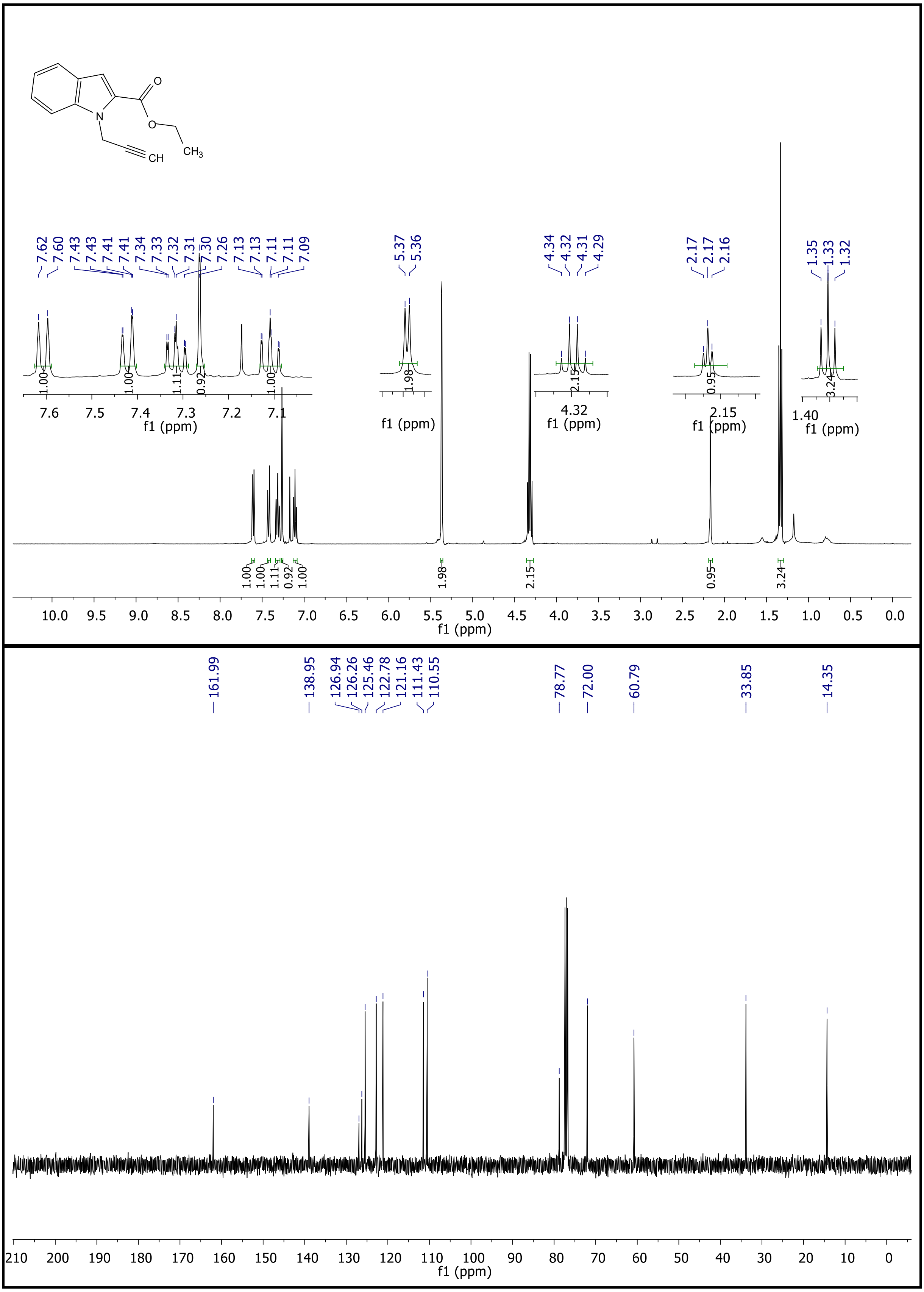

Figure 26: ^{1}H and ^{13}C NMR Spectra of **36** in $CDCl_3$

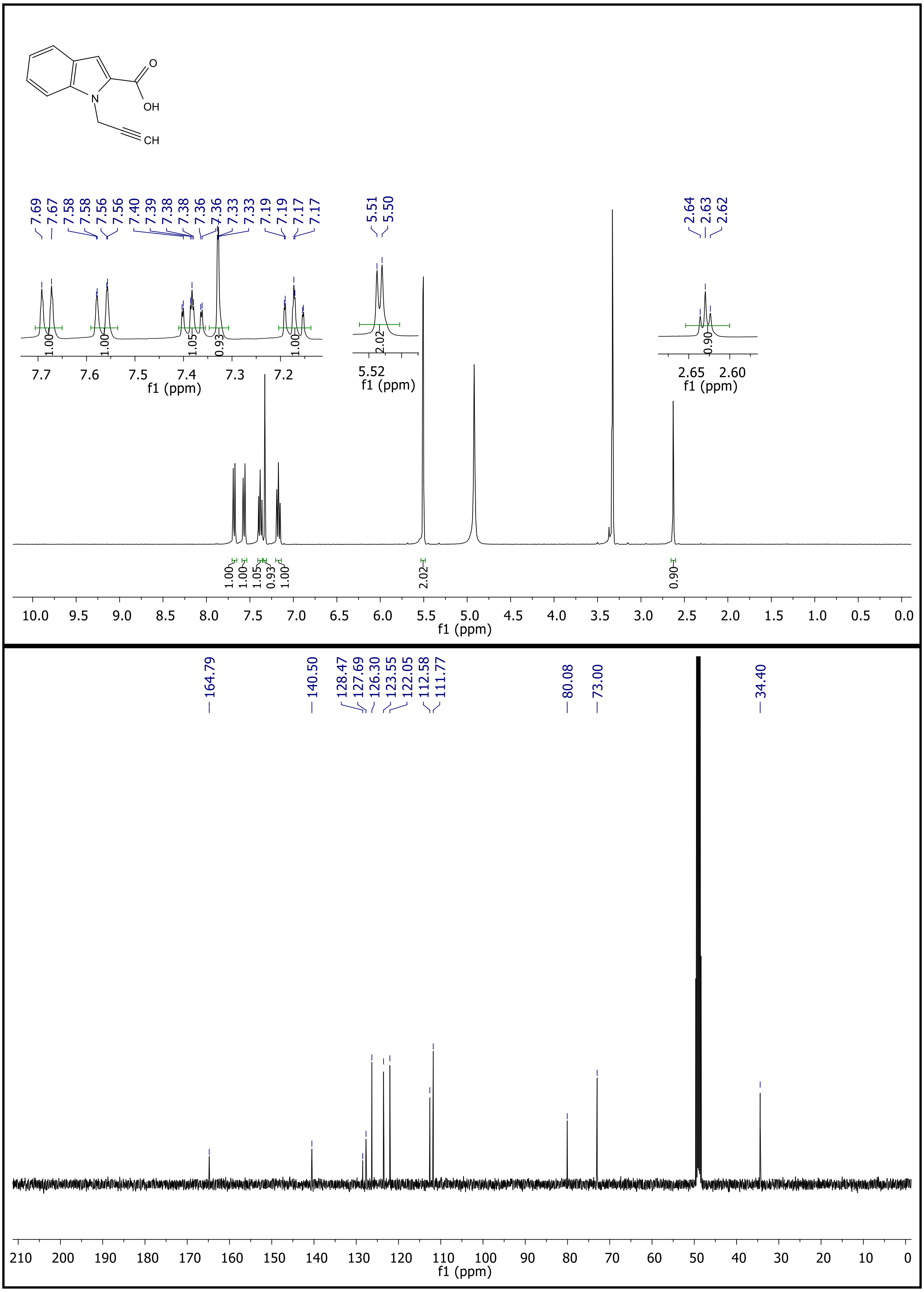

Figure 27: ^{1}H and ^{13}C NMR Spectra of **37** in CD_3OD

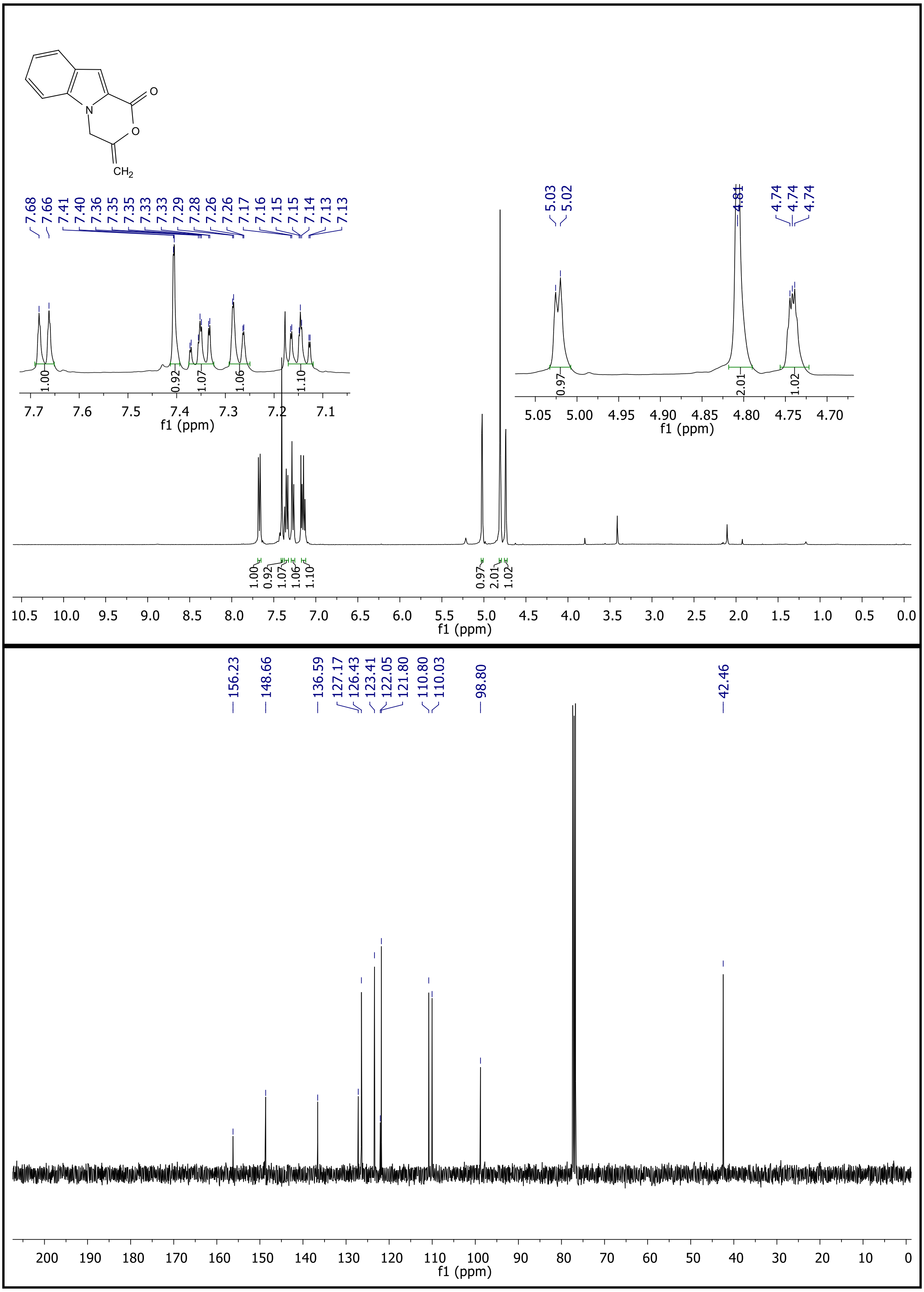

Figure 28: ^{1}H and ^{13}C NMR Spectra of **38** in $CDCl_3$

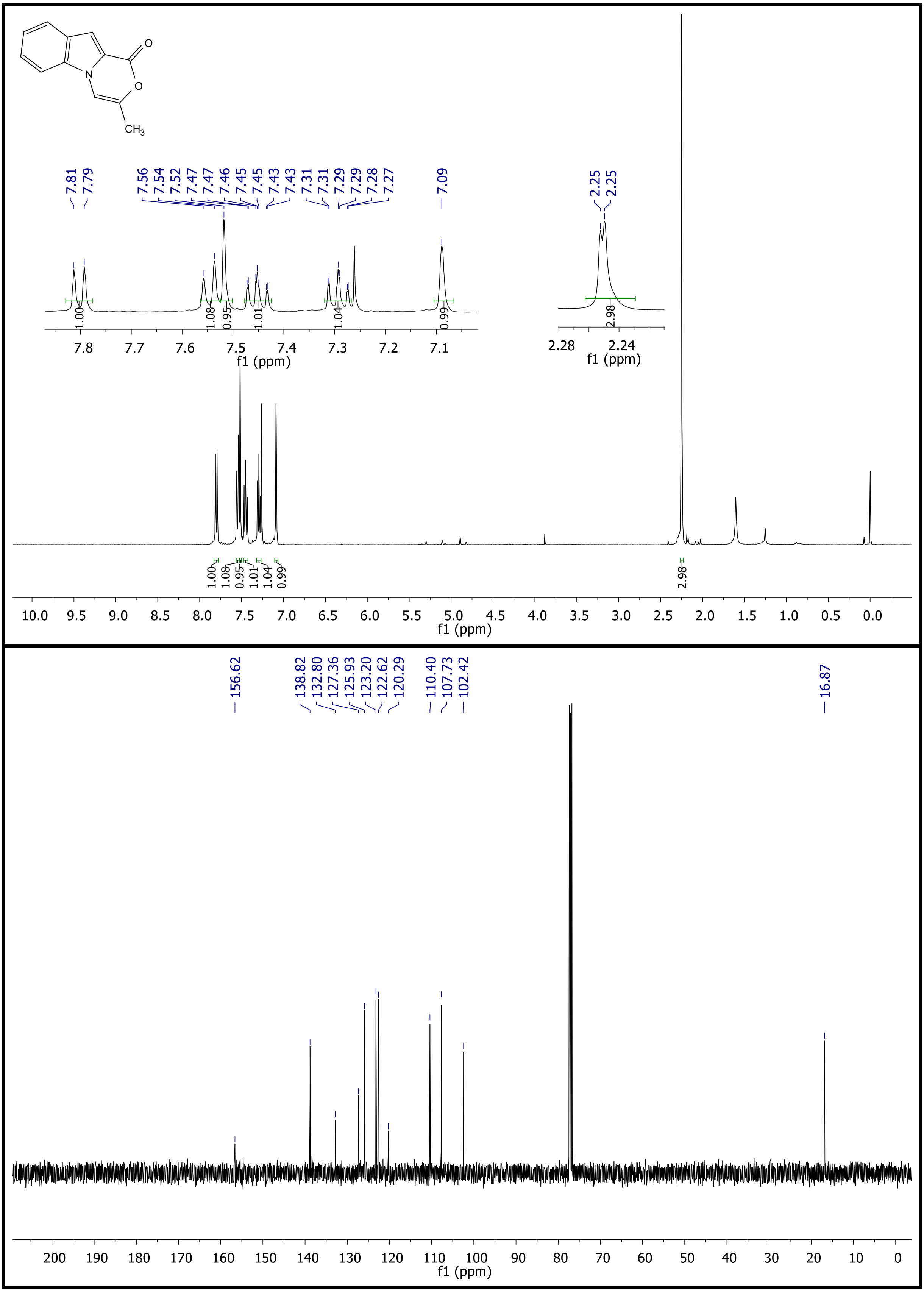

Figure 29: ^{1}H and ^{13}C NMR Spectra of **39** in $CDCl_3$

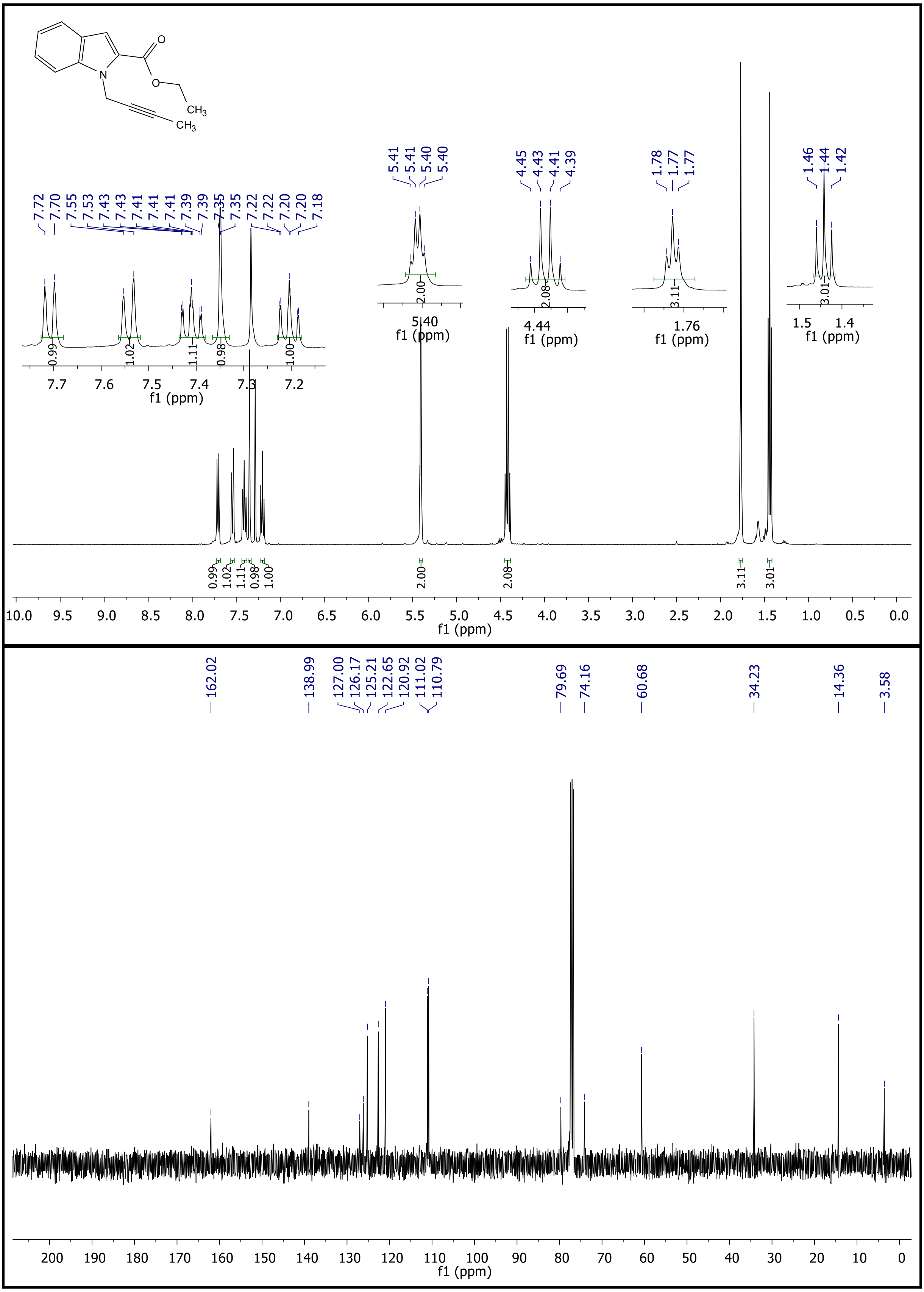

Figure 30: ^{1}H and ^{13}C NMR Spectra of **40** in $CDCl_3$

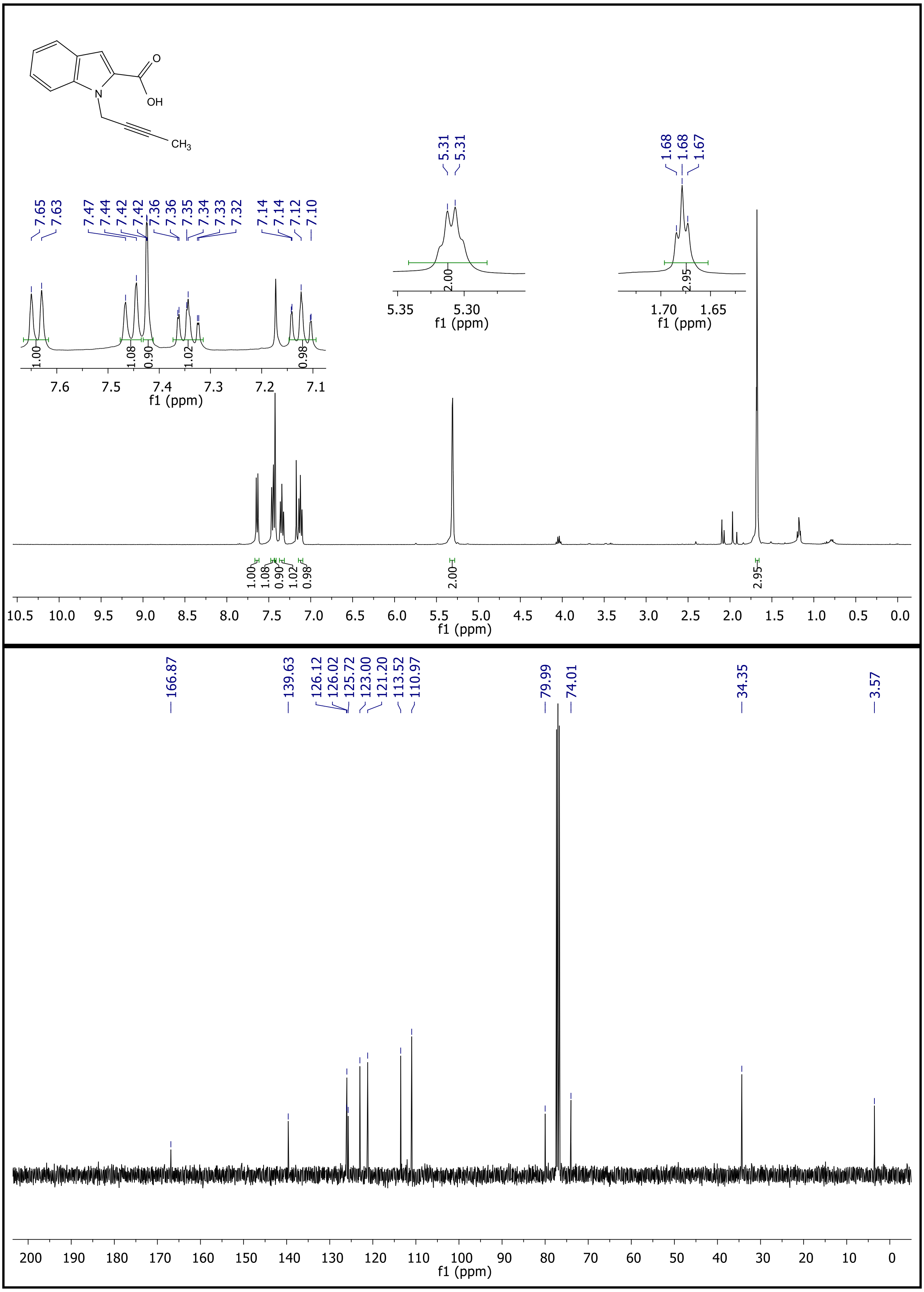

Figure 31: ^{1}H and ^{13}C NMR Spectra of **41** in $CDCl_3$

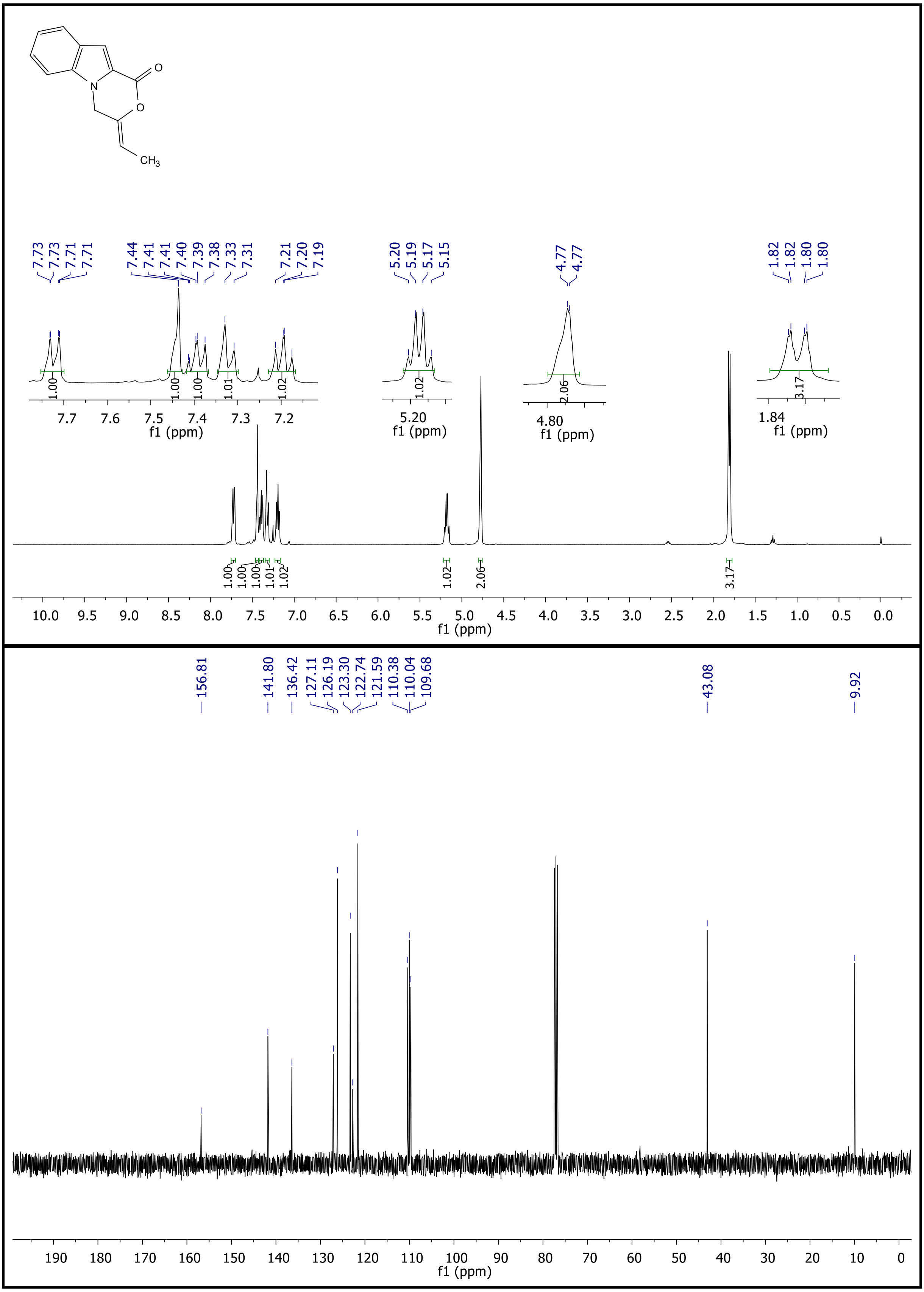

Figure 32: ^{1}H and ^{13}C NMR Spectra of **42** in $CDCl_3$

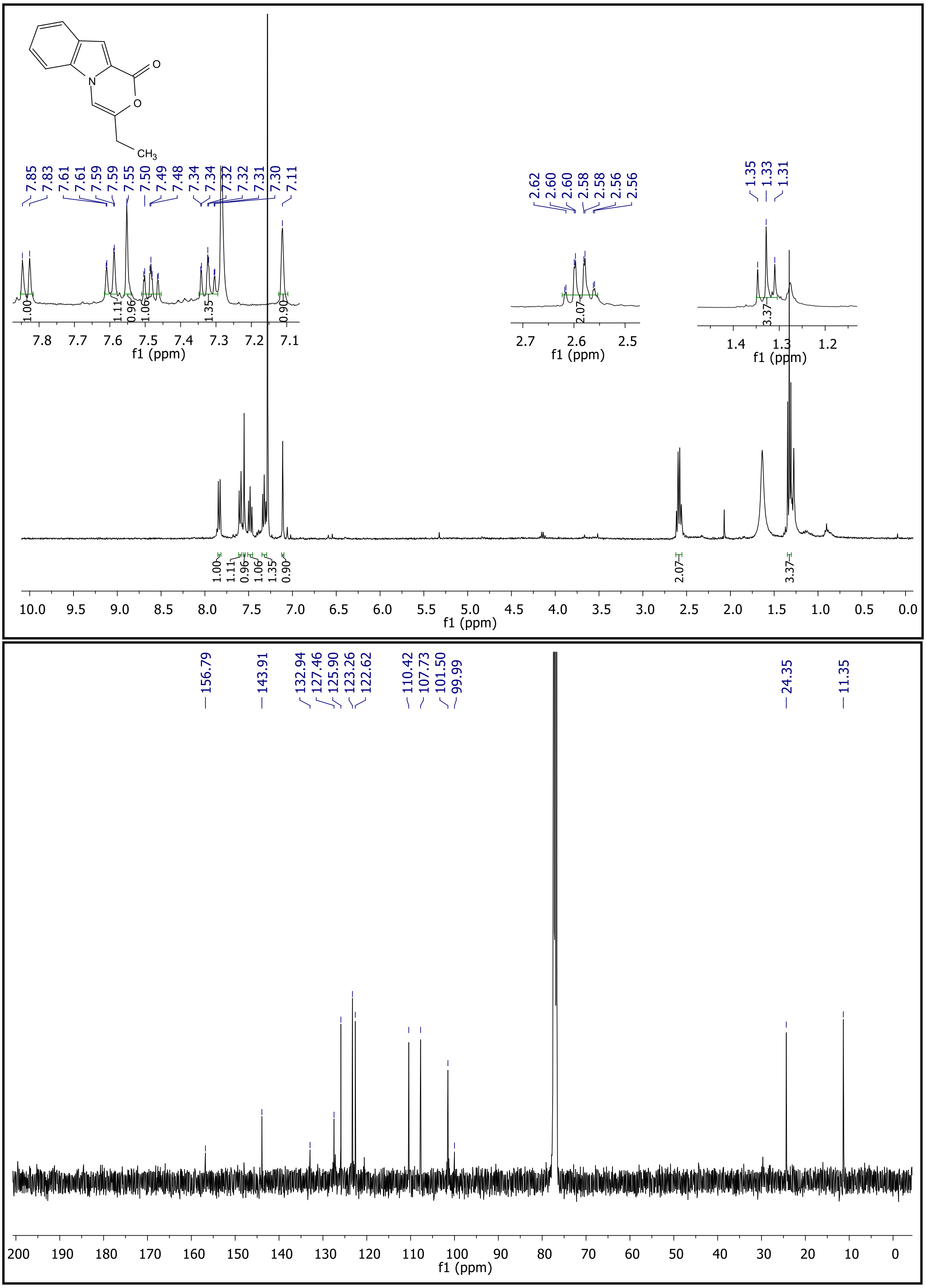

Figure 33: ^{1}H and ^{13}C NMR Spectra of **43** in $CDCl_3$

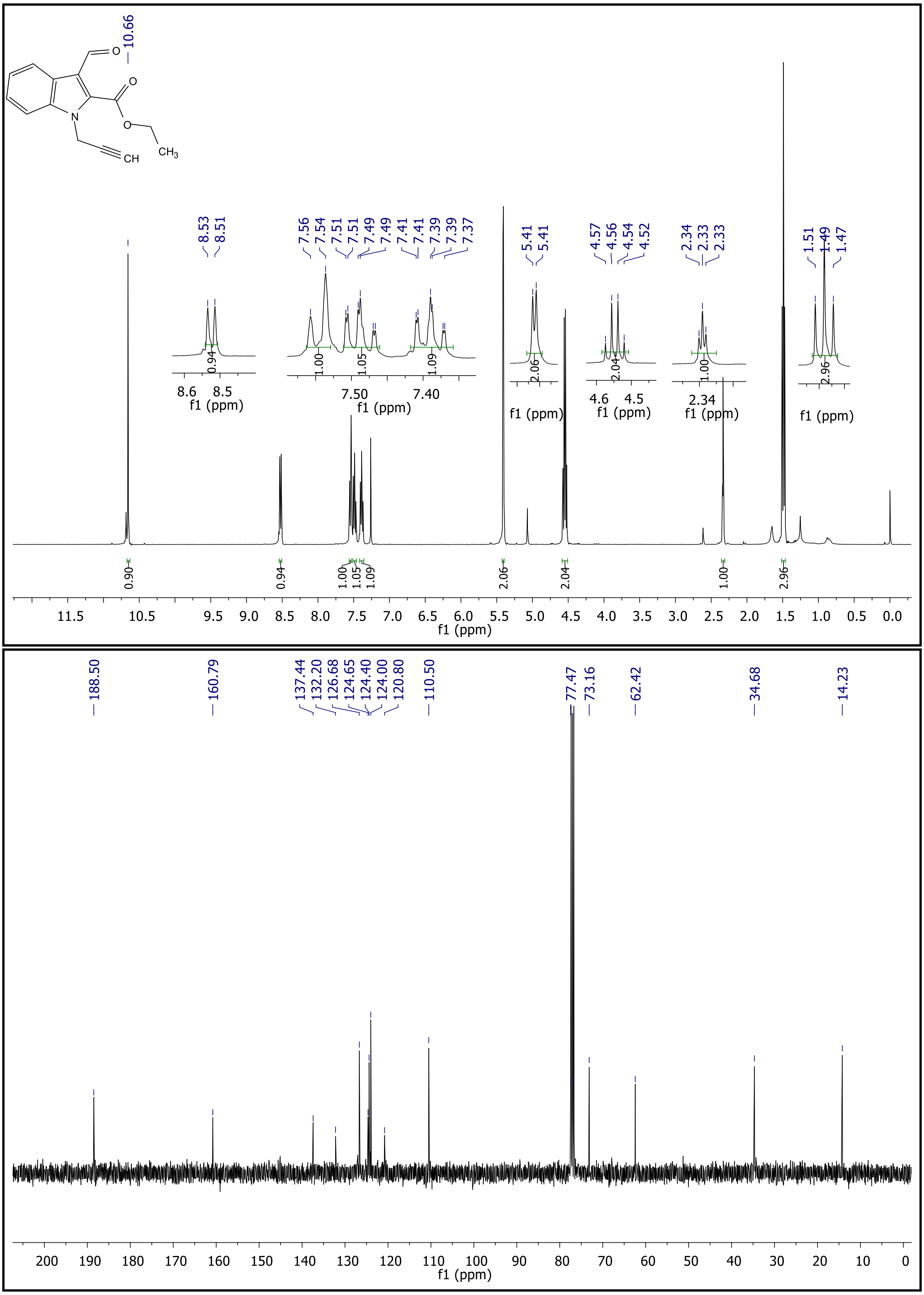

Figure 34: ^{1}H and ^{13}C NMR Spectra of **44** in $CDCl_3$

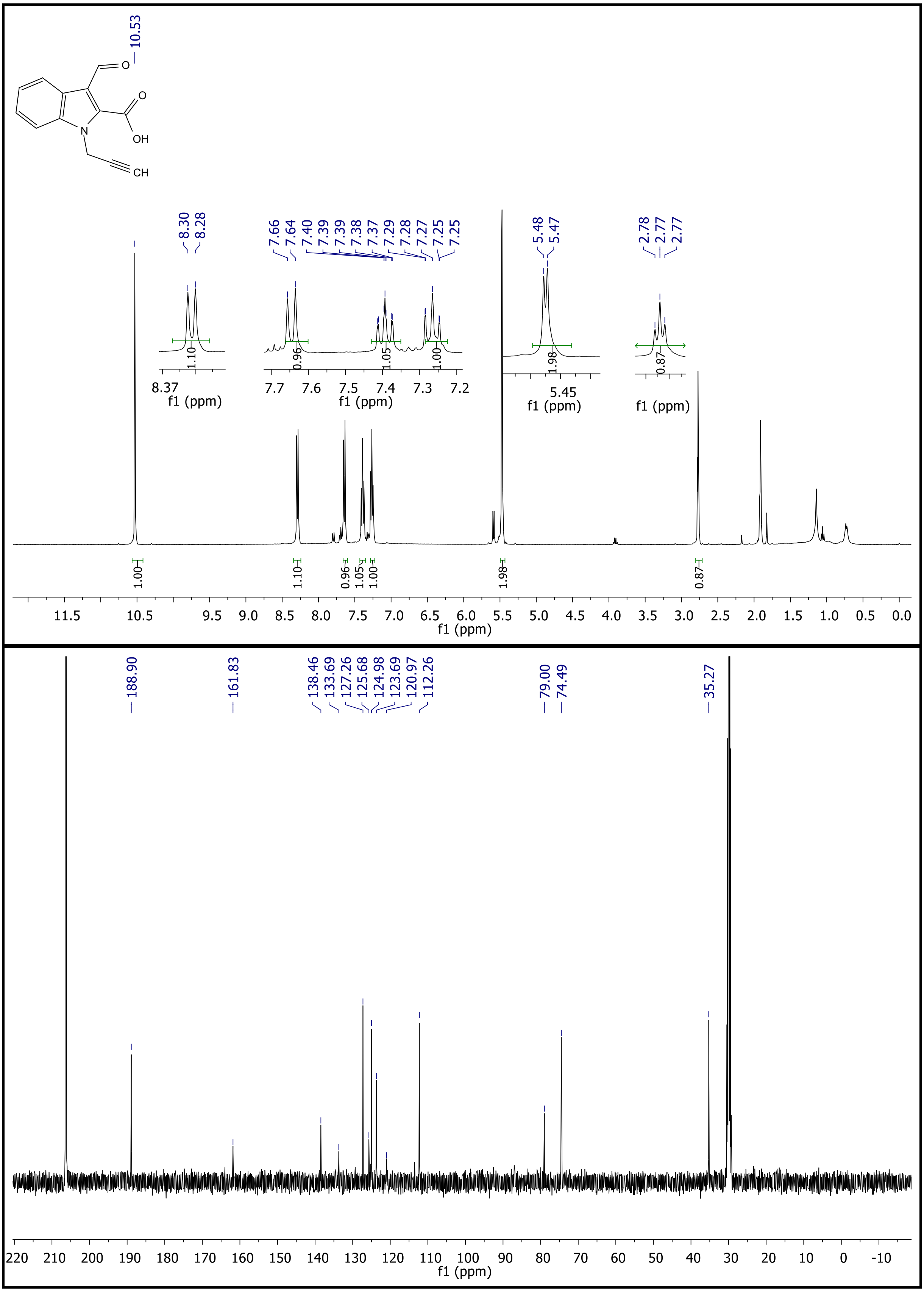

Figure 35: ^{1}H and ^{13}C NMR Spectra of **45** in CD_3COCD_3

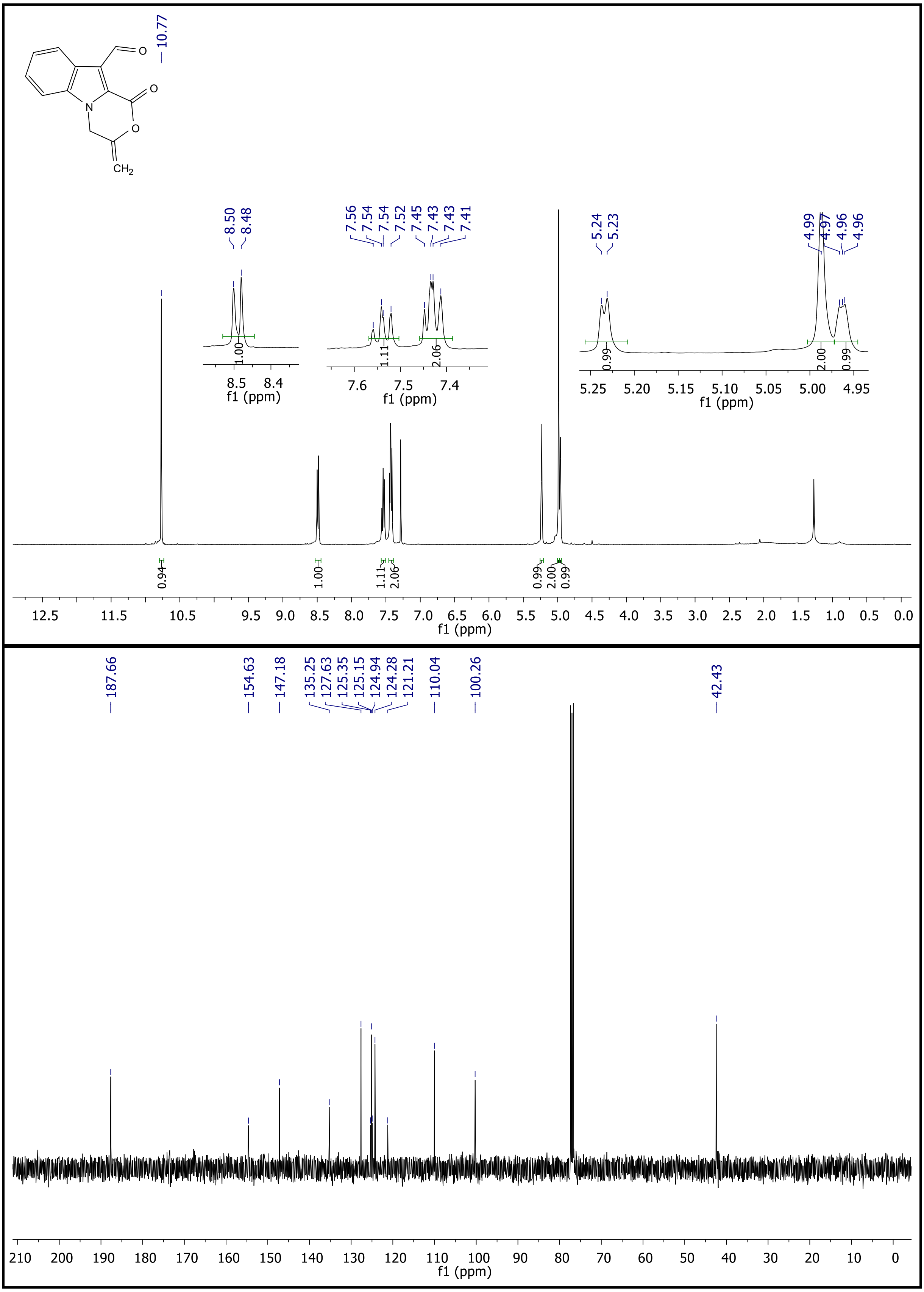

Figure 36: ^{1}H and ^{13}C NMR Spectra of **46** in $CDCl_3$

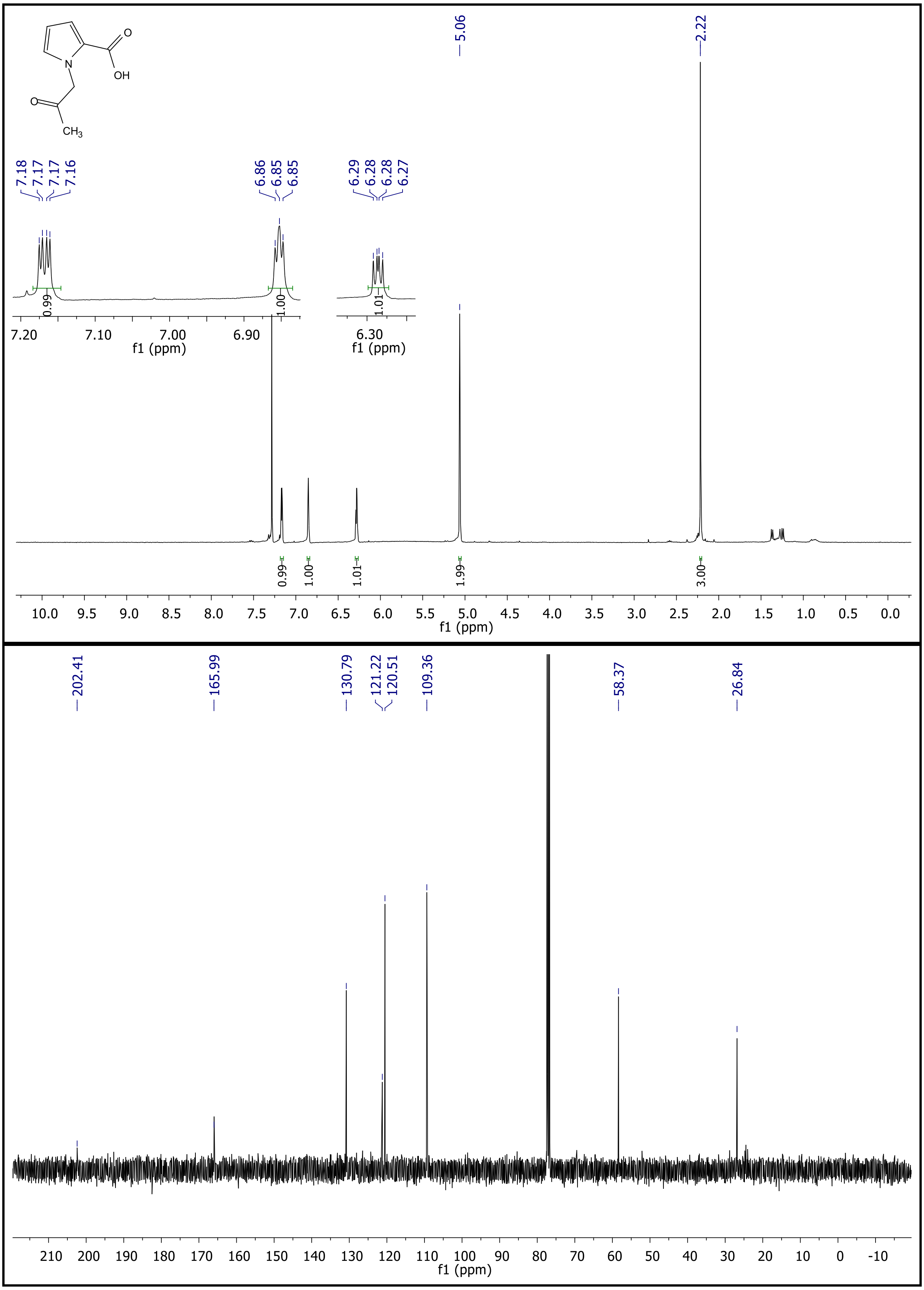

Figure 37: ^{1}H and ^{13}C NMR Spectra of **48** in $CDCl_3$

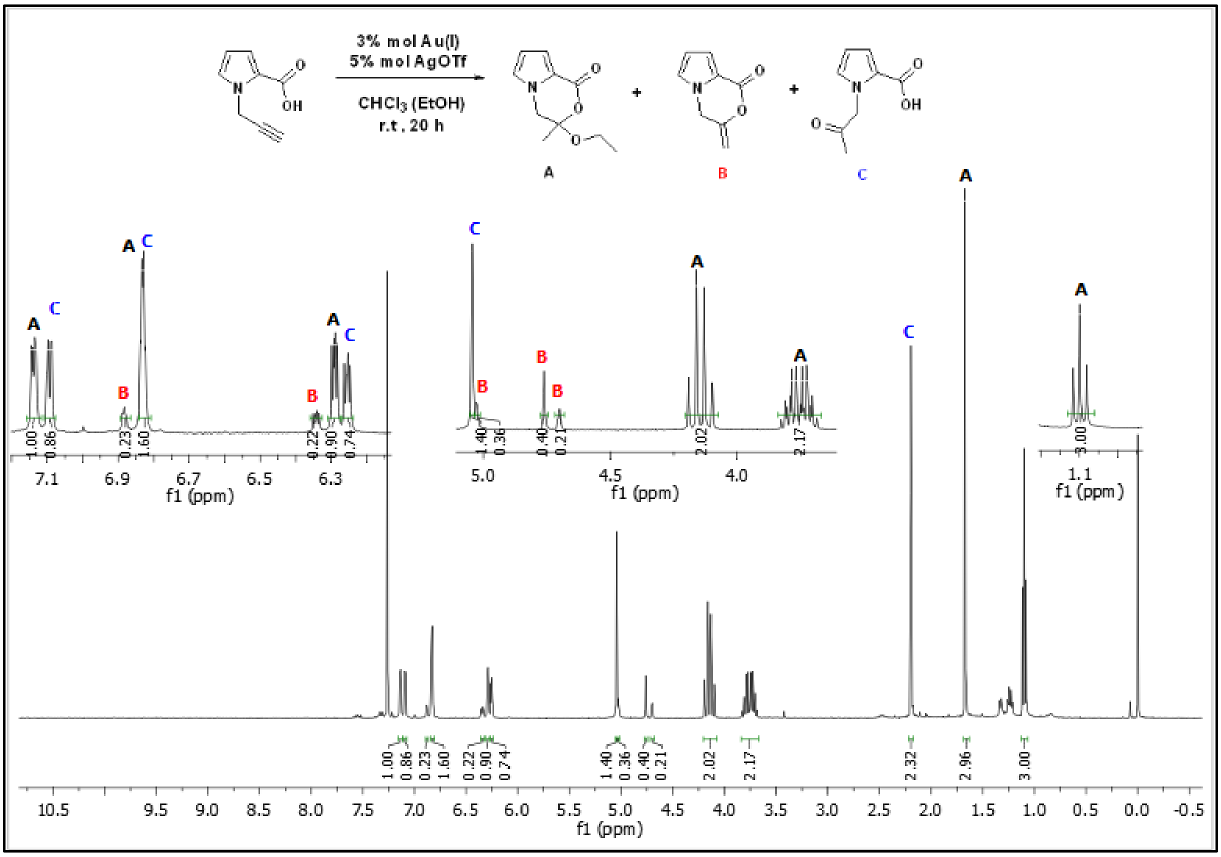

Figure 38: ^{1}H NMR Spectra of the reaction of **15** with gold(I) in chloroform in the presence of ethanol.

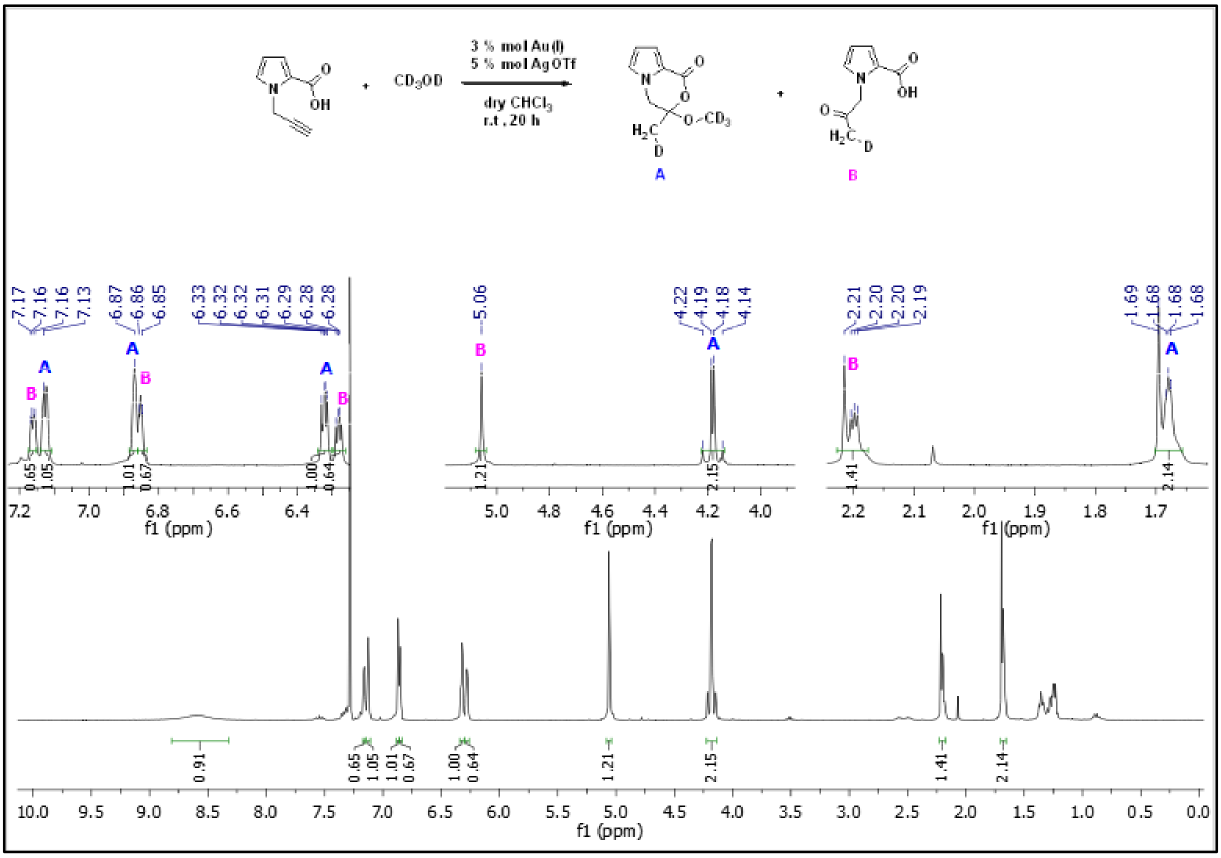

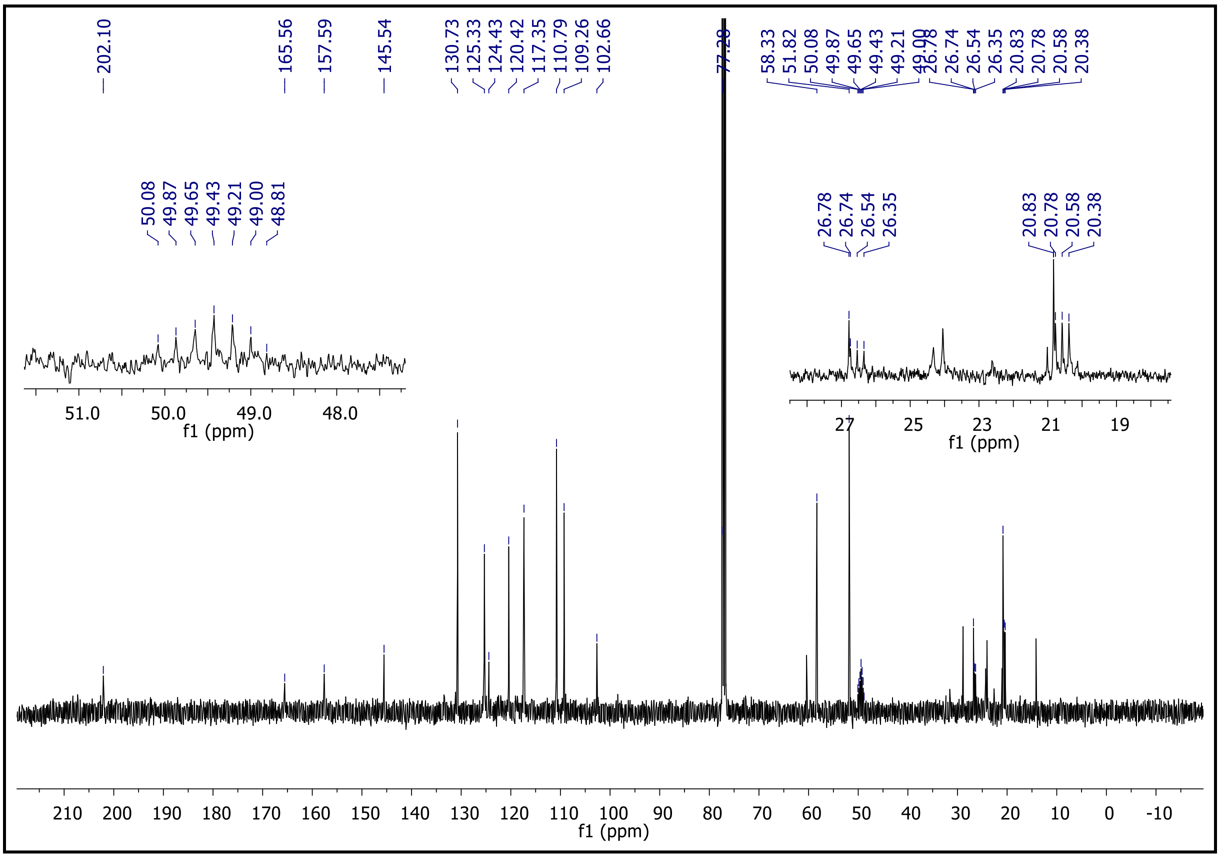

Figure 39: ^{1}H and ^{13}C NMR Spectra of the reaction of **15** with gold(I) in the presence of CD_3OD.

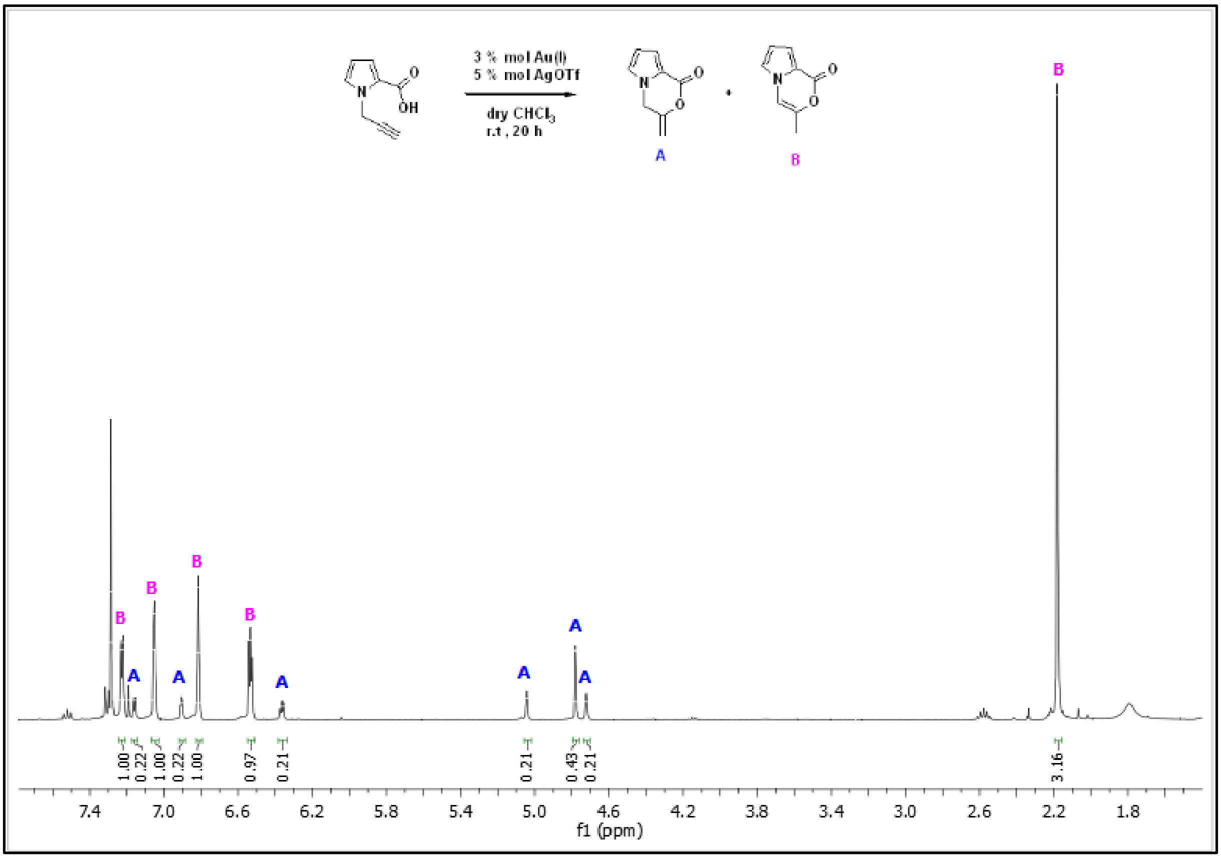

Figure 40: ^{1}H NMR Spectra of the reaction of **15** with gold(I) in chloroform

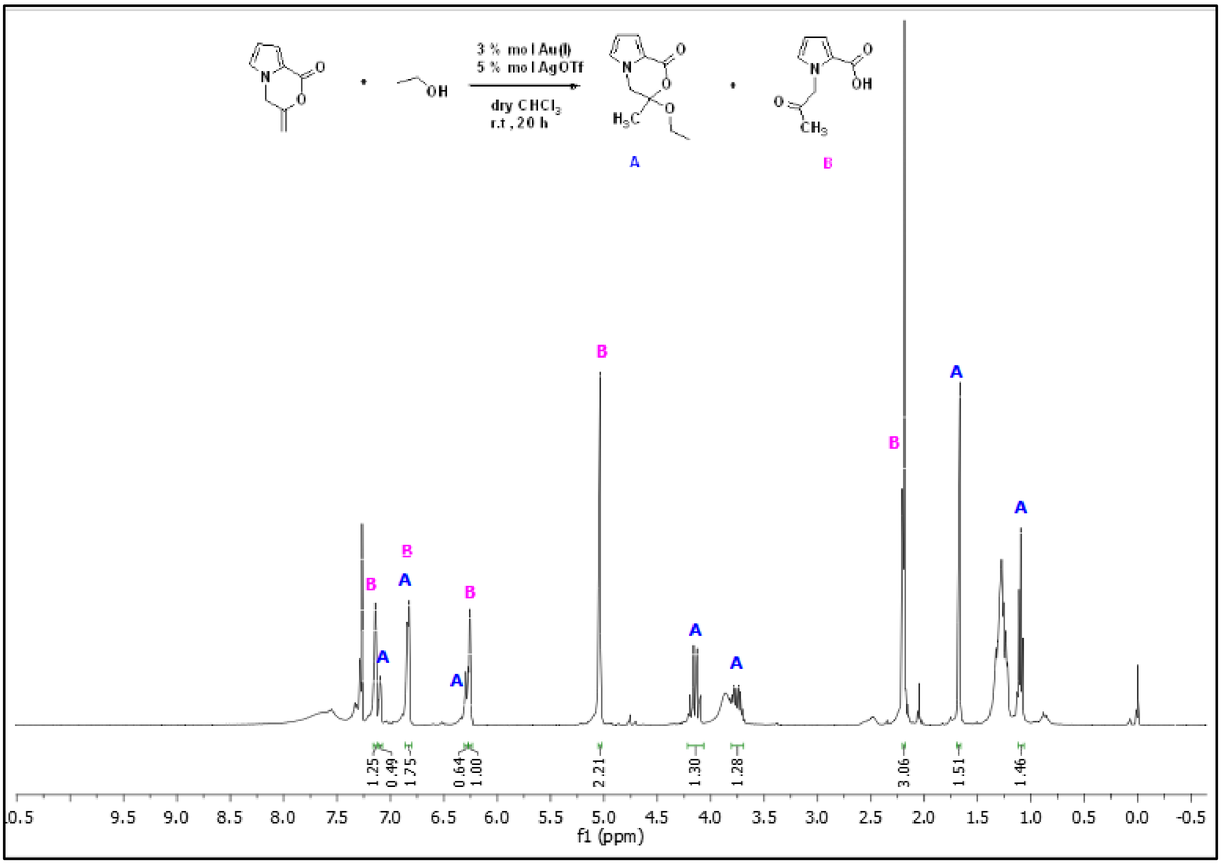

Figure 41: ^{1}H NMR Spectra of the reaction of **7** with gold(I) in the presence of EtOH

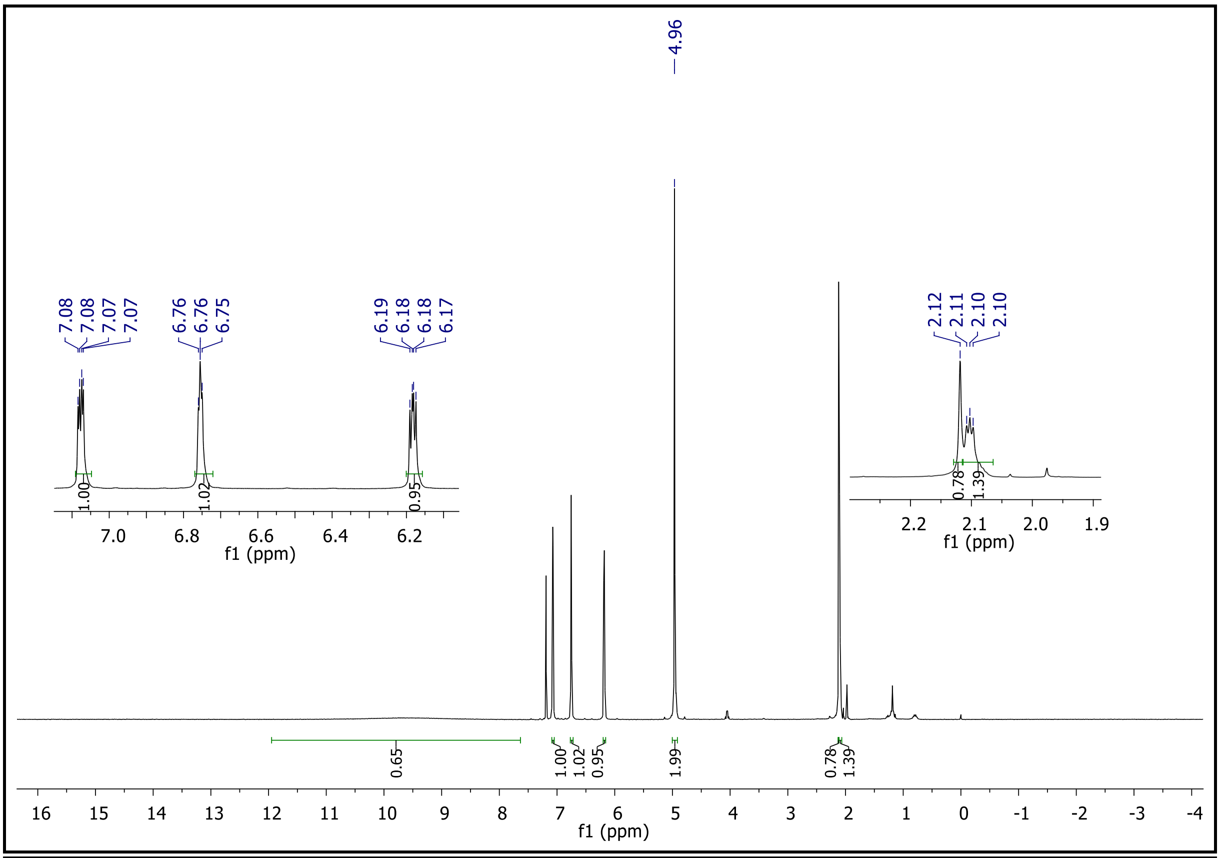

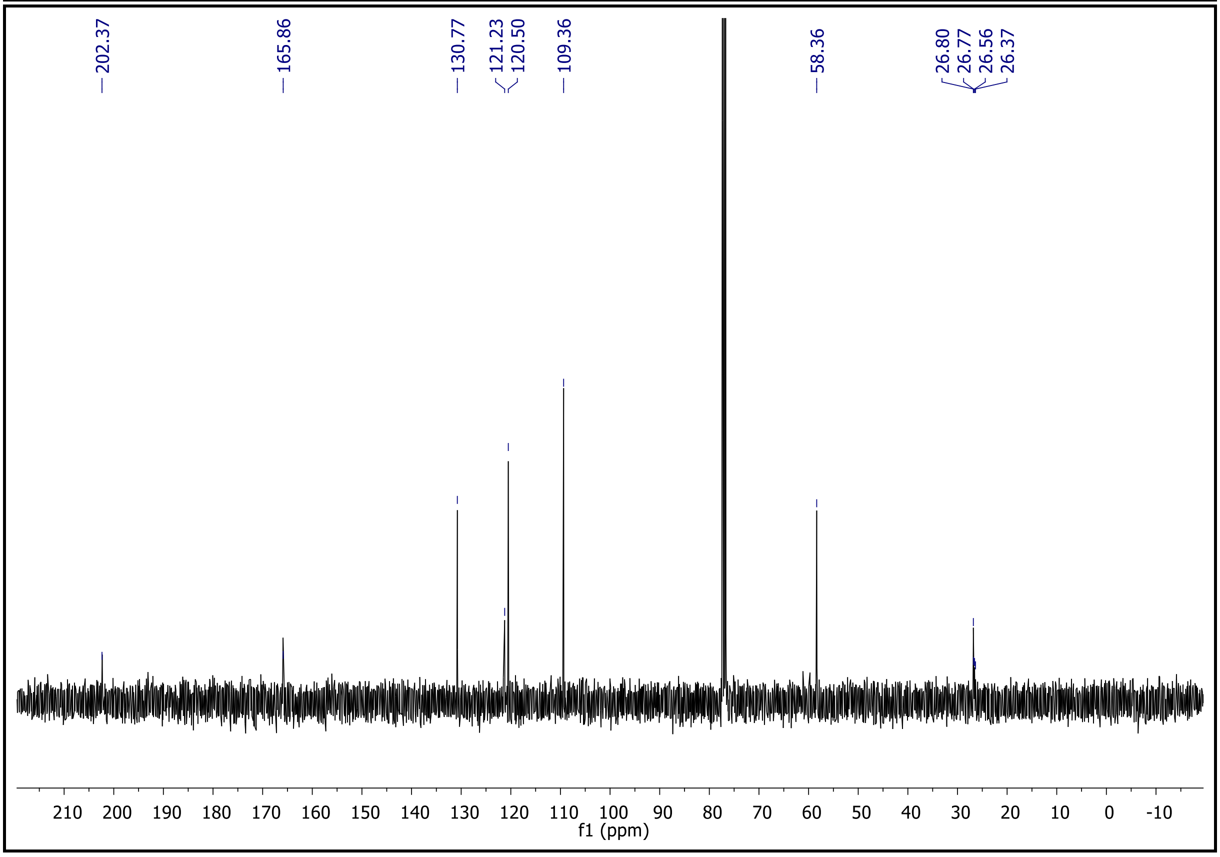

Figure 42: ^{1}H and ^{13}C NMR Spectra of **50a** and **50b** in $CDCl_3$
